# A consolidated analysis of the physiologic and molecular responses induced under acid stress in the legume-symbiont model-soil bacterium *Sinorhizobium meliloti*

**DOI:** 10.1038/srep29278

**Published:** 2016-07-11

**Authors:** W. O. Draghi, M. F. Del Papa, C. Hellweg, S. A. Watt, T. F. Watt, A. Barsch, M. J. Lozano, A. Lagares, M. E. Salas, J. L. López, F. J. Albicoro, J. F. Nilsson, G. A. Torres Tejerizo, M. F. Luna, M. Pistorio, J. L. Boiardi, A. Pühler, S. Weidner, K. Niehaus, A. Lagares

**Affiliations:** 1 IBBM - Instituto de Biotecnología y Biología Molecular, CONICET - Departamento de Ciencias Biológicas, Facultad de Ciencias Exactas, Universidad Nacional de La Plata, calles 47 y 115, 1900-La Plata, Argentina; 2 CeBiTec - Centrum für Biotechnologie, Universität Bielefeld, Bielefeld, Germany; 3 Laboratorio de Bioquímica, Microbiología e Interacciones Biológicas en el Suelo, Departamento de Ciencia y Tecnología, Universidad Nacional de Quilmes, Roque Sáenz Peña 352, Bernal B1876BXD, Buenos Aires, Argentina; 4 CINDEFI - Centro de Investigación y Desarrollo en Fermentaciones Industriales, CONICET - Departamento de Química, Facultad de Ciencias Exactas, Universidad Nacional de La Plata, calles 47 y 115, 1900-La Plata, Argentina

## Abstract

Abiotic stresses in general and extracellular acidity in particular disturb and limit nitrogen-fixing symbioses between rhizobia and their host legumes. Except for valuable molecular-biological studies on different rhizobia, no consolidated models have been formulated to describe the central physiologic changes that occur in acid-stressed bacteria. We present here an integrated analysis entailing the main cultural, metabolic, and molecular responses of the model bacterium *Sinorhizobium meliloti* growing under controlled acid stress in a chemostat. A stepwise extracellular acidification of the culture medium had indicated that *S. meliloti* stopped growing at ca. pH 6.0–6.1. Under such stress the rhizobia increased the O_2_ consumption per cell by more than 5-fold. This phenotype, together with an increase in the transcripts for several membrane cytochromes, entails a higher aerobic-respiration rate in the acid-stressed rhizobia. Multivariate analysis of global metabolome data served to unequivocally correlate specific-metabolite profiles with the extracellular pH, showing that at low pH the pentose-phosphate pathway exhibited increases in several transcripts, enzymes, and metabolites. Further analyses should be focused on the time course of the observed changes, its associated intracellular signaling, and on the comparison with the changes that operate during the sub lethal acid-adaptive response (ATR) in rhizobia.

Rhizobia are Gram-negative α- and β-proteobacteria that have the ability to fix atmospheric nitrogen in symbiotic association with legumes^1^,^2^ as well as with nonleguminous plants in a few known examples^3^. That capacity gives these associations a central role in the N cycle, thus making rhizobia also a subject of interest in agricultural-production systems^4^,^5^,^6^,^7^. The establishment of symbiosis in natural soils, however, is frequently limited by abiotic-stress conditions which affect that mutualistic interaction in different ways^8^. Thus, the acidity of soils limits nodulation and N_2_ fixation in many legume-rhizobia symbioses of agronomic interest^9^. Soil acidity is an extended edaphic condition in culturable lands over the entire globe^10^, and is known to be a major limiting circumstance for legume productivity. The increased concentration of hydrogen ions negatively affects at once the host plant^11^,^12^, the rhizobia^13^, and the symbiotic relationship^14^,^15^,^16^. Different stages in the interaction between rhizobia and the host roots have been reported to be affected by acidity, including the production of nodulation factors^14^,^17^,^18^,^19^,^20^, the attachment of rhizobia to roots^14^, the number of root nodules^15^, and the nitrogenase activity^18^.

*Sinorhizobium meliloti* and *Sinorhizobium medicae* are rhizobia with the ability to establish symbiosis with legumes of the genera *Medicago*, *Melilotus*, and *Trigonella*. These rhizobia are particularly sensitive to low pH^21^, growing either very slowly or not at all at pHs between 6.0–5.5 depending on the medium and the cultivation condition^12^,^22^,^23^. For this reason, acid tolerance has been considered a positive trait to perform well—in both survival and symbiosis—under acid conditions^24^. The screening for acid-tolerant alfalfa-nodulating isolates that could colonize and/or persist in acidic media and/or soils thus resulted in a selection of novel strains with enhanced saprophytic competence^11^ and/or improved symbiosis under moderately-acid conditions^23^,^25^,^26^,^27^,^28^,^29^. On the basis of these and other previous results, the genetic analysis of acid tolerance in rhizobia in general, and in alfalfa symbionts in particular, emerged as a potential powerful approach for a rational improvement of the rhizobial performance at low pH. As a result of the screening of Tn*5*-mutant libraries, several genes for acid tolerance (namely *act*, among others) could be identified in *S. medicae*—including *actA*^30^ and *actR/S*^31^,^32^, *actP*^33^, *exoH*^34^, and *exoR*^9^ along with some other genes that were induced at low pH, such as *phrR*^35^ and *lpiA*^36^,^37^. The collected genetic evidence indicates that tolerance to acidity in these rhizobia is a multigenic phenotype. Such a view is consistent with the failure to convert acid-sensitive rhizobia into acid-tolerant variants simply by the transfer of single cosmid clones generated from a more tolerant genotype (results from our laboratory). A more extensive understanding at the molecular level of the metabolism and responses of rhizobia to low pH remain essential for making an effective use of genetic manipulations in order to enhance bacterial acid tolerance.

Aiming at exploring other alternatives for improving acid tolerance in rhizobia, we attempted to induce transient phenotypes of acid tolerance. As previously demonstrated in *E. coli* and other bacteria^38^,^39^, an acid-tolerance response (ATR) could be induced in different rhizobia, including *S. medicae*^40^ and *S. meliloti*^41^ among others^42^. An increase in the tolerance to severe acid shocks was observed when rhizobia had been previously cultivated in batch under sublethal acidic conditions^41^,^42^. Tiwari *et al.*^31^ had demonstrated that the genes *actSR* were necessary for the induction of an ATR in *S. medicae*. Later on, Draghi *et al.*^41^ demonstrated that the ATR in *S. meliloti* was coupled to an increased symbiotic competitiveness for nodule occupancy, opening the possibility of exploring the stabilization of the ATR in rhizobia to be inoculated into acid soils. Unfortunately, no data were available on the molecular changes associated with that acid adaptation.

The subsequent approaches to improve the basic understanding of the rhizobial responses to extracellular acidity were based on different experimental configurations, all directed at characterizing the genetic expression and protein profile under either acid growth or acid shock. Transcriptional fusions generated by Tn*5* transposition demonstrated that acid-induced promoters were associated with cytochrome synthesis, potassium-ion cycling, and lipid biosynthesis and transport among other functions^43^. After performing a complementary study using proteomic tools, Reeve *et al.*^43^ suggested that the folding, proteolysis, and transport processes were key activities in *S. medicae* growing in acidity. While all these studies were performed on low-pH batch cultures, Hellweg *et al.*^44^ undertook an in-depth time-course analysis of the transcriptomic response in *S. meliloti* after shifting the bacterial cells from neutral to acid pH. The work revealed that a short-term exposure of the bacteria to low pH was sufficient to induce significant transcriptional changes in diverse rhizobial genes. All these studies provided useful—albeit fragmented—information on the phenotypes, and in certain examples, on the molecular components displayed by the rhizobia after challenge with a high concentration of extracellular hydrogen ions.

To date, most physiological and genetic studies on acid-stress in rhizobia have used batch cultures fraught with the consequent heterogeneity in cell physiology during cultivation, and also lacking an external control on the growth rate and other culture variables. The alternative use of continuous cultivation in a chemostat, already employed for the analysis of acid stress in rhizobia^45^, is an appropriate, and indeed preferable, choice for a more robust experimental design through the reduction of variables down to only the extracellular pH, thus leading to physiologically homogeneous bacterial populations and subsequent datasets^46^. Having used both classical and various omic tools, in this work we present a consolidated analysis of the responses of *S. meliloti* growing under a controlled acid stress. Changes in the transcriptome, proteome, and metabolome were integrated into a model to describe the cultural responses in the chemostat, together with the modifications in the enzymes and compounds of the central metabolic pathways.

## Results

### Cultivation parameters of *S. meliloti* 2011 growing in continuous culture in chemostat either at pH 7.0 or at nearly growth-limiting acidity

A continuous culture of *S. meliloti* 2011 was established at pH 7.0 in Evans minimal medium with glucose and ammonium as the respective carbon and nitrogen sources. The cells were cultured under the conditions indicated in Materials and Methods (*i*.*e*., D = 0.07.h^−1^, 28 °C, dissolved oxygen at saturation). Because of the C:N ratio in Evans defined medium the bacterial culture became N-limited at pH 7.0. Under steady-state conditions at pH 7.0 the culture reached a cell density of *ca*. 4.7 × 10^9^ c.f.u.ml^−1^ (OD^600nm^ = 4.6) at a biomass of 1.66 g.l^−1^ dry weight (Table 1). In order to determine the lowest extracellular pH at which *S. meliloti* was still able to grow in the chemostat, the pH of the extracellular medium was lowered stepwise at intervals of 0.2 pH units to achieve discrete steady states at each new pH (between pHs of 7.0 and 6.0). When the extracellular pH reached the value 6.0 (the acidic pH_limit_), the culture washed out, indicating that the rhizobia either stopped growing or duplicated at a rate that was lower than the one imposed by the chemostat-dilution rate. The continuous cultivation provided an optimal design for determining the lowest extracellular pH that was still compatible with the duplication rate imposed by the experimental setting. The acidic pH_limit_ in the chemostat proved to be comparable to those previously obtained in batch cultivation for *S. meliloti* and *S. medicae* (*i*.*e*., between 5.8–5.6)^23^,^47^. This information on the acidic pH_limit_ was used in a subsequent experiment to set up a continuous culture at pH 6.1 in order to have rhizobial cells growing just 0.1 pH unit above the condition where they had reached their limit of acid tolerance. Table 1 lists the cultivation parameters that characterized the growth of *S. meliloti* in the chemostat at pH 7.0 and at pH 6.1, including the data on the biomass production, number of culturable cells, and respiration indices. Under acidity a clear change in the limiting substrate occurred (an excess of near 7 mM ammonium was measured in the supernatant) accompanied by a 53% decrease in the biomass. The acid stress in the rhizobial cells at pH 6.1 was clearly reflected in that only 26% of the total bacteria counted in the Petroff-Hausser chamber were culturable (the number of living cells was estimated by plating). The more than 5-fold increase in the O_2_ consumption per living cell was observed in the culture at pH 6.1 compared to the same parameter in the culture at pH 7.0 indicated a higher aerobic respiration in the acid-stressed rhizobia. Finally, while cells produced significantly more EPS under acidity (Y_EPS/s_; *cf*. Table 1), no major change in the amount of polyhydroxybutyrate per cell was detected. The connection between EPS production and acid tolerance in rhizobia has been suspected since long. In *S. meliloti*, however, there is no evidence that EPS could have a positive effect on the bacterial acid tolerance. It has been previously reported that an *exoY S. meliloti* mutant, which lacks EPS, did not display any significant difference in its death rate when exposed at low pH^40^.

### Identification of protein markers induced during the continuous cultivation of *S. meliloti* 2011 at the acid pH 6.1 compared to neutrality

The cells collected from the continuous cultures were used to investigate the presence of molecular markers specifically associated with growth at either an acid pH or neutrality. In contrast to the limitations imposed by studies on batch cultures, bacterial cultivation in the chemostat at a constant dilution rate (D) provided a suitable experimental protocol for keeping rhizobia growing at the same population-doubling time, at an extracellular pH of either 6.1 or 7.0. The rhizobia in the culture at pH 6.1 continued to grow at 0.1 pH unit above their acidic pH_limit_ (*i*.*e*., pH 6.0, *cf*. the previous section).

**Figure 1:**
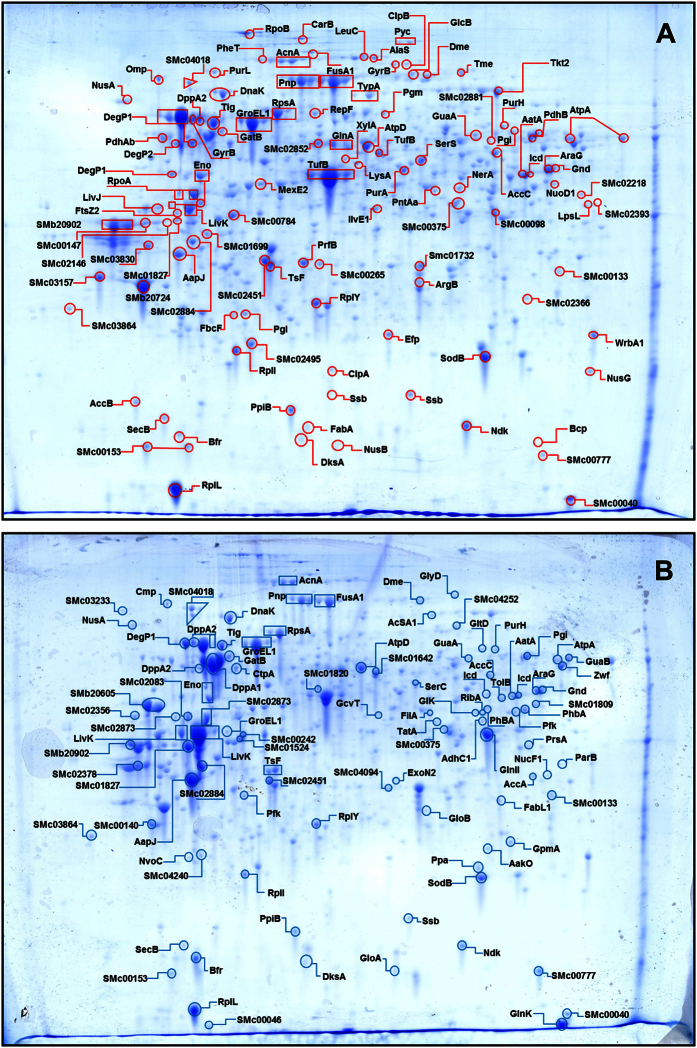
2-D–gel–electrophoretic profile of *S. meliloti* 2011 cytosolic proteins from cells grown in the chemostat at pH 6.1 and at pH 7.0. Isoelectric focussing of 100 μg of cytosolic proteins (first dimension, horizontal) was carried out on 24 cm IPG^TM^ (GE) strips with a final 4 to 7 pH gradient. The gel shown is representative of 4 independent technical replicates. The labels in the figure are the names of the polypeptides identified by UV-MALDI-TOF peptide-mass fingerprinting (Materials and Methods). Panel A Markers overexpressed at pH 6.1 compared to their relative expression at pH 7.0 (gel in B) were detected with the aid of the *ImageMaster* 2D^TM^ software (Fig. S2) and by visual inspection, and are all listed in Table 2. Panel B Markers overexpressed at pH 7.0 compared to their relative expression at pH 6.1 (gel in A) were also detected with the aid of the *ImageMaster* 2D^TM^ software (Fig. S3) and by visual inspection, and are all listed in Table 3.

The cytoplasmic proteomes from cells harvested from each chemostat culture were isolated as described in Materials and Methods and analyzed by 2-D–gel electrophoresis. Figure 1A,B show representative gels corresponding to the rhizobia grown at pH 6.1 and at 7.0, respectively. The identification of polypeptides was achieved by UV-MALDI-TOF™ peptide-mass fingerprinting (*cf*. Materials and Methods). The gel position of most identified products corresponded to the expected molecular mass and isoelectric point of each protein species as inferred from the *S. meliloti* genomic sequence (Fig. S1). With the aid of the *ImageMaster *2D^TM^ software we automatically recognized 382 protein spots (the red-outlined spots in Figs S2 and S3), with those representing more than 6% of the total translation products predicted from the genomic sequence^48^. Such a proportion is consistent with the 13% previously reported by Djordjevic *et al.*^49^ when they analyzed the *S. meliloti* proteome under five different physiologic conditions. In some instances, different molecular forms of a same protein were found at slightly discrepant positions in the gel, an observation that was more frequent in the proteome from rhizobia grown at pH 6.1 (i.e. five molecular forms for Pnp, three for DegP1 and FusA, and two for GyrB and Tig; Fig. 1A) than in the rhizobia grown at pH 7.0 (i.e. four molecular forms for DppA2 and two for LivK; Fig. 1B).

After a comparative analysis of the 2-D gels, both by visual inspection and by using the *ImageMaster* 2D^TM^ software, we detected 43 and 28 protein markers specifically overexpressed under the conditions of acid-stress and neutrality, respectively (*cf*. the list of differentially expressed markers at pH 6.1 and pH 7.0 in Tables 2 and 3, respectively). Two types of such markers were defined: *class-I*, corresponding to polypeptides detected under only one of the two conditions analyzed (*i*.*e*., either in cells from the acidic or the neutral culture: that is, markers with an *on-off* expression pattern); and *class-II*, corresponding to polypeptides present under both conditions but expressed at different relative intensities. Out of the 71 differentially induced polypeptides, only 9 were *class-I* markers (*on-off*). Of these 9, 8 were found in the rhizobia grown at pH 7.0 (SMc00242, SMc01827, IlvE1, AglE, ExoN2, SMa1507, SMa2361, and SMb20605), and only one was found in the rhizobia grown at pH 6.1 (GyrB). Most of the 62 *class-II* markers corresponded to proteins overexpressed at pH 6.1, thus revealing a higher set of induced proteins under the acid-stress conditions.

**Figure 2:**
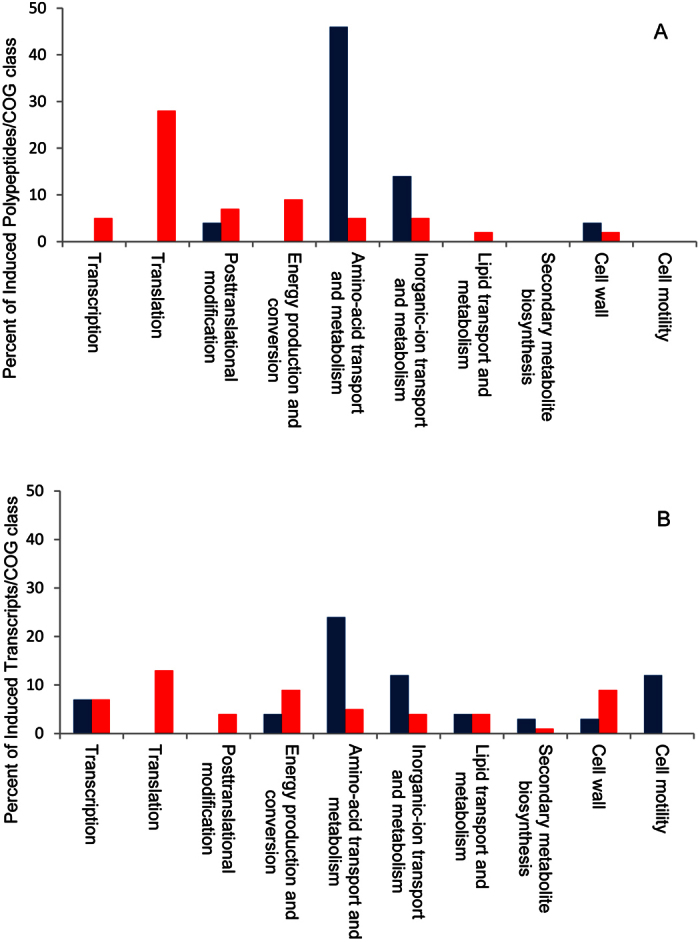
Incidence of the main COGs among the differentially expressed markers at pH 6.1 and 7.0 as revealed by the proteomic (**A**) and transcriptomic (**B**) analyses. The abundance of the COG classes indicated on the abscissa is expressed as a percent (ordinate). pH 7.0: blue ; pH 6.1: red.

Furthermore, clear differences were evident in the predicted functions associated with the induced markers under each pH condition (*cf*. Tables 2 and 3, last two columns). At neutral pH most overexpressed polypeptides (75% comprising *class-I* and *-II* markers) corresponded to genes belonging to clusters of orthologous groups (COGs) of transport and metabolism (Fig. 2, Panel A, blue bars). In the rhizobia growing at extracellular pH 6.1 most induced polypeptides corresponded to genes pertaining to COGs related to translation (28%), post-translational modifications (7%), and energy production and conversion (9%; Fig. 2, Panel A, red bars), thus indicating a change in the functional priorities under acid stress. While at pH 7.0 the transport and biosynthesis of cellular compounds emerge as quite active processes, under acid stress most of the overexpressed markers were associated with protein biosynthesis and energy metabolism. In the rhizobia growing at pH 6.1 the ribosomal polypeptides L25 (RplY) and L9 (RplI) were increased as well as the ATP-dependent chaperone Tig, a peptidylprolyl-*cys-trans* isomerase which interacts with short nascent polypeptide chains^50^. Likewise, related to translation at pH 6.1, a significant increase in the release factor PrfB and in the elongation factors EF-TU (TufB), EF-P (Efp), and EF-G (FusA) occurred; which proteins participate in the binding of aminoacyl-tRNAs to the ribosome and in the translocation of growing peptidyl-tRNAs^51^,^52^,^53^,^54^. These results, likely related to different needs for protein synthesis in the acid-stressed rhizobia, are consistent with recent publications where specific changes in ribosomal-protein composition were also observed in the responses of bacteria^55^ as well as in different eucaryotes^56^,^57^ to stress. In our experiments, the observed changes appeared to be restricted to a set of translation-related proteins with no significant modifications in the cellular amounts of the 16S and 5S rRNAs (Fig. S4). All these results point to the higher demands on protein synthesis in the acid-stressed rhizobia that grow in the chemostat at the same population-doubling time as those at pH 7.0. The parallel increase in the protease DegP^58^ also suggests a higher rate of protein degradation and turnover under conditions of acidity. This protease had been previously reported to be necessary for the normal growth of *S. meliloti* under high-temperature stress^59^, and might likely be the consequence of an increased protein damage. Consistent with such a scenario, an overexpression of the Pnp ribonuclease^60^ was also observed at pH 6.1, likewise suggesting a parallel increase in RNA degradation.

An analysis of Tables 2 and 3 also indicated that rhizobia in the acidic chemostat culture induced membrane-associated proteins such as the translation products of SMc03157 (a lipoprotein with homologs related to transport processes^61^), SMc01845 (a peptidoglycan-binding protein related to certain cell-wall–degradation enzymes^62^,^63^), and the response regulator ChvI, which protein interacts with the membrane histidine-kinase receptor ExoS^64^. ChvG/ChvI, the *Agrobacterium tumefaciens* homolog of ExoS/ChvI, was found to be involved in the tolerance to acid stress^65^. In *S. meliloti*, this two-component system has been shown to be physically associated with the periplasmic ExoR as well as being involved in EPS biosynthesis and symbiosis^66^, with several molecular targets of this regulon having been recently identified^67^.

### Transcriptomic analysis of *S. meliloti* growing in the chemostat at pH 6.1 compared to that of rhizobia growing at neutral pH

With the aim at exploring the profiles of gene expression in acid-stressed and control rhizobia, we performed a global transcriptional analysis in microarrays. The relative gene expression (M value) could be estimated for 3,750 transcripts (p < 0.05), representing about 60% of the total number of genes. Of these loci, a higher proportion of those being induced occurred under acid stress. For example, we observed 393 transcripts induced at pH 6.1 (M ≥ 1) compared to 225 transcripts induced at pH 7.0 (see Table S1). The analysis of COGs in the induced transcripts was in striking parallel with the results obtained from the rhizobial proteomes (Fig. 2, Panel B compared to Fig. 2, Panel A), except for membrane associated markers. Several induced markers were associated with translation and amino-acid transport plus metabolism at pH 6.1 and 7.0, respectively. When the transcriptomic data became available for the differentially induced polypeptides listed in Tables 2 and 3 (columns 7 and 8), concordant results were observed with positive M values for the polypeptides overexpressed under acidity and with negative M values resulting for polypeptides overexpressed at neutral pH.

The transcriptomic analysis was particularly useful for investigating gene expression in envelope-associated markers that were excluded from the present cytosol-proteome analysis. Thus, genes of the motility apparatus and the chemotactic machine were observed to be repressed under the condition of acidity. The repressed genes included *flaA*, *flaB*, *flaD* (flagelins); *flgB*, *flgF*, *flgG* (flagellar basal-body rod proteins); *flgL* (flagellar hook-associated protein); *fliE* (flagellar-hook basal-body protein); *fliM* (flagellar motor-switch transmembrane protein); and *cheE*, *motB*, *a*nd *motD* (chemotaxis proteins). Conversely, at pH 6.1 we observed an induction of the genes *murE* (encoding an UDP-N-acetylmuramoylalanyl-D-glutamate-2,6-diaminopimelate ligase; M = 1.9) and *murI* (encoding a glutamate racemase; M = 2.1); which enzymes participate in peptidoglycan biosynthesis, an observation that suggests an intermembrane-space remodelling under acid stress. Likewise, the accompanying induction of genes such as *accA* (acetyl-CoA carboxylase carboxyltransferase subunit alpha; M = 2.3) and *plsX* (acyl-acyl carrier protein phosphate acyltransferase; M = 1.6)—those enzymes related to fatty-acid and phospholipid biosynthesis—points to modifications in the lipid composition of the membrane under acidity. Changes in membrane components related to energy metabolism were also evident from these transcriptome data, particularly with respect to the genes encoding electron transporters such as *cycM* (cytochrome c; SMc02897; M = 2.6), *cycB2* (SMa1170; M = 2.0), *azu1* (SMa1243; M = 2.3), *fixN1* (SMa1220; M = 2.1), and *fixG* (SMa1211; M = 2.2), all of which *loci* increase their transcription at pH 6.1. The parallel increase in the transcriptional activity of genes encoding subunits of the membrane ATPase (*atpA*, the gene of the subunit alpha of the F0F1 ATP synthase; M = 1.6) and the NADH dehydrogenase (*nuoA1*, *nuoB1*, *nuoC1*, and *nuoE1*; genes encoding the subunits A, B, C, and E of the enzyme; M values from 2.5 to 1.4) strongly indicates the occurrence of a higher energy demand, electron-transport rate, and degree of oxygen consumption (Table 1) under acid stress.

Another key group of markers that frequently escape in-gel proteome analyses are those related to nucleic-acid metabolism and transcriptional regulation, owing to either their usually high isoelectric-point values—many are basic proteins—or their low cellular concentrations. In rhizobia growing at pH 6.1 we detected a higher expression of genes that encode transcription factors, and members of two-component systems such as: SMa0849 (*syrM*), SMc00929 (putative homolog of *gcvA*), SMa0760 (a gene encoding the antikinase FixT2), SMa1179 (*nosR*), SMc00458 (*feuP*, encoding a response regulator), SMa0815 (*nifA*), SMc02366 (*ragA*, encoding a response regulator), SMb20683 (encoding a transcriptional regulator of the LysR family), SMc00681 (*lrp*), SMc02560 (*chvI*, encoding a response regulator also detected in the proteome), SMc00109 (gene for a probable transcriptional regulator), SMa1705 (encoding a transcriptional regulator of the MucR family), and SMc01507 (encoding a sensor histidine kinase^68^). Of these genes, the *S. meliloti feuP* has been previously shown to be required for the expression of a *wild-type* hypoosmosis-tolerant phenotype through correct exportation of cyclic β-glucans^69^. These results therefore indicate that low pH induces central regulators which change the saccharidic composition of the bacterial envelope. Since in other bacteria *gcvA* —the SMc00929 homolog— and *chvI* have both been associated with responses to acid stress^70^,^71^, these genes likely participate in an evolutionarily conserved response mechanism.

Finally, the transcriptome made evident as well the induction of several genes associated with the increase in general transcriptional and translational activities, supporting the results discussed in the previous sections. Accordingly, at low pH higher transcriptional activities of genes encoding ribosomal proteins, subunits of the RNA polymerase [SMc01317 (*rpoB*, gene for the DNA-directed–RNA-polymerase beta chain), SMc02408 (*rnpO*, gene for the DNA-directed–RNA-polymerase omega subunit)], and the Rho termination factor [SMc02796 (*rho*)] have been detected. An increase in RNA metabolism may likewise be inferred from the enhanced transcription of the ribonuclease genes SMc01365 (*rnr*, a probable exoribonuclease II) and SMc02652 (*rnc*, a probable ribonuclease III) and from the increase in different RNA helicases [*e*.*g*., SMc00522 (*rhlE*) and SMc03877 (*mgpS*)], which enzymes in prokaryotes have already been associated with housekeeping functions and with structural adaptations of RNAs and ribosomes to abiotic stress^72^.

### Genome distribution of acid-induced markers

The proteome and transcriptome data were used to map, within the *S. meliloti* genome, the position of the markers with increased expression under the acidic condition (*cf*. Fig. S5). Consistent with the observation that many acid-induced markers are involved in central metabolic activities (Tables 1 and 2, and Supplementary Table S1), the *S. meliloti* chromosome proved to be the replicon with the highest density of acid-induced genes (on the average, *ca*. 8 genes with M ≥ 1 per 100 kb). With respect to the two symbiotic megaplasmids, pSymA encoded an average of 5 acid-induced genes per 100 kb, compared to the *ca*. 3 per 100 kb present in pSymB. Within this general context, the presence of a region of near 120 kb on the pSymA where we observed the striking value of *ca*. 21 acid-induced markers per 100 kb (indicated with asterisk on the pSymA in Fig. S5) is remarkable. The higher GC content within that region compared to that of the complete pSymA (61.36% vs. 60.4%) suggests that several of these genes might be part of a horizontally acquired region within the pSymA DNA. This region includes ORFs that codify for a transcriptional regulator (SMa1207), a cation transporter (SMa1087), a DegP-like protease (SMa1128), two Fix (SMa1216 and SMa1220, both encoding cytochrome-*c* subunits) and two non-Fix (SMa1170, SMa1243) electron transporters, proteins related to nitrous- and nitric-oxid metabolism (SMa1279, NorE, SMa1179, NosR), and many acid-induced hypothetical proteins among other markers.

### Metabolome analysis of *S. meliloti* 2011 grown under either acidic or neutral extracellular conditions

**Figure 3:**
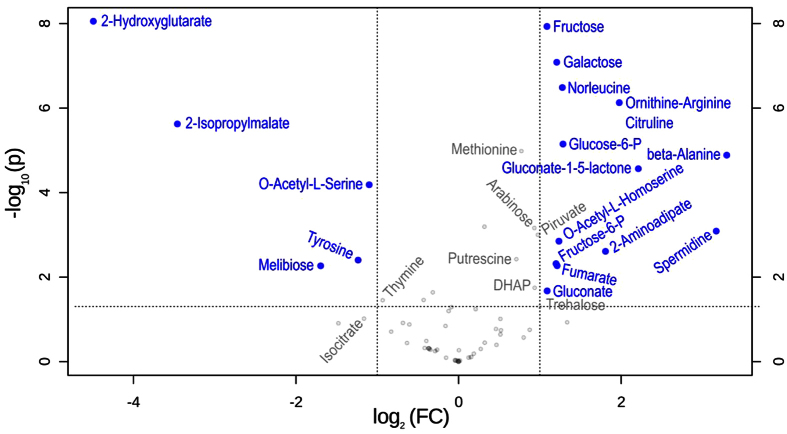
Volcano plot showing metabolites with fold changes (FC) ≥ 2 or ≤0.5 (p ≤ 0.05), when the pH of the extracellular culture was modified from 7.0 to 6.1 (FC = amount at pH 6.1/amount at pH 7.0). The plot shows the −log_2_ of the cytosolic amount of each metabolite at pH 6.1 with respect to the amount of the same species at pH 7.0. The signals for each metabolite were normalized to a constant cell dry weight and to the ribitol added as an internal standard (*cf*. Materials and Methods and Supplementary Table 2). The blue circles and names in blue indicate features (*i*.*e*., metabolites) with FC either ≥2 or ≤0.5 at a p ≤ 0.05. Calculations were performed by means of the MetaboAnalyst software. The data presented correspond to the statistical analysis of 4 technical replicates for each pH condition.

In order to explore the metabolites that characterize the growth of *S. meliloti* under different pH conditions, we performed the metabolome analysis described in Materials and Methods. The results identified and quantified 66 different compounds (*cf*. the data in Supplementary Table S2), whose relative amounts per cell dry weight enabled the analysis as to whether or not rhizobia have specific metabolite-compositional patterns associated with their extracellular pH of growth. The MetaboAnalyst software revealed several compounds whose intracellular concentrations significantly changed when the extracellular pH was modified. Figure 3 shows a volcano plot where the blue dots correspond to those compounds with a fold change (FC) higher than 2 (increased at pH 6.1) or lower than 0.5 (increased at pH 7.0), and a *t*-test threshold with a *p*-value lower than 0.05 (see the numerical data in Supplementary Table S3). In rhizobia grown under the indicated acidic and neutral pH, 13 and 6 compounds, respectively, became increased. Of these metabolites, glucose-6-P, gluconate-1,5-lactone, fructose, galactose, and phosphoenolpyruvate are all members of the central carbon pathways in *S. meliloti*^73^.

**Figure 4:**
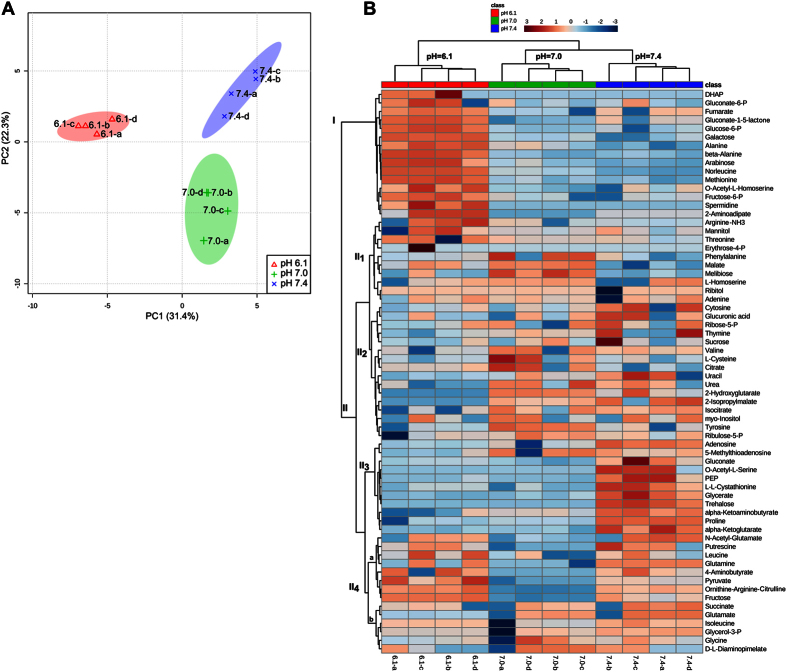
Multivariate chemometric and hierarchical-cluster analyses of the metabolome data from *S. meliloti* 2011 grown at pH 6.1, 7.0, and 7.4. (**A**) PCA showing the metabolite distribution within the PC1 *vs*. PC2 space of variation. The PCA analysis was performed on the cytosolic concentration of each metabolite normalized to a constant cell dry weight and to the signal of a known amount of added ribitol as an internal standard (*cf*. Materials and Methods plus the data in Supplementary Table 2). The PC1 space (x-axis) served to clearly separate samples from the rhizobia grown under acidity (pH 6.1) from the corresponding ones from the rhizobia grown at pH 7.0 and 7.4, which samples could otherwise be discriminated through the PC2 space (y-axis). The colored areas surrounding the data points represent 95% confidence limits. (**B**) Cluster display of metabolome data from rhizobia grown at different pHs. Each metabolite is represented by a single row of colored boxes, while each rhizobial sample from cells grown at the indicated pH (with 4 technical replicates each) is represented by a single column. The dendrograms and color images were produced with the MetaboAnalyst software. The color scale indicates the fold change (FC) with respect to the average value over the three pH conditions for each specific metabolite (red: values higher than the average; blue: values lower than the average).

In order to have an unsupervised method directed at finding metabolome changes associated with pH variations, we performed a Principal-Component Analysis (PCA) using the compositional data obtained for the rhizobia grown under each pH condition. The results demonstrated that the data from rhizobia cultivated at pH 7.0 and 6.1 mapped at different regions in the two-dimensional space of components 1 and 2 (PC1 *vs*. PC2; *cf*. Fig. 4, Panel A and the vector-correlation plot in Supplementary Fig. S6). That the metabolome data from cells grown in an independent chemostat at pH 7.4 (which we used as a control sample of rhizobia grown under a slightly basic pH) could also be differentiated from the other two sets of data is indeed interesting, and shows that an extracellular pH difference as low as 0.4 units—and in the alkaline direction—is sufficient to make the metabolic profiles in *S. meliloti* distinctly different. The cluster analysis presented in Fig. 4, Panel B identified and quantified changes in which specific sets of metabolites (*i*.*e*., profiles, metabolic patterns) became positively or negatively correlated with particular values of the extracellular pH. Whereas the cytosolic concentrations of the metabolites from cluster I were positively correlated with the rhizobial growth in the acidic medium at pH 6.1 (with the several having FCs ≥ 2 also being shown in the volcano plot in Fig. 3), the levels of the metabolites from cluster II_3_ became predominantly increased only at pH 7.4 (*i*.*e*., O-acetyl-L-serine, phosphoenolpyruvate, D, L-cystathionine, glycerate, threalose, proline, alpha-ketoglutarate, and alpha-ketoaminobutyrate), thus providing a set of marker compounds for rhizobial growth in a more alkaline medium. Other metabolites, such as those from cluster II_4a_ (*i*.*e*., N-acetylglutamate; glutamine; 4-aminobutyrate; ornithine, arginine, and citrulline; along with fructose, among others) increased at both pH 6.1 and pH 7.4 and thus constitute compounds marking culture-pH deviations from neutrality.

## Discussion

Increased hydrogen-ion concentrations in the environment have been demonstrated to strongly affect viability and symbiosis in *S. meliloti*^13^, the related species *S. medicae*^9^, and several other rhizobia^8^. In view of these findings and on the worldwide distribution of acidity in agricultural soils, an understanding of the responses of rhizobia to low pH becomes a central issue in the attempt to improve symbiosis in moderately acidic soils. In the present work we investigated the physiologic and molecular responses to extracellular acidity of the model rhizobium *S. meliloti*. In order to characterize the bacterial changes induced by low pH, we set up *S. meliloti* steady-state cultures in a chemostat, an experimental system allowing a strict control of cultivation parameters. The use of a chemostat also enables, at the same time, the acquisition of reliable and homogeneous datasets, obviating the secondary growth effects intrinsically associated with the more heterogeneous batch cultures^46^. The results of the experiments summarized in Table 1 indicated a limiting growth in acidic medium close to pH 6.0–6.1 for *S. meliloti* 2011; a condition where we observed a significant proportion (*ca*. 74%) of bacteria that were unable to grow (results from the plating experiments), in agreement with the acidic stress imposed. Within such a context, the parallel increase in oxygen consumption by metabolizing bacterial cells is reflected in a higher energy demand for both repair and biosynthesis under conditions of acidity (*i*.*e*., for both maintenance and growth). The observed increase in several of the ribosome-associated proteins (*e*.*g*., RplI, RplY, RplL, RpsA, TufB, PrfB, and Efp; *cf*. Table 2) provided parallel evidence in support of a higher demand of protein synthesis during the growth at lower pH. Most notable is the difference between the two systems of cultivation in that, whereas in order to alleviate an acid stress, in batch cultures rhizobia decrease their duplication rate^42^; those same bacteria when confronted with an equally low pH in the chemostat are forced to maintain their replication (at a constant dilution rate) at the same rate as that used at pH 7.0.

As demonstrated here, the adaptive process included changes in transcripts associated with membrane components, such as the biosynthetic enzymes of phosphatidylglycerol derivatives (Smc00611, a homolog of *lpiA* from *R. tropici*^36^) and ornithine-containing lipids (Smc01116, namely *olsA* in *S. meliloti*^74^: note, too, that we also observed an increase in the cytosolic concentration of the ornithine substrate under acidity). Both of these lipids were relevant for acid tolerance in *R. tropici* through different proposed mechanisms (for a recent review see ref. 75). The correlation between the observations in *R. tropici* and *S. meliloti* suggests that membrane-lipid remodelling is a general phenomenon associated with acid adaptation in rhizobia, if not in other bacteria as well.

The remarkable increase in aerobic respiration (more than fivefold) under acid stress quantitatively reflects the magnitude of the impact of protons on the rhizobial energy demands. The omic analyses demonstrated an enhanced transcription of chromosomal genes encoding different cytochromes, part of the respiratory ATPase, and several subunits of the membrane-associated NADH dehydrogenase. A significant increase in the transcription of the pSymA-encoded cytochrome genes *cycB2*, *fixN1*, and *fixG* was likewise observed under acidity; in agreement with the previous evidence on the role of pSymA in stress responses^76^. The increased transcription of *fixN1* in particular had also been reported in a strain of *S. medicae* incubated in batch at low pH^77^. The present results would indicate that the cellular role of certain of the *fix* genes exceeds their sole participation in nitrogen fixation during late symbiosis. The observed changes in several cytochrome-associated transcripts could be related to a reinforcement and/or switching-on of alternative destinations for the reducing power generated under acid stress. Other authors have suggested that alternative respiratory pathways may be associated with changes in the P/O ratios under specific circumstances, thus making the system more flexible to respond to environmental variations^78^.

**Figure 5:**
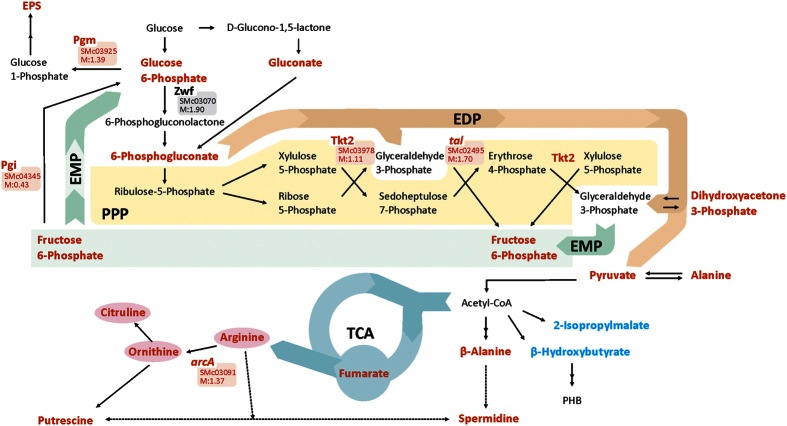
Graphical representation of *S. meliloti* central carbon-metabolic pathways where significant changes in metabolites, transcripts, and/or specific enzymes were observed when rhizobia were grown under acid stress. Metabolites in red correspond to those with an increased concentration at pH 6.1 relative to neutrality according to the analysis shown in the volcano plot from Fig. 3 and/or in the cluster from Fig. 4, Panel B. The transcripts and enzymes in red color correspond to induced markers from the differential transcriptome and proteome shown in Supplementary Table 1 and Table 1, respectively. PPP: pentose-phosphate pathway, EDP: Entner-Doudoroff pathway, EMP: Embden-Meyerhof-Parnas pathway, TCA: tricarboxylic-acid cycle, *arcA* (Smc03091): arginine deaminase, *zwf* (Smc03070): glucose-6-phosphate dehydrogenase, *tal* (Smc02495): transaldolase, Tkt2 (Smc03978): transketolase, DHAP: dihydroxyacetone phosphate, PHB: polyhydroxybutyrate, EPS: exopolysaccharide, Pgi: phosphoglucose isomerase, Pgm: phosphoglucomutase.

In view of the known active pathways in *S. meliloti*^73^, we could infer that energy and reducing power for biosynthesis should derive from the Entner-Doudoroff pathway and from the pentose-phosphate pathway (PPP). According to our results, the higher expression under acidity (of the mRNAs and proteins) of glucose-6-phosphate dehydrogenase (*zwf*) together with the transketolase (Tkt2) and the transaldolase (*tal*) is strongly suggestive of an increased activity in both the oxidative and the nonoxidative branch of the PPP. The observed induction under acidity of phosphoglucose isomerase (Pgi), the first enzyme of the incomplete Embden-Meyerhof-Parnas pathway in *S. meliloti*, suggests an increased recycling of fructose-6-P synthesized in the nonoxidative branch of the PPP back into glucose-6-phosphate^79^. In agreement with this possibility, both the substrate fructose-6-P and the product glucose-6-P were found in higher concentrations in the acid-stressed rhizobia. Furthermore, the increased concentration of glucose-6-P and phosphoglucomutase are both consistent with the increased EPS biosynthesis also observed under acidity. The induction of the EPS-biosynthetic genes following a short exposure of rhizobia to low pH has already been reported^44^. In addition to these considerations and the complex hierarchical regulation of EPS biosynthesis in *S. meliloti*^80^, the increased production of EPS in the acidic continuous culture may also have been evoked by a metabolic overflow of carbon *via* the oxidative PPP, derived from higher demands for the NADPH used for biosynthesis and repair (*cf*. the schematic summary of these metabolic interactions in Fig. 5). The concept of metabolic overflow was originally introduced in microorganisms to describe the excretions of specific by-products as a consequence of their growth under glucose excess^81^,^82^,^83^,^84^,^85^. The increased EPS production by rhizobia under acid stress resembles microorganism responses during metabolic overflow, where cells dispose of different compound(s) —those derived from central metabolic intermediates— in order to keep specific metabolic pathways active. Nonetheless, cellular needs that impel “classic” metabolic overflows are clearly different from those that operate under the acid stress studied here; where, in contrast to a repression in the respiratory chain, a strong increase in aerobic metabolism occurs. Unfortunately, we have no data on the exometabolome of the rhizobia in the chemostat in order to evaluate the presence of by-products that could be derived from the observed increase in the cytosolic amounts of three-carbon central metabolites (*e*.*g*., dihydroxyacetone-3-phosphate and pyruvate, Supplementary Table 3, Fig. 5).

Acid- and neutrality-grown rhizobia, though clearly recognizable through the differential-expression patterns observed in specific RNAs and proteins, could also be unequivocally distinguished through their cytosolic metabolomes. The possibility of associating metabolomes with extracellular pHs does not appear to be restricted to a comparison of widely different conditions (*i*.*e*., neutrality *versus* a growth-limiting acidity); and especially not so, since we could also clearly differentiate the metabolomes from cells grown at pHs 7.0 and 7.4 (Fig. 4). Thus, we observed a quite strong dependence of the metabolome of the rhizobia on (even limited) changes in the extracellular pH (<0.5 units).

The results presented in this report have demonstrated that *S. meliloti* has a complex process of adaptation to acidity in agreement with previous findings in support of the multigenic character of the response. The acidic condition of growth modified transcription (|M| ≥ 1, p ≤ 0.05) in nearly 10% of the genome (Supplementary Table 1). We have demonstrated here that the genetic information associated with the growth of *S. meliloti* under acidity is spread throughout the genome. The chromosomal location of most of the acid-regulated genes indicates that adaptation to acidity requires the readjustment of diverse central functions (*e*.*g*., the expression of membrane proteins related to respiration and lipid metabolism and changes in the central carbon metabolism). Besides such general mechanisms of adaptation—those likely existing since the presymbiotic state within the rhizobial evolution—an intriguing expression of specific *fix* and other pSym-encoded markers was also observed.

The induction of pSym markers by acid may be related to recent results revealing the presence of acidic environments along the infection pathway. Novel data obtained through the use of fluorescent probes demonstrated the existence of an acidic environment at the “bright spot” during the initiation of root-hair infections in *M. sativa*^86^ as well as later on during symbiosis in the peribacteroid space within zone III of N_2_-fixing nodules^87^. That some acid-responsive pSym markers detected in the acidic culture could also be induced *in planta* as a primary response against the reported extracellular low pH is thus conceivable. Recent transcriptome data from laser-dissected nodules showed that several pSymA genes induced within infected nodules were also overexpressed under acidic cultivation, thus confirming the versatile role of these pSym replicon^88^.

All this evidence taken together has indicated that the growth of *S. meliloti* under stressing acidic conditions in the chemostat resulted in clear biochemical modifications; whose main features are summarized in the scheme depicted in Fig. 5. The differential proteome and transcriptome revealed changes in proteins and genes associated with the bacterial surface (*i*.*e*., in lipid metabolism involving cell envelope), alterations in the central carbon metabolism and in respiration, and modifications in the amount of EPS secreted, among others. A new specific metabolome profile could be established, with changes in many compounds that correlated with the observed shifts in the proteome and transcriptome (*i*.*e*., intermediates of the PPP pathway). The quantitative rhizobial metabolome emerged as a substantially sensitive indicator of (even subtle) changes in the extracellular pH.

The question thus remains as to what could now constitute promising avenues in order to gain an understanding and improvement of the rhizobial behavior and symbiosis under moderate acidity. Unfortunately, we do not understand the relevance to acid tolerance of most of the differentially expressed markers identified in this present investigation. Thus, the phenotypic impact of specific mutants should now be investigated. Further analyses should also be focussed on the identification of the initial acid-induced molecular events that trigger the downstream biochemical changes described here. Additional approaches to improving the response of *S. meliloti* to low pH could also be addressed by the identification of the genes and processes that are part of the ATR, through which the bacteria transiently acquire both a higher tolerance to acidity, and an improved symbiotic competitiveness^41^. We need to learn, for example, how the molecular changes that take place during the ATR compare with the responses observed here. The connection between the transient tolerance to acidity and the concomitant improvement in symbiosis make the process of acid adaptation a suitable target for upcoming research towards an eventual use of the adapted phenotype in preconditioning rhizobia of inoculants.

## Materials and Methods

### Bacterial strains and growth conditions

*Sinorhizobium meliloti* Rm 2011, whose fully genomic sequence is available, was used in this work (GenBank accession numbers: NC_020528.1, NC_020527.1, and NC_020560.1). For starter batch cultures and nutrient-limited continuous cultures in chemostat Evans defined minimal medium was used^89^, containing glucose (10 g.l^−1^) and ammonium chloride (0.7 g.l^−1^) as the respective carbon and nitrogen sources. The use of glucose, which is metabolized by *S. meliloti*, facilitated the calculation of basic cultural parameters in the chemostat such as the yields Y_X/S_, Y_CO2/S_, and Y_EPS/S_ presented in Table 1.

### Continuous-culture setup

Continuous cultures of *S. meliloti* were performed at 28 °C in a 2-L Bioflo IIe (New Brunswick Scientific Co., Edison, NJ, USA) reactor with a working volume of 1.5 l. The growth rate (*i*.*e*., dilution rate) was adjusted at 0.07 ± 0.01 h^−1^. The cultures were flushed with filtered sterile air (20 l.h^−1^) at flow rates measured with a bubblemeter. The dissolved-oxygen concentration was continuously measured with an Ingold (Wilmington, MA, USA) polarographic probe. Oxygen and CO_2_ concentrations were determined by a paramagnetic oxygen analyzer (Servomex 1100A; Norwood, MA, USA) and an infrared CO_2_ analyzer (Horiba PIR 2000; Japan), respectively. The rates of oxygen consumption and CO_2_ production were calculated by a mass-balance method according to Cooney^90^. The pH was automatically maintained at either 7.0 ± 0.05, 6.1 ± 0.05, or 7.4 ± 0.05 through the addition of 1 N NaOH. Glucose concentrations in the culture media and supernatants were determined with a glucose-oxidase enzymatic kit (Wiener Lab, Argentina) and ammonium concentrations in the media and supernatants were measured by the indophenol method^91^. The biomass dry weight and rates of oxygen consumption were calculated as previously described^92^. Bacterial cells were counted in a Petroff-Hausser chamber and the culturable cells determined by plating culture samples in agarized Evans medium. Cultures were considered to be under steady-state conditions when the biomass concentration and specific rate of oxygen consumption varied by less than 10% during three retention times.

### Exopolysaccharide (EPS) and polyhydroxybutyrate determination

To isolate the EPS, the supernatant from continuous cultures was precipitated with three volumes of cold ethanol and incubated at −20 °C overnight. The mixture was centrifuged at 10,000 × *g* for 15 min. The pellet was dissolved and dialyzed against distilled water, lyophilized, and stored at −80 °C until use. The amount of EPS in these samples was determined by the Anthrone Method as previously described^93^.

The polyhydroxybutyrate content in the cell pellets was determined as chrotonic acid in H_2_SO_4_, as described by Law *et al.*^94^.

### 2-D Gel electrophoresis

One liter of culture was harvested and centrifuged at 10,000 × *g* for 20 min at 4 °C. The pellets were washed twice with cold phosphate-buffered saline; centrifuged as above; and the final pellets frozen immediately in liquid nitrogen, lyophilized, and stored at −80 °C until use.

To obtain the protein extracts, 100 mg of dry cells were suspended in 5 ml of 10 mM Tris-HCl pH 7.6 with Complete Protease Inhibitor Cocktail™ tablets (Roche) and sonicated for 1 min in 5–10 sec cycles in an ice-water bath at 50% power with a Sonifier 150™ (Brandson Ultrasonic Corporation; Connecticut, USA). The samples were then centrifuged at 10,000 × *g*, for 20 minutes at 4 °C and the supernatants centrifuged at 100,000 × *g* for 1 h at 4 °C to eliminate cell debris. The supernatants were incubated with 0.5 μg.ml^**−**1^ Benzonase Nuclease™ (Sigma Aldrich; St. Louis MO, USA) for 30 min at 37 °C and the proteins finally precipitated with four volumes of cold acetone followed by an overnight incubation at −20 °C. After a 15-min centrifugation at 10,000 × *g* the pellets were washed twice with cold acetone. The air-dried pellets were finally suspended in 500 μl of rehydration buffer (RB) containing 8 M urea, 2% (w/v) 3-([3-chloramidopropyl]dimethyammonio)-1-propane-sulfonate (CHAPS), and 0.01% (w/v) bromophenol-blue. The total-protein contents were determined by the Bradford method (BioRad kit; Hercules, CA, USA).

### Isoelectrofocussing and two-dimensional sodium-dodecyl-sulfate–polyacrylamide-gel electrophoresis (2-D-SDS-PAGE) of protein samples

Five hundred μg of total proteins dissolved in 450 μl of RB were mixed with 5 μl of IPG™ buffer for a strip-immobilized pH gradient from pH 4–7 (GE; Pittsburg, USA), and after the addition of 5 μl of 1 M dithiothreitol the sample was incubated on a shaker at 25 °C for 15 min. Then 450 μl were loaded onto a 24 cm, pH 4–7 Immobiline DryStrip™ (GE) and the proteins electrofocussed on the strips in an IPGphor™ unit (GE) for 1 h at 0 volts, 12 h at 30 volts, 2 h at 60 volts, 1 h at 500 volts, 1 h at 1,000 volts; <15 h at 8,000 volts. Isoelectric focusing was terminated after 75,000 volts h. After the electrofocussing, the strips were equilibrated in 5 ml of a solution containing 6 M urea, 50 mM Tris (pH 8.8), 30% (v/v) glycerol, 2% (w/v) SDS, and 2% (w/v) dithiothreitol on a tilt table for 15 min (the disulfide-reducing step). The solution was discarded and 5 ml of a second solution added for 15 min containing 6 M urea, 30% (v/v) glycerol, 2% (w/v) SDS, 2.5% (w/v) iodoacetamide, and 0.01% (w/v) bromophenol blue (SH alkylation step). The SDS-PAGE was performed on an EttanDalt™ (GE) electrophoresis unit. The strips were placed in a 1.5-mm thick 12.5% (w/v) polyacrylamide gel sealed with 0.1% (w/v) agarose in SDS-electrophoresis buffer (25 mM Tris, 192 mM glycine, 1% [w/v] SDS) containing 0.01% (w/v) bromophenol blue. The electrophoresis was run for 30 min at 4 watts per gel followed by a further run at 20 watts per gel until the bromophenol blue band reached the bottom of the gel. The gels were stained overnight with a solution of Coomassie Brilliant Blue (CBB: 5% [v/v] methanol, 42.5% [v/v] ethanol, 10% [v/v] acetic acid, 0.2% [w/v] CBB G250, 0.05% [w/v] CBB R250) and destained in the solution 5% (v/v) methanol, 42.5% (v/v) ethanol, 10% (v/v) acetic acid for 1 h then further in 7% (v/v) acetic acid until the background was sufficiently reduced. The gels were finally documented on an Imagescanner (GE).

### Image Analysis

The gels were scanned through the use of a transmission Image Scanner (GE). Image analysis was performed with *Image Master* 2D^TM^ Platinum (version 6.0) (GE) for at least three gels for each pH condition. Spot intensities were quantified according to the volume percent; and specific spots classified as up or down regulated whenever the average volume for a given spot at one pH condition was at least 50% different from the corresponding volume of the homologous spot under another pH condition, and presented simultaneously a *p*-value lower than 0.05 in a *t*-test.

### In-gel tryptic digest of proteins and mass spectrometry

Protein spots were excised from the 2-D gels and placed into microtiter-plate wells that had been previously washed twice with a trifluoracetic acid: acetonitrile: water solution (0.1:60:40 [v/v]). The tryptic digestions were performed as described on the Keck home page (http://info.med.yale.edu/wmkeck/prochem/geldig3.htm). Samples containing digested proteins were mixed 1:1 with a solution of water:acetonitrile:trifluoracetic acid (67:33:0.1 [v/v]) saturated with α-cyano-4-hydroxycinnamic acid. The mass spectra were obtained on an Ultraflex MALDI-TOF-MS™ instrument (Brucker, Bremen). The identification of proteins from each peptide-mass fingerprint was carried out by means of the Mascot software (Matrix Sciences, London, UK) with the aid of a *S. meliloti* database. The following search parameters were set in the Mascot software: enzyme, trypsin; missed cleavages, 1; peptide tolerance, 150 ppm; MH+; and monoisotopic.

### Transcriptomic analysis in microarrays in search of *S. meliloti* genes differentially expressed under acid and/or neutral extracellular conditions

For the microarray slides, RNA was isolated from the bacterial cells grown in the continuous culture stabilized at pH 7.0 or pH 6.1. The rhizobial cells were disrupted mechanically as described previously^95^. Cy3- and Cy5-labelled cDNAs were prepared by the method of De Risi *et al.* from 10 μg of total RNA^96^. Three slide hybridizations were performed with the labelled cDNA synthesized from both of the RNA preparations (*i*.*e*., pH 7.0, pH 6.1, 3 replicates each, technical replicates). Hybridization, image acquisition, and data analysis were carried out as previously described^95^,^97^. The mean-signal and mean-local-background intensities were determined for each spot on the microarray images by means of the ImaGene 5.5 software for spot detection, image segmentation, and signal quantification (Biodiscovery Inc., Los Angeles, USA). M and A values for individual spots in the microarrays were calculated as previously described^95^. Here a normalization method was used based on a local regression that accounts for the intensity and spatial dependence on the dye biases^98^. Normalization and t-statistics were computed by means of the EMMA 1.1 microarray data-analysis software developed at the Bioinformatics Resource Facility, Center for Biotechnology, Bielefeld University (http://www.genetik.uni-bielefeld.de/EMMA/; ref. 99. Genes were considered differentially expressed if obtained for at least five of the nine replicate spots and if the confidence indicator (*p*) was ≤0.05 and the log_2_ of the expression ratio (M) ≥ 1 or ≤−1 (*i*.*e*., if at least a twofold difference was obtained between the two experimental conditions). The microarray results were verified for a specific acid-induced transcript (*degP1* - SMc02365, M^pH 6.1/pH 7.0^ = 1.98; Supplementary Fig. 6) by real-time quantitative reverse-transcription PCR using a KAPA SYBR FAST One-Step qRT-PCR kit (Kapa Biosystems Inc. Wilmington, MA, USA) according to the manufacturer’s instructions. The arrays data have been deposited in the NCBI Gene Expression Omnibus database^100^ and are accessible through GEO Series accession number GSE74449.

### Metabolite extraction and derivatization for the metabolome experiments

The extraction and sample derivatization were performed as described previously^101^. One ml of 80% (v/v) aqueous methanol containing 10 μM ribitol (internal standard) was added to 10–30 mg dry weight of *S. meliloti* cells in 1.5-ml screw-cap tubes with 0.5 g acid-washed glass beads (Sigma-Aldrich). Immediately after adding the methanol, the cells were disrupted three times in a FastPrep™ instrument (Qbiogene, Heidelberg, Germany) at 6.5 m/s for 45 s. For improved extraction, the mixtures were further incubated at 70 °C for 15 min at 1,400 rpm in a Thermomixer™ (Eppendorf, Hamburg, Germany). After centrifugation at 18,500 × *g* for 20 min at room temperature the clear supernatant was evaporated to dryness in a nitrogen stream. Methoximation of carbonyl moieties with 50 μl of a 20 mg.ml^−1^ solution of methoxylamine hydrochloride in pyridine was run at 37 °C for 90 min with constant stirring. Dissociable protons were protected with trimethylsilyl groups through reaction with 50 μl N-methyl-N-[trimethylsilyl]trifluoroacetamide at 37 °C for 30 min.

Gas chromatography–mass-spectrometry (GC-MS) analysis was performed with a TraceGC™ gas chromatograph and a PolarisQ™ ion-trap mass spectrometer equipped with an AS2000™ auto sampler (Thermo Finnigan, Dreieich, Germany). Sample volumes of 1 μl were injected at a 250 °C injector temperature without a split. The gas chromatograph contained a 30-m X 0.25-mm Equity-5™ column having a 0.25-μm 5% diphenyl- 95% dimethylsiloxane coating (Supelco, Bellfonte, Calif.). The interface temperature was adjusted to 250 °C and the ion source set at 200 °C. The helium carrier gas was set at a constant flow of 1 ml.min^−1^. After a 3 min constant heating at 80 °C the oven temperature was raised in steps of 3 °C.min^−1^ to 300 °C. In order to equilibrate the system for the next injection, the temperature was set at 80 °C for 5 min. Mass spectra were recorded at 2 scans.s^−1^ with a scanning range of 50–550 m/z. Evaluation of the chromatograms was performed with the Xcalibur 2.0 software (Thermo Finnigan). Metabolites were identified by comparison with the NIST 98 (NIST, Gaithersburg, MD) database and with purified standards. Automatic peak quantification of selected metabolites was implemented into the processing setup of the Xcalibur software. Virtual fragmentations were performed with the Mass Frontier 2.0 software (Thermo). The data were log-normalized and uni- and multivariate tests applied through the Metaboanalyst server^102^.

## Additional Information

**How to cite this article**: Draghi, W. O. *et al.* A consolidated analysis of the physiologic and molecular responses induced under acid stress in the legume-symbiont model-soil bacterium *Sinorhizobium meliloti.* *Sci. Rep.* **6**, 29278; doi: 10.1038/srep29278 (2016).

## Supplementary Material

Supplementary Dataset 1

Supplementary Dataset 2

## Figures and Tables

**Table 1:** Cultivation parameters of *S. meliloti* 2011 grown in the chemostat at extracellular pH 7.0 and pH 6.1.

Cultivation parameters	Chemostat pH 7.0	Chemostat pH 6.1
Dilution rate (h^−1^)	0.073 ± 0.002	0.070 ± 0.003
Optical Density^(600nm)^	4.57 ± 0.02	1.82 ± 0.12
Bacterial cell number (cells.ml^−1^)^a^	4.70 × 10^9^ ± 0.9 × 10^9^	1.79 × 10^9^ ± 1 × 10^9^
Culturable cells/total bacteria (%) (c.f.u. × 100 total bacteria^−1^)^b^	100%	26%
Dry weight (mg.ml^−1^)	1.66 ± 0.11	0.78 ± 0.05 (0.20 g of these being able to grow)^b^
Cells/Dry weight (cells.mg^−1^)	2.8 × 10^9^ ± 0.6 × 10^9^	2.3 × 10^9^ ± 1.3 × 10^9^
Dry weight/cell (pg.cell^−1^)	0.35 ± 0.02	0.43 ± 0.02
EPS production (mg.mg dry weight^−1^)	0.04 ± 0.22	0.24 ± 0.06
PHB content (% w.w^−1^)	6.8 ± 0.12	5.3 ± 0.04
Glucose consumption (g.l^−1^)	3.04 ± 0.15	1.46 ± 0.08
Ammonia (limiting nutrient at pH 7.0) (mM)	ND	6.9
rCO_2_ (mmol.l^−1^.h^−1^)	3.58 ± 0.07	1.66 ± 0.01
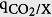 (mmol.C-mol^−1^ h^−1^)	56 ± 2	55 ± 2
CO_2_ production/culturable cells (fmol.cfu^−1^.h^−1^)	0.76 ± 0.01	3.56 ± 0.06 [↑ *ca*. 4.7 times]
rO_2_ (mmol.l^−1^.h^−1^)	3.97 ± 0.1	2.19 ± 0.1
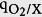 (mmol.C-mol^−1^ h^−1^)	62 ± 1	72 ± 1
O_2_ consumption/culturable cells (fmol.cfu^−1^.h^−1^)	0.8 ± 0.01	4.7 ± 0.2 [↑ *ca*. 5.8 times]
RQ (rCO_2_.rO_2_^−1^)	0.9 ± 0.04	0.76 ± 0.04
Y_X_/_S_ (C-mol.C-mol^−1^)	0.64 ± 0.04	0.62 ± 0.06
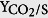 (mol.C-mol^−1^)	0.48 ± 0.01	0.49 ± 0.01
Y_EPS/S_ (Y_P/S estimation_) (C-mol.C-mol^−1^)	0.02 ± 0.01	0.13 ± 0.03
C balance ( 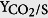 + Y_X/S_ + Y_EPS/S_)	1.14 ± 0.01	1.24 ± 0.1

All values are expressed with their corresponding standard deviations.

rO_2_: rate of O_2_ consumption (mmol.l^−1^.h^−1^).

q_O2_/_X_: mmol O_2_ consumption/C-mol of biomass/h.

rCO_2_: rate of CO_2_ production (mmol.l^−1^.h^−1^).

q_CO2_/_X_: mmol of CO_2_ production/C-mol of biomass/h.

Yx/s: biomass-growth yield (C-mol of biomass produced/C-mol of consumed glucose).

Y_CO2/S_: CO_2_ production (molCO_2_/C-mol of consumed glucose).

Y_EPS/S_: exopolysaccharide production (C-mol EPS/C-mol of consumed glucose).

RQ: Respiratory Quotient.

ND: Non-detected.

^a^Number of bacteria counted in Petroff-Hausser chamber.

^b^Proportion of bacteria counted by plating with respect to those counted in the Petroff-Hausser chamber (%).

**Table 2:** *S. meliloti* 2011 protein markers differentially overexpressed under continuous cultivation of the rhizobia at extracellular pH 6.1 compared with the expression of the same markers at pH 7.0.

Gel Code (a)	ORF	Protein name (a)	Accession number	Sequence coverage (%)	Score (MASCOT)	Ratio of intensities (pH 6.1/pH 7.0) (b)	M Value (h) (transcriptome)	Predicted/putative function	COG (i)
**1**	**SMc02365**	**DegP1 (c)**	**Q52894**	**41**	**154**	**4.80**	**1.97**	Probable serine protease do-like precursor (EC 3.4.21.-).	O
**2**	**SMc02365**	**DegP1 (c)**		**34**	**148**				
**3**	**SMc02365**	**DegP1 (c)**		**38**	**181**				
**4**	**SMc02050**	**Tig (d)**	**Q92Q12**	**46**	**181**	**1.49**	**1.55**	Trigger factor (TF).	O
**5**	**SMc02050**	**Tig (d)**		**47**	**193**				
**6**	**SMc02782**	**GyrB (e)**	**Q92TE4**	**24**	**136**	**ON**	**0.59**	DNA gyrase subunit B (EC 5.99.1.3).	L
**7**	**SMc02782**	**GyrB (e)**		**10**	**81**				
**8**	**SMc03157**	**–**	**Q92LX5**	**39**	**155**	**2.78**	**1.23**	Outer membrane lipoprotein 3 precursor (PLP3).	P
**9**	**SMb20724**	**–**	**Q92TR9**	**30**	**84**	**2.00**	**0.65**	Hypothetical protein.	S
**10**	**SMc01312**	**FusA (f)**	**Q92QH2**	**25**	**116**	**1.43**	**0.61**	Elongation factor G (EF-G).	J
**11**	**SMc01312**	**FusA (f)**		**41**	**192**				
**12**	**SMc01312**	**FusA (f)**		**17**	**109**				
**13**	**SMc00324**	**Pnp (g)**	**Q92SW0**	**35**	**180**	**1.37**	**1.97**	Polyribonucleotide nucleotidyltransferase (EC 2.7.7.8).	J
**14**	**SMc00324**	**Pnp (g)**		**18**	**111**				
**15**	**SMc00324**	**Pnp (g)**		**20**	**129**				
**16**	**SMc00324**	**Pnp (g)**		**30**	**210**	**4.98**	**5.36**		
**17**	**SMc00324**	**Pnp (g)**		**20**	**111**	**4.98**	**5.40**		
**18**	**SMc03978**	**Tkt2**	**Q92M82**	**18**	**118**	**2.25**	**1.11**	Transketolase (EC 2.2.1.1).	G
**19**	**SMc00043**	**SodB**	**Q9XD74**	**48**	**82**	**1.60**	**0.28**	Superoxide dismutase [Mn] (EC 1.15.1.1).	P
**20**	**SMc02560**	**ChvI**	**P50350**	**32**	**68**	**1.86**	**1.23**	Transcriptional regulatory protein ChvI.	T-K
21	SMc01126	Tme	O30808	27	132	2.28	−0.27	NADP-dependent malic enzyme (EC 1.1.1.40).	C
**22**	**SMc00861**	**–**	**Q92KK7**	**14**	**62**	**2.00**	**0.40**	Putative signal peptide protein.	R
**23**	**SMc01326**	**TufB**	**Q925Y6**	**30**	**1,82**	**1.82**	**0.88**	Elongation factor Tu (EF-Tu).	J
**24**	**SMc02692**	**RplY**	**Q92N68**	**55**	**200**	**1.73**	**1.87**	Probable 50S ribosomal protein L25.	J
25	SMc00565	RplI	Q92QZ9	29	68	1.74	NA	Probable 50S ribosomal protein L9.	J
**26**	**SMc01333**	**PrfB**	**Q92QJ2**	**16**	**59**	**3.47**	**1.21**	Peptide chain release factor 2 (RF-2).	J
**27**	**SMc00357**	**Efp**	**Q92ST6**	**33**	**65**	**1.90**	**0.40**	Elongation factor P (EF-P).	J
**28**	**SMc01917**	**NuoE1**	**P56909**	**29**	**80**	**1.59**	**0.97**	NADH-quinone oxidoreductase chain E 1 (EC 1.6.99.5).	C
**29**	**SMc01845**	**–**	**Q92NK3**	**18**	**86**	**1.51**	**1.23**	Membrane-bound lytic mureintransglycosylase B precursor (EC 3.2.1.-).	M
–	SMc01285	RpoA	Q925Z2	31	93	SVNA	0.55	DNA-directed RNA polymerase alpha chain (EC 2.7.7.6).	K
–	SMc01318	RplL	Q92QH8	66	88	SVNA	0.47	50S ribosomal protein L7/L12.	J
–	SMc00335	RpsA	P14129	43	233	SVNA	1.86	30S ribosomal protein S1.	J
–	SMc01934	ProS	Q92QN2	53	168	SVNA	0.77	Putativeprolyl-tRNAsynthetase (EC 6.1.1.15).	J
–	SMc00480	Icd	Q92PG6	37	148	SVNA	0.49	Isocitrate dehydrogenase [NADP] (EC 1.1.1.42).	C
–	SMc01270	AdhC1	Q92QD7	37	144	SVNA	0.48	Alcohol dehydrogenase class III (EC 1.1.1.1).	C
–	SMc03826	ArgG	Q92L73	46	189	1.24	1.46	Argininosuccinatesynthase (EC 6.3.4.5).	E
–	SMc00643	PurA	Q92MA5	40	175	SVNA	1	Adenylosuccinatesynthetase (EC 6.3.4.4).	F
–	SMc02064	SerS	Q92Q22	41	150	SVNA	0.48	Seryl-tRNAsynthetase (EC 6.1.1.11).	J
–	SMc00641	SerA	Q92MA3	25	86	SVNA	0.71	D-3-phosphoglycerate dehydrogenase (EC 1.1.1.95) (PGDH).	E
–	SMc01192	MetS	Q92PX0	28	109	SVNA	0.26	Methionyl-tRNAsynthetase (EC 6.1.1.10).	J
–	SMc04461	TolB	Q926C2	42	162	SVNA	0.75	TolB protein precursor.	U
–	SMc02686	PrsA	Q92N73	24	55	SVNA	NA	Probable ribose-phosphate pyrophosphokinase.	F
–	SMc00153	—	Q92PB9	56	95	SVNA	1.80	Probable ATP synthase delta chain (EC 3.6.3.14).	S
–	SMc00595	NdK	Q92QX9	25	82	SVNA	0.88	Nucleoside diphosphatekinase (EC 2.7.4.6).	F
–	SMc01288	Adk	Q93FE6	55	124	SVNA	NA	Probable adenylatekinase.	F
–	SMc03242	TypA	Q92LG8	27	140	SVNA	3.39	GTP-binding protein typA/BipA.	T
–	SMc03925	Pgm	Q92M12	31	81	SVNA	1.39	Phosphoglucomutase (EC 5.4.2.2).	G
–	SMc02100	Tsf	Q92Q54	62	174	SVNA	−0.19	Elongation factor Ts (EF-Ts).	J
–	SMc02163	Pgi	Q92SC4	28	101	SVNA	1.43	Glucose-6-phosphate isomerase (EC 5.3.1.9).	G
–	SMc02495	Tal	Q92LK3	38	80	SVNA	1.70	Probable transaldolase (EC 2.2.1.2).	G
–	SMc00040	—	Q92RC9	27	67	SVNA	1.04	Hypothetical protein.	O
–	SMc01233	Ssb	P56898	64	189	SVNA	NA	Probable single-strand binding protein.	L
–	SMc01345	AccC	Q92QK1	30	117	SVNA	0.25	Biotin carboxylase (EC 6.3.4.14).	I

a: The code numbers (first column) and protein names (third column) correspond to those as in Fig. S2 and Fig. 1A, respectively. The polypeptides from each spot were identified by means of UV-MALDI-TOF MS and the peptide-mass fingerprint as described in Materials and Methods. The bold data corresponds to polypeptides detected as overexpressed both by the *ImageMaster *2D^™^ (GE) software (using 50% volume difference compared to the spot a pH 7.0, and *p* test lower than 0.05 in a *t*-test), and by (*a priori*) comparative visual inspection of the 2D gels (*i*.*e*. by the comparison of spots from gels shown in Fig. 1A,B).

b: Ratios of spot intensities (spot intensity at pH 6.1 *vs*. spot intensity of the homolog spot at pH 7.0) as estimated by the spot volume function of the *ImageMaster* 2D^™^ software.

c–g: The same letters correspond to different isoforms and/or/modifications or degradation products of a same protein detected at different positions in the 2D gel from Fig. 1A (or its replicates).

h: M values correspond to the log_2_ of the relative transcriptional activity of the indicated gene at pH 7.0 compared to the corresponding activity at pH 6.1, as listed in Supplementary Table S1.

i: COG classes as previously defined elsewhere^103^. *Cf*. consolidated COG analysis from the differential proteome and transcriptome in Fig. 2.

NA: Transcriptome data not available.

SVNA: “Spot volume not available” when using the ImageMaster sofware.

ON: Polypeptides that are present only in the cytoplasm of rhizobia grown at pH 6.1 (absent in the homologous sample from rhizobia grown at pH 7.0).

**Table 3:** *S. meliloti* 2011 protein markers differentially overexpressed under continuous cultivation of the rhizobia at extracellular pH 7.0 compared with the expression of the same markers at pH 6.1.

Gel Code (a)	ORF	Protein name (a)	Accession number	Squence Coverage (%)	Score (MASCOT)	Ratio of intensities (pH 7.0/pH 6.1) (b)	M Value (h) (transcriptome)	Predicted/putative function	COG (i)
**1**	**SMa2361**	**—**	**Q92XH5**	**43**	**162**	**ON**	**−3.05**	Hypothetical protein.	R
**2**	**SMc00786**	**DppA1**	**Q92RW0**	**18**	**87**	**2.21**	**−1.37**	Periplasmic dipeptide transport protein precursor.	E
**3**	**SMc01525**	**DppA2 (c)**	**Q92N26**	**41**	**201**	**5.46**	**−0.77**	Periplasmic oligopeptide-binding protein precursor.	E
**4**		**DppA2 (c)**		**46**	**156**			Periplasmic oligopeptide-binding protein precursor.	E
**5**		**DppA2 (c)**		**42**	**220**			Periplasmic oligopeptide-binding protein precursor.	E
**6**		**DppA2 (c)**		**41**	**168**			Periplasmic oligopeptide-binding protein precursor.	E
**7**	**SMb21196**	**OppA**	**Q9AKR0**	**25**	**72**	**SVNA**	**NA**	ABC transporter of tetrapeptides and some tripeptides, periplasmic solute-binding protein.	E
**8**	**SMc01946**	**LivK (d)**	**Q926C5**	**47**	**144**	**30.25**	**−2.58**	Leucine- isoleucine- valine- threonine- and alanine-binding protein precursor.	E
**9**		**LivK (d)**		**31**	**132**			Leucine- isoleucine- valine- threonine- and alanine-binding protein precursor.	E
**10**	**SMc00242**	**—**	**Q92PR4**	**27**	**87**	**ON**	**NA**	Hypothetical signal peptide protein.	R
**11**	**SMc01827**	**—**	**Q92K04**	**18**	**57**	**ON**	**−2.58**	Hypothetical protein.	P
**12**	**SMb20605**	**—**	**Q926E2**	**26**	**93**	**ON**	**−4.80**	Aliphatic amidase expression-regulating protein.	E
**13**	**SMb20745**	**GlnII**	**Q92TR3**	**20**	**58**	**5.00**	**−2.71**	Glutamine synthetase II (EC 6.3.1.2).	E
**14**	**SMb20964**	**—**	**Q9R9R2**	**38**	**101**	**1.44**	**NA**	Peroxidase	P
**15**	**SMc04023**	**ExoN2**	**Q92M48**	**25**	**110**	**ON**	**−0.40**	UTP-glucose-1-phosphate uridylyltransferase (EC 2.7.7.9)	M
16	SMa1353	—	Q92YX3	27	70	4.55	−0.58	D-tagatose 3-epimerase (EC 5.3.1.-).	G
17	SMc02896	IlvE1	Q92SX8	23	59	ON	1.12	Probable branched-chain amino acid aminotransferase (EC 2.6.1.42).	E-H
18	SMc03061	AglE	Q9Z3R5	16	88	ON	0.53	Alpha-glucosides-binding periplasmic protein AglE precursor.	G
**19**	**SMb20292**	**—**	**Q92WP7**	**51**	**143**	**1.51**	**−1.42**	Hypothetical immunogenic protein.	R
20	SMa1507	—	Q92YP3	33	80	ON	−1.75	Transcriptional activator protein precursor.	R
–	SMc02356	LivA	Q92MNO	25	111	SVNA	−1.57	Putative branched chain amino acid binding periplasmic ABC transporter.	E-T
–	SMc02873	—	Q92SZ7	34	102	SVNA	−0.41	Multiple sugar-binding protein precursor.	G
–	SMc02118	AapJ	Q92Q71	48	187	SVNA	−2.82	General L-amino acid-binding periplasmic protein.	E-T
–	SMc03786	Bfr	Q92LA7	55	110	SVNA	−1.51	Bacterioferritin (BFR).	P
–	SMc02259	—	Q92S63	46	146	SVNA	−1.14	Lysine-arginine-ornithine-binding periplasmic protein precursor.	E-T
–	SMb21144	—	Q926G9	33	233	SVNA	−0.56	Putative choline uptake ABC transporter periplasmic solute-binding protein precursor.	M
–	SMc01605	—	Q92NI5	37	161	SVNA	−1.17	Putative periplasmic binding ABC transporter protein.	P
–	SMb20915	AslA1	Q92UC0	34	161	SVNA	−2.46	Putative arylsulfatase.	P
–	SMc00140	—	Q92PA9	55	125	SVNA	−1.30	Arginine-binding periplasmic protein 2 precursor.	E-T
–	SMc01642	PrbA	Q92NF1	27	97	SVNA	−1.38	Periplasmic dipeptide transportprotein precursor.	E
–	SMc00777	ThrC1	Q92RF5	38	116	SVNA	−0.45	Probable threonine synthase.	S

a: The code numbers (first column) and protein names (third column) correspond to those as in Fig. S3 and Fig. 1B, respectively. The polypeptides from each spot were identified by means of UV-MALDI-TOF MS and the peptide-mass fingerprint as described in Materials and Methods. Bold data corresponds to polypeptides detected as overexpressed both by the *ImageMaster* 2D™ (GE) software (using 50% volume difference compared to the spot a pH 6.1, and *p* test lower than 0.05 in a *t*-test), and by (*a priori*) comparative visual inspection of the 2D gels (i.e. by the comparison of spots from gels shown in Fig. 1A,B).

b: Ratios of spot intensities (spot intensity at pH 7.0 *vs*. spot intensity of the homolog spot at pH 6.1) as estimated by the spot volume function of the *ImageMaster* 2D™ software.

c–g: The same letters correspond to different isoforms and/or modifications or degradation products of a same protein detected at different positions in the 2D gel from Fig. 1B (or its replicates).

h: M values correspond to the log_2_ of the relative transcriptional activity of the indicated gene at pH 7.0 compared to the corresponding activity at pH 6.1, as listed in Supplementary Table S1.

i: COG classes as previously defined elsewhere^103^. *Cf*. consolidated COG analysis from the differential proteome and transcriptome in Fig. 2.

NA: Transcriptome data not available.

SVNA: “Spot volume not available” when using the ImageMaster sofware.

ON: Polypeptides that are present only in the cytoplasm of only rhizobia grown at pH 7.0 (absent in the homologous sample from rhizobia grown at pH 6.1).

## References

[b1] Chen W. M. *et al.* Legume symbiotic nitrogen fixation by beta-proteobacteria is widespread in nature. J. Bacteriol. 185, 7266–7272 (2003). 14645288 10.1128/JB.185.24.7266-7272.2003 PMC296247

[b2] MacLean A. M., Finan T. M. & Sadowsky M. J. Genomes of the symbiotic nitrogen-fixing bacteria of legumes. Plant Physiol. 144, 615–622 (2007). 17556525 10.1104/pp.107.101634 PMC1914180

[b3] Trinick M. J. & Hadobas P. A. Biology of the *Parasponia-Bradyrhizobium* symbiosis. Plant Soil 110, 177–185, doi: 10.1007/bf02226797 (1988).

[b4] Giller K. E. & Wilson K. J. Nitrogen Fixation in Tropical Cropping Systems. 313 (CAB International, 1991).

[b5] O’Hara G. W. The Role of Nitrogen Fixation in Crop Production. Journal of Crop Production 1, 115–138, doi: 10.1300/J144v01n02_05 (1998).

[b6] Graham P. H. & Vance C. P. Nitrogen fixation in perspective: an overview of research and extension needs. Field Crops Research 65, 93–106 (2000).

[b7] Oldroyd G. E. & Dixon R. Biotechnological solutions to the nitrogen problem. Curr. Opin. Biotechnol. 26, 19–24 (2014). 24679253 10.1016/j.copbio.2013.08.006

[b8] Zahran H. H. Rhizobium-Legume Symbiosis and Nitrogen Fixation under Severe Conditions and in an Arid Climate. Microbiol. Mol. Biol. Rev. 63, 968–989 (1999). 10585971 10.1128/mmbr.63.4.968-989.1999 PMC98982

[b9] Glenn A. R., Reeve W. G., Tiwari R. P. & Dilworth M. J. Acid tolerance in root nodule bacteria. Novartis Found Symp 221, 112–126, discussion 126–130 (1999). 10207916

[b10] von Uexküll H. R. & Mutert E. Global extent, development and economic impact of acid soils. Plant Soil 171, 1–15, doi: 10.1007/bf00009558 (1995).

[b11] Howieson J. G. & Ewing M. A. Acid-tolerance in the *Rhizobium meliloti-Medicago* symbiosis. Aust J Agric Res 37, 55–64 (1986).

[b12] Howieson J. G., Robson A. D. & Abbott L. K. Acid-tolerant species of *Medicago* produce root exudates at low pH which induce the expression of nodulation genes in *Rhizobium meliloti*. Aust. J. Plant Physiol. 19, 287–296 (1992).

[b13] Lowendorf H. S., Baya A. M. & Alexander M. Survival of *Rhizobium* in Acid Soils. Appl. Environ. Microbiol. 42, 951–957 (1981). 16345909 10.1128/aem.42.6.951-957.1981 PMC244139

[b14] Caetano-Anollés G., Lagares A. & Favelukes G. Adsorption of *Rhizobium meliloti* to alfalfa roots: Dependence on divalent cations and pH. Plant Soil 117, 67–74 (1989).

[b15] Munns D. N. Nodulation of *Medicago sativa* in solution culture. Plant Soil 28, 129–146 (1968).

[b16] Rice W. A., Penney D. C. & Nyborg M. Effects of soil acidity on rhizobia numbers, nodulation and nitrogen fixation by alfalfa and red clover. Can. J. Soil Sci. 57, 197–203 (1977).

[b17] Vargas A. & Graham P. Cultivar and pH effects on competition for nodule sites between isolates of *Rhizobium* in beans. Plant Soil 117, 195–200 (1989).

[b18] Vassileva V., Milanov G., Ignatov G. & Nikolov B. Effect of low pH on nitrogen fixation of common bean grown at various calcium and nitrate levels. J Plant Nutr 20, 279–294, doi: 10.1080/01904169709365250 (1997).

[b19] Moron B. *et al.* Low pH changes the profile of nodulation factors produced by *Rhizobium tropici* CIAT899. Chem. Biol. 12, 1029–1040 (2005). 16183027 10.1016/j.chembiol.2005.06.014

[b20] Richardson A. E., Simpson R. J., Djordjevic M. A. & Rolfe B. G. Expression of Nodulation Genes in *Rhizobium leguminosarum* biovar *trifolii* Is Affected by Low pH and by Ca and Al Ions. Appl. Environ. Microbiol. 54, 2541–2548 (1988). 16347761 10.1128/aem.54.10.2541-2548.1988 PMC204310

[b21] Graham P. H. Stress tolerance in *Rhizobium* and *Bradyrhizobium*, and nodulation under adverse soil condition. Can. J. Microbiol. 38, 475–484 (1992).

[b22] Reeve W. G., Tiwari R. P., Dilworth M. J. & Glenn A. R. Calcium affects the growth and survival of *Rhizobium meliloti*. Soil Biol. Biochem. 25, 581–586 (1993).

[b23] Del Papa M. F. *et al.* Isolation and characterization of alfalfa-nodulating rhizobia present in acidic soils of central Argentina and Uruguay. Appl. Environ. Microbiol. 65, 1420–1427 (1999). 10103231 10.1128/aem.65.4.1420-1427.1999 PMC91201

[b24] Howieson J., Ewing M. & D’Antuono M. Selection for acid tolerance in *Rhizobium meliloti*. Plant Soil 105, 179–188 (1988).

[b25] Del Papa M. F. *et al.* A microcosm study on the influence of pH and the host-plant on the soil persistence of two alfalfa-nodulating rhizobia with different saprophytic and symbiotic characteristics. Biol Fertil Soils 39, 112–116 (2003).

[b26] Graham P. H. *et al.* Acid pH tolerance in strains of *Rhizobium* and *Bradyrhizobium*, and initial studies on the basis for acid tolerance of *Rhizobium tropici* UMR1899. Can. J. Microbiol. 40, 198–207 (1994).

[b27] Richardson A. E. & Simpson R. J. Acid-tolerance and symbiotic effectiveness of *Rhizobium trifolii* associated with a *Trifolium subterraneum* L.-based pasture growing in an acid soil. Soil Biol. Biochem. 21, 87–96 (1989).

[b28] Segundo E., Martinez-Abarca F., Dillewijn P., Fernández-López M., Lagares A., Martinez-Drets G., Niehaus K., Pühler A. & Toro. N. Characterisation of symbiotically efficient alfalfa-nodulating rhizobia isolated from acid soils of Argentina and Uruguay. FEMS Microbiol Ecol 28, 169–176 (1999).

[b29] Thornton F. & Davey C. Saprophytic competence of acid tolerant strains of *Rhizobium trifolii* in acid soil. Plant Soil 80, 337–344 (1984).

[b30] Tiwari R. P., Reeve W. G., Dilworth M. J. & Glenn A. R. An essential role for *actA* in acid tolerance of *Rhizobium meliloti*. Microbiology 142, 601–610 (1996). 8868435 10.1099/13500872-142-3-601

[b31] Tiwari R. P., Reeve W. G., Dilworth M. J. & Glenn A. R. Acid tolerance in *Rhizobium meliloti* strain WSM419 involves a two-component sensor-regulator system. Microbiology 142, 1693–1704 (1996). 8757734 10.1099/13500872-142-7-1693

[b32] Fenner B. J., Tiwari R. P., Reeve W. G., Dilworth M. J. & Glenn A. R. *Sinorhizobium medicae* genes whose regulation involves the ActS and/or ActR signal transduction proteins. FEMS Microbiol. Lett. 236, 21–31, doi: 10.1016/j.femsle.2004.05.016 (2004). 15212786

[b33] Reeve W. G., Tiwari R. P., Kale N. B., Dilworth M. J. & Glenn A. R. ActP controls copper homeostasis in *Rhizobium leguminosarum* bv. *viciae* and *Sinorhizobium meliloti* preventing low pH-induced copper toxicity. Mol. Microbiol. 43, 981–991 (2002). 11936079 10.1046/j.1365-2958.2002.02791.x

[b34] Vivas-Marfisi A., Tiwari R., Dilworth M. & Glenn A. In Nitrogen Fixation: From Molecules to Crop Productivity Vol. 38 Current Plant Science and Biotechnology in Agriculture (eds Pedrosa Fabio O., Hungria Mariangela, Yates Geoffrey, Newton William E.) Ch. 272, 487–487 (Springer: Netherlands, , 2000).

[b35] Reeve W. G., Tiwari R. P., Wong C. M., Dilworth M. J. & Glenn A. R. The transcriptional regulator gene phrR in *Sinorhizobium meliloti* WSM419 is regulated by low pH and other stresses. Microbiology 144, 3335–3342 (1998). 9884225 10.1099/00221287-144-12-3335

[b36] Vinuesa P. *et al.* Genetic analysis of a pH-regulated operon from *Rhizobium tropici* CIAT899 involved in acid tolerance and nodulation competitiveness. Mol. Plant. Microbe Interact. 16, 159–168, doi: 10.1094/MPMI.2003.16.2.159 (2003). 12575750

[b37] Reeve W. G. *et al.* The *Sinorhizobium medicae* WSM419 *lpiA* gene is transcriptionally activated by FsrR and required to enhance survival in lethal acid conditions. Microbiology 152, 3049–3059, doi: 10.1099/mic.0.28764-0 (2006). 17005985

[b38] Foster J. W. & Hall H. K. Adaptive acidification tolerance response of *Salmonella typhimurium*. J. Bacteriol. 172, 771–778 (1990). 2404956 10.1128/jb.172.2.771-778.1990 PMC208505

[b39] Goodson M. & Rowbury R. J. Resistance of acid-habituated *Escherichia coli* to organic acids and its medical and applied significance. Lett. Appl. Microbiol. 8, 211–214, doi: 10.1111/j.1472-765X.1989.tb00250.x (1989).

[b40] Dilworth M. J., Rynne F. G., Castelli J. M., Vivas-Marfisi A. I. & Glenn A. R. Survival and exopolysaccharide production in *Sinorhizobium meliloti* WSM419 are affected by calcium and low pH. Microbiology 145, 1585–1593, doi: 10.1099/13500872-145-7-1585 (1999). 10439397

[b41] Draghi W. O. *et al.* Cultural conditions required for the induction of an adaptive acid-tolerance response (ATR) in *Sinorhizobium meliloti* and the question as to whether or not the ATR helps rhizobia improve their symbiosis with alfalfa at low pH. FEMS Microbiol. Lett. 302, 123–130 (2010). 19958387 10.1111/j.1574-6968.2009.01846.x

[b42] O’Hara G. W. & Glenn A. R. The adaptive acid tolerance response in root nodule bacteria and *Escherichia coli*. Arch. Microbiol. 161, 286–292 (1994). 8002711 10.1007/BF00303582

[b43] Reeve W. G. *et al.* Probing for pH-regulated proteins in *Sinorhizobium medicae* using proteomic analysis. J Mol Microbiol Biotechnol 7, 140–147 (2004). 15263818 10.1159/000078657

[b44] Hellweg C., Puhler A. & Weidner S. The time course of the transcriptomic response of *Sinorhizobium meliloti* 1021 following a shift to acidic pH. BMC Microbiology 9, 37 (2009). 19216801 10.1186/1471-2180-9-37 PMC2651895

[b45] Watkin E. L. J., O’hara W. G. W. & Glenn A. R. Physiological responses to acid stress of an acid-soil tolerant and an acid-soil sensitive of *Rhizobium leguminosarum* bv. *trifolii*. Soil Biol. Biochem. 35, 621–624 (2003).

[b46] Hoskisson P. A. & Hobbs G. Continuous culture – making a comeback? Microbiology 151, 3153–3159, doi: 10.1099/mic.0.27924-0 (2005). 16207900

[b47] O’Hara G. W., Goss T. J., Dilworth M. J. & Glenn A. R. Maintenance of Intracellular pH and Acid Tolerance in *Rhizobium meliloti*. Appl. Environ. Microbiol. 55, 1870–1876 (1989). 16347984 10.1128/aem.55.8.1870-1876.1989 PMC202972

[b48] Galibert F. *et al.* The composite genome of the legume symbiont *Sinorhizobium meliloti*. Science 293, 668–672 (2001). 11474104 10.1126/science.1060966

[b49] Djordjevic M. A. *et al.* A global analysis of protein expression profiles in *Sinorhizobium meliloti*: discovery of new genes for nodule occupancy and stress adaptation. Mol. Plant. Microbe Interact. 16, 508–524 (2003). 12795377 10.1094/MPMI.2003.16.6.508

[b50] Deuerling E. *et al.* Trigger Factor and DnaK possess overlapping substrate pools and binding specificities. Mol. Microbiol. 47, 1317–1328, doi: 3370 [pii] (2003). 12603737 10.1046/j.1365-2958.2003.03370.x

[b51] Stark H. *et al.* Visualization of elongation factor Tu on the *Escherichia coli* ribosome. Nature 389, 403–406 (1997). 9311785 10.1038/38770

[b52] Aoki H., Adams S. L., Turner M. A. & Ganoza M. C. Molecular characterization of the prokaryotic *efp* gene product involved in a peptidyltransferase reaction. Biochimie 79, 7–11 (1997). 9195040 10.1016/s0300-9084(97)87619-5

[b53] Frank J. & Agrawal R. K. Ratchet-like movements between the two ribosomal subunits: their implications in elongation factor recognition and tRNA translocation. Cold Spring Harb Symp Quant Biol 66, 67–75 (2001). 12762009 10.1101/sqb.2001.66.67

[b54] Craigen W. J., Lee C. C. & Caskey C. T. Recent advances in peptide chain termination. Mol. Microbiol. 4, 861–865 (1990). 2215213 10.1111/j.1365-2958.1990.tb00658.x PMC7168415

[b55] Mars R. A., Mendonca K., Denham E. L. & van Dijl J. M. The reduction in small ribosomal subunit abundance in ethanol-stressed cells of *Bacillus subtilis* is mediated by a SigB-dependent antisense RNA. Biochim. Biophys. Acta 1853, 2553–2559, doi: 10.1016/j.bbamcr.2015.06.009 (2015). 26115952

[b56] Wang J. *et al.* Expression changes of ribosomal proteins in phosphate- and iron-deficient *Arabidopsis* roots predict stress-specific alterations in ribosome composition. BMC genomics 14, 783, doi: 10.1186/1471-2164-14-783 (2013). 24225185 PMC3830539

[b57] Zhang X. *et al.* Translational control of the cytosolic stress response by mitochondrial ribosomal protein L18. Nature structural & molecular biology 22, 404–410, doi: 10.1038/nsmb.3010 (2015). PMC4424103 25866880

[b58] Rawlings N. D. & Barrett A. J. Families of serine peptidases. Methods Enzymol 244, 19–61 (1994). 7845208 10.1016/0076-6879(94)44004-2 PMC7133253

[b59] Glazebrook J., Ichige A. & Walker G. C. Genetic analysis of *Rhizobium meliloti bacA-phoA* fusion results in identification of *degP*: two loci required for symbiosis are closely linked to *degP*. J. Bacteriol. 178, 745–752 (1996). 8550509 10.1128/jb.178.3.745-752.1996 PMC177721

[b60] Briani F. *et al.* Genetic analysis of polynucleotide phosphorylase structure and functions. Biochimie 89, 145–157 (2007). 17084501 10.1016/j.biochi.2006.09.020

[b61] Yu F., Inouye S. & Inouye M. Lipoprotein-28, a cytoplasmic membrane lipoprotein from *Escherichia coli*. Cloning, DNA sequence, and expression of its gene. J. Biol. Chem. 261, 2284–2288 (1986). 3003106

[b62] Foster S. J. Cloning, expression, sequence analysis and biochemical characterization of an autolytic amidase of *Bacillus subtilis* 168 *trpC2*. J. Gen. Microbiol. 137, 1987–1998 (1991). 1683402 10.1099/00221287-137-8-1987

[b63] Krogh S., Jorgensen S. T. & Devine K. M. Lysis genes of the *Bacillus subtilis* defective prophage PBSX. J. Bacteriol. 180, 2110–2117 (1998). 9555893 10.1128/jb.180.8.2110-2117.1998 PMC107137

[b64] Cheng H. P. & Walker G. C. Succinoglycan production by *Rhizobium meliloti* is regulated through the ExoS-ChvI two-component regulatory system. J. Bacteriol. 180, 20–26 (1998). 9422587 10.1128/jb.180.1.20-26.1998 PMC106843

[b65] Yuan Z. C., Liu P., Saenkham P., Kerr K. & Nester E. W. Transcriptome profiling and functional analysis of *Agrobacterium tumefaciens* reveals a general conserved response to acidic conditions (pH 5.5) and a complex acid-mediated signaling involved in *Agrobacterium*-plant interactions. J. Bacteriol. 190, 494–507 (2008). 17993523 10.1128/JB.01387-07 PMC2223696

[b66] Wells D. H., Chen E. J., Fisher R. F. & Long S. R. ExoR is genetically coupled to the ExoS-ChvI two-component system and located in the periplasm of *Sinorhizobium meliloti*. Mol. Microbiol. 64, 647–664 (2007). 17462014 10.1111/j.1365-2958.2007.05680.x

[b67] Belanger L. & Charles T. Members of the *Sinorhizobium meliloti* ChvI regulon identified by a DNA binding screen. BMC Microbiology 13, 132 (2013). 23758731 10.1186/1471-2180-13-132 PMC3687685

[b68] Sauviac L. & Bruand C. A putative bifunctional histidine kinase/phosphatase of the HWE family exerts positive and negative control on the *Sinorhizobium meliloti* general stress response. J. Bacteriol. 196, 2526–2535, doi: 10.1128/JB.01623-14JB.01623-14[pii] (2014). 24794560 PMC4097584

[b69] Griffitts J. S. *et al.* A *Sinorhizobium meliloti* osmosensory two-component system required for cyclic glucan export and symbiosis. Mol. Microbiol. 69, 479–490 (2008). 18630344 10.1111/j.1365-2958.2008.06304.x

[b70] Wu C. F., Lin J. S., Shaw G. C. & Lai E. M. Acid-induced type VI secretion system is regulated by ExoR-ChvG/ChvI signaling cascade in *Agrobacterium tumefaciens*. PLoS Pathogens 8, e1002938, doi: 10.1371/journal.ppat.1002938 (2012). 23028331 PMC3460628

[b71] Stauffer L. T. & Stauffer G. V. Antagonistic Roles for GcvA and GcvB in *hdeAB* Expression in *Escherichia coli*. ISRN microbiology 2012, 697308 (2012). 23762759 10.5402/2012/697308 PMC3658693

[b72] Owttrim G. W. RNA helicases: diverse roles in prokaryotic response to abiotic stress. RNA biology 10, 96–110, doi: 10.4161/rna.22638 (2013). 23093803 PMC3590241

[b73] Geddes B. A. & Oresnik I. J. Physiology, genetics, and biochemistry of carbon metabolism in the alphaproteobacterium *Sinorhizobium meliloti*. Can. J. Microbiol. 60, 491–507, doi: 10.1139/cjm-2014-0306 (2014). 25093748

[b74] Weissenmayer B., Gao J. L., Lopez-Lara I. M. & Geiger O. Identification of a gene required for the biosynthesis of ornithine-derived lipids. Mol. Microbiol. 45, 721–733 (2002). 12139618 10.1046/j.1365-2958.2002.03043.x

[b75] Sohlenkamp C. & Geiger O. Bacterial membrane lipids: diversity in structures and pathways. FEMS Microbiol. Rev., doi: 10.1093/femsre/fuv008 (2015). 25862689

[b76] Barnett M. J. *et al.* Nucleotide sequence and predicted functions of the entire *Sinorhizobium meliloti* pSymA megaplasmid. Proc. Natl. Acad. Sci. USA 98, 9883–9888, doi: 10.1073/pnas.161294798 (2001). 11481432 PMC55547

[b77] Tiwari R. P. *et al.* Probing for pH-regulated genes in *Sinorhizobium medicae* using transcriptional analysis. J Mol Microbiol Biotechnol 7, 133–139 (2004). 15263817 10.1159/000078656

[b78] Bekker M., de Vries S., Ter Beek A., Hellingwerf K. J. & de Mattos M. J. Respiration of *Escherichia coli* can be fully uncoupled via the nonelectrogenic terminal cytochrome bd-II oxidase. J. Bacteriol. 191, 5510–5517, doi: 10.1128/JB.00562-09 (2009). 19542282 PMC2725625

[b79] Portais J. C., Tavernier P., Gosselin I. & Barbotin J. N. Cyclic organization of the carbohydrate metabolism in *Sinorhizobium meliloti*. Eur. J. Biochem. 265, 473–480 (1999). 10491206 10.1046/j.1432-1327.1999.00778.x

[b80] Janczarek M. Environmental signals and regulatory pathways that influence exopolysaccharide production in rhizobia. Int J Mol Sci 12, 7898–7933, doi: 10.3390/ijms12117898 (2011). 22174640 PMC3233446

[b81] Crabtree H. G. Observations on the carbohydrate metabolism of tumours. The Biochemical journal 23, 536–545 (1929). 16744238 10.1042/bj0230536 PMC1254097

[b82] Pronk J. T., Yde Steensma H. & Van Dijken J. P. Pyruvate metabolism in *Saccharomyces cerevisiae*. Yeast 12, 1607–1633 (1996). 9123965 10.1002/(sici)1097-0061(199612)12:16<1607::aid-yea70>3.0.co;2-4

[b83] Paczia N. *et al.* Extensive exometabolome analysis reveals extended overflow metabolism in various microorganisms. Microbial cell factories 11, 122, doi: 10.1186/1475-2859-11-122 (2012). 22963408 PMC3526501

[b84] Wolfe A. J. The Acetate Switch. Microbiol. Mol. Biol. Rev. 69, 12–50, doi: 10.1128/mmbr.69.1.12-50.2005 (2005). 15755952 PMC1082793

[b85] Doelle H., Ewings K. & Hollywood N. In Microbial Reactions Vol. 23 Advances in Biochemical Engineering Ch. 1, 1–35 (Springer: Berlin Heidelberg, , 1982).

[b86] Geddes B. A., González J. E. & Oresnik I. J. Exopolysaccharide Production in Response to Medium Acidification Is Correlated With an Increase in Competition for Nodule Occupancy. Mol. Plant. Microbe Interact. 27, 1307–1317, doi: 10.1094/mpmi-06-14-0168-r (2014). 25387133

[b87] Pierre O. *et al.* Peribacteroid space acidification: a marker of mature bacteroid functioning in *Medicago* truncatula nodules. Plant Cell Environ 36, 2059–2070, doi: 10.1111/pce.12116 (2013). 23586685

[b88] Roux B. *et al.* An integrated analysis of plant and bacterial gene expression in symbiotic root nodules using laser-capture microdissection coupled to RNA sequencing. The Plant Journal 77, 817–837, doi: 10.1111/tpj.12442 (2014). 24483147

[b89] Evans C. G. T., Herbert D. & Tempest D. W. The continuous cultivation of micro-organisms. II. Construction of a chemostat. Methods Microbiol 2, 277–327 (1970).

[b90] Cooney C. L., Wang H. Y. & Wang D. I. C. Computer-aided material balancing for prediction of fermentation parameters. Biotechnol. Bioeng. 19, 55–67 (1977). 321044 10.1002/bit.260190106

[b91] Solorzano L. Determination of ammonia in natural waters by the phenolhypochlorite method. Limnol. Oceanogr. 14, 799 (1969).

[b92] Luna M. F., Mignone C. F. & Boiardi J. L. The carbon source influences the energetic efficiency of the respiratory chain of N_2_-fixing *Acetobacter diazotrophicus*. Appl. Microbiol. Biotechnol. 54, 564–569 (2000). 11092633 10.1007/s002530000425

[b93] Trevelyan W. E., Forrest R. S. & Harrison J. S. Determination of Yeast Carbohydrates with the Anthrone Reagent. Nature 170, 626–627 (1952). 13002392 10.1038/170626a0

[b94] Law J. H. & Slepecky R. A. Assay of Poly-beta-hydroxybutyric acid. J. Bacteriol. 82, 33–36 (1961). 13759651 10.1128/jb.82.1.33-36.1961 PMC279110

[b95] Becker A. *et al.* Global changes in gene expression in *Sinorhizobium meliloti* 1021 under microoxic and symbiotic conditions. Mol. Plant. Microbe Interact. 17, 292–303 (2004). 15000396 10.1094/MPMI.2004.17.3.292

[b96] De Risi J. L., Iyer V. R. & Brown P. O. Exploring the metabolic and genetic control of gene expression on a genomic scale. Science 278, 680–686 (1997). 9381177 10.1126/science.278.5338.680

[b97] Ruberg S. *et al.* Construction and validation of a *Sinorhizobium meliloti* whole genome DNA microarray: genome-wide profiling of osmoadaptive gene expression. J Biotechnol 106, 255–268 (2003). 14651866 10.1016/j.jbiotec.2003.08.005

[b98] Yang Y. H. *et al.* Normalization for cDNA microarray data: a robust composite method addressing single and multiple slide systematic variation. Nucleic Acids Res. 30, e15-, doi: 10.1093/nar/30.4.e15 (2002). 11842121 PMC100354

[b99] Dondrup M. *et al.* EMMA: a platform for consistent storage and efficient analysis of microarray data. J Biotechnol 106, 135–146 (2003). 14651856 10.1016/j.jbiotec.2003.08.010

[b100] Edgar R., Domrachev M. & Lash A. E. Gene Expression Omnibus: NCBI gene expression and hybridization array data repository. Nucleic Acids Res 30, 207–210 (2002). 11752295 10.1093/nar/30.1.207 PMC99122

[b101] Barsch A., Patschkowski T. & Niehaus K. Comprehensive metabolite profiling of *Sinorhizobium meliloti* using gas chromatography-mass spectrometry. Funct Integr Genomics 4, 219–230 (2004). 15372312 10.1007/s10142-004-0117-y

[b102] Xia J., Mandal R., Sinelnikov I. V., Broadhurst D. & Wishart D. S. MetaboAnalyst 2.0–a comprehensive server for metabolomic data analysis. Nucleic Acids Res. 40, W127–133 (2012). 22553367 10.1093/nar/gks374 PMC3394314

[b103] Tatusov R. *et al.* The COG database: an updated version includes eukaryotes. BMC Bioinformatics 4, 41 (2003). 12969510 10.1186/1471-2105-4-41 PMC222959

