# Supplementary material for: A consolidated analysis of the physiologic and molecular responses induced under acid stress in the legume-symbiont model-soil bacterium Sinorhizobium meliloti
Source: Sci Rep. 2016 Jul 11;6:29278. doi: 10.1038/srep29278 (PMC4941405; doi:10.1038/srep29278)
Supplement: Supplementary Dataset 1 [file srep29278-s1.pdf]

A consolidated analysis of the physiologic and molecular responses induced under acid stress in the legume-symbiont model-soil bacterium *Sinorhizobium meliloti*.

Draghi, W. O.[1]; Del Papa, M. F. [1]; Hellweg, C.[2]; Watt, S. A.[2]; Watt, T. F.[2]; Barsch, A[2]. Lozano, M. J.[1]; Lagares, A. (Jr.)[4]; Salas, M. E[1]; López, J. L.[1]; Albicoro, F. J.[1]; Nilson, J. F.[1]; Torres Tejerizo, G. A.[1]; Luna, M. F. [3]; Pistorio, M.[1]; Boiardi, J. L.[3]; Pühler, A.[2]; Weidner; S.[2]; Niehaus, K.[2]; and Lagares A. [1]*

[1] IBBM - Instituto de Biotecnología y Biología Molecular, CONICET - Departamento de Ciencias Biológicas, Facultad de Ciencias Exactas, Universidad Nacional de La Plata, calles 47 y 115, 1900-La Plata, Argentina

[2] CeBiTec - Centrum für Biotechnologie, Universität Bielefeld, Bielefeld, Germany

[3] CINDEFI – Centro de Investigación y Desarrollo en Fermentaciones Industriales, CONICET - Departamento de Química, Facultad de Ciencias Exactas, Universidad Nacional de La Plata, calles 47 y 115, 1900-La Plata, Argentina

[4] Laboratorio de Bioquímica, Microbiología e Interacciones Biológicas en el Suelo, Departamento de Ciencia y Tecnología, Universidad Nacional de Quilmes, Roque Sáenz Peña 352, Bernal B1876BXD, Buenos Aires, Argentina

Key words: acid stress, rhizobia, legume

***Corresponding author**
Phone: +54-221-425-0497 ext. 31
Fax: +54-221-422-3409 ext. 56
E-mail:lagares@biol.unlp.edu.ar

<u>SUPPLEMENTARY DATA</u>

Fig. S1. Correlation analysis between the experimental and the expected (calculated) values of molecular weight (Panel B, pH 7.0 and Panel D, pH 6.1) and isolectric point (Panel A, pH 7.0 and Panel C, pH 6.1) of the protein markers identified by PMF in the gels shown in Figs. 1 and 2. The molecular weight and isoelectric-point (pI) values for each individual polypeptide were calculated from the corresponding amino-acid sequence (abscissa). Experimental values were inferred from the position in the 2-D gels by means of the *Image Master*™ software (*ordinate*). For most polypeptides a good correlation was obtained between the observed and the expected molecular weight and pI values.

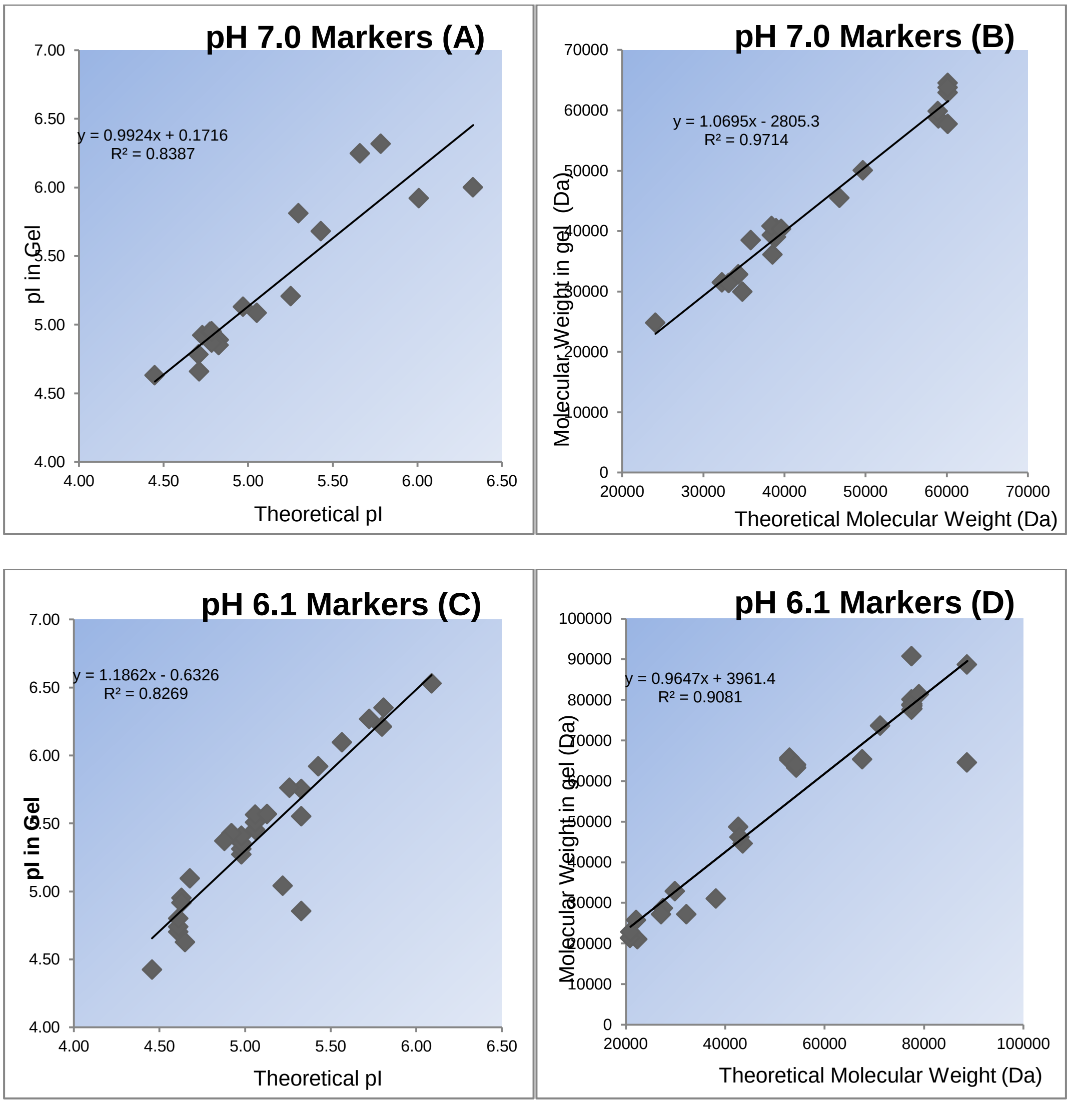

SUPPLEMENTARY DATA

Fig. S2. Image-Master detection of induced polypeptides in the cytosolic proteome of *S. meliloti* 2011 grown in chemostat at pH 6.1 compared to the expression of the corresponding polypeptides in the proteome of the same rhizobia grown at pH 7.0. The ratio of homologous polypeptides in one pH condition compared to the other was estimated by the ratio of the spot volumes (Image Master) after each one was normalized to the total intensity (all spots) from the corresponding gel. For each pH condition four independent gels were analyzed, including those from Figs.1 and 2. The numerical codes (in black) for each red-outlined spot in the figure (differentially expressed polypeptides) correspond to the respective numbers in the first column of Table 2.

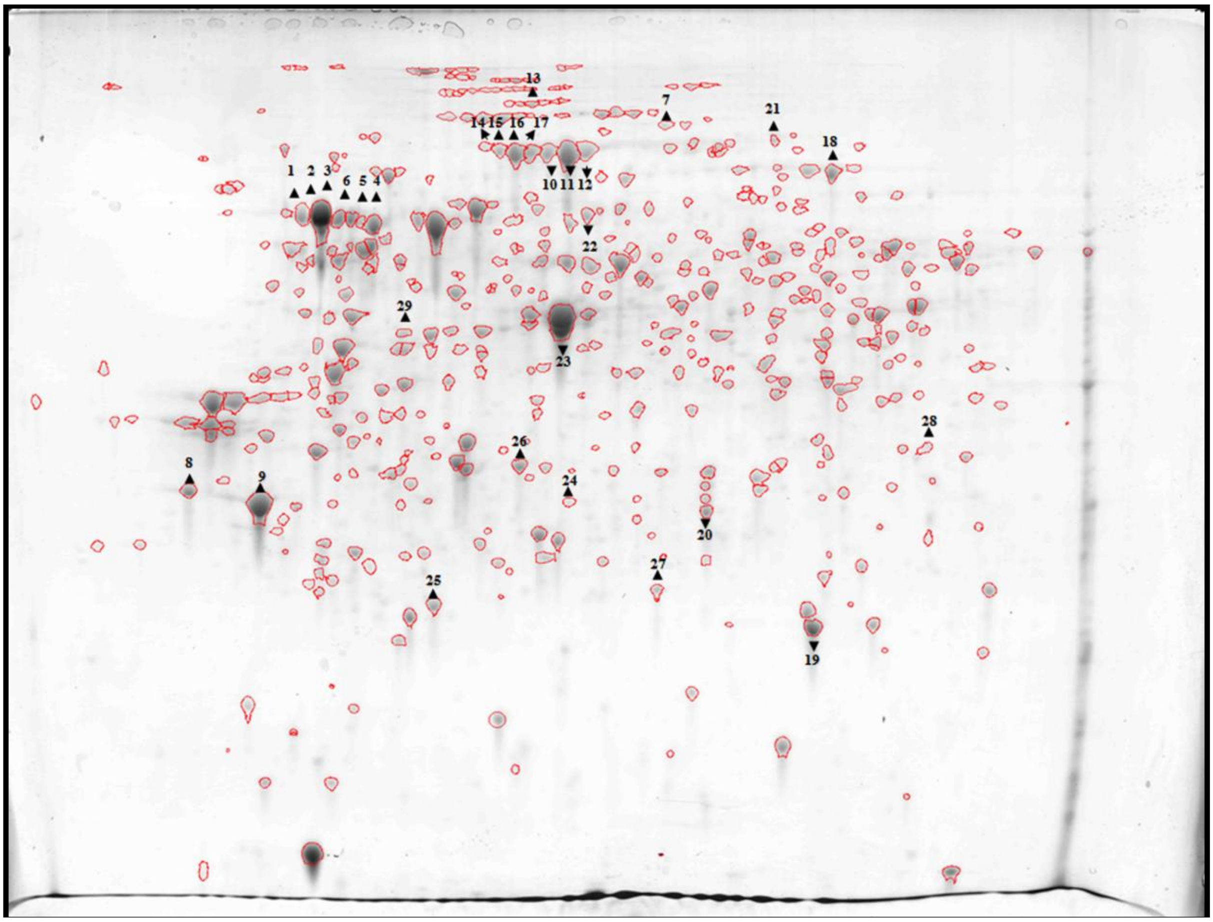

SUPPLEMENTARY DATA

Fig. S3. Image-Master detection of induced polypeptides in the cytosolic proteome of *S. meliloti* 2011 grown in chemostat at pH 7.0 compared to the expression of the corresponding polypeptides in the proteome of the same rhizobia grown at pH 6.1. The ratio of homologous polypeptides in one pH condition compared to the other was estimated by the ratio of the spot volumes (Image Master) after each one was normalized to the total intensity (all spots) from the corresponding gel. For each pH condition four independent gels were analyzed, including those from Figs.1 and 2. The numerical codes (in black) for each red-outlined spot in the figure (differentially expressed polypeptides) correspond to the respective numbers in the first column of Table 3.

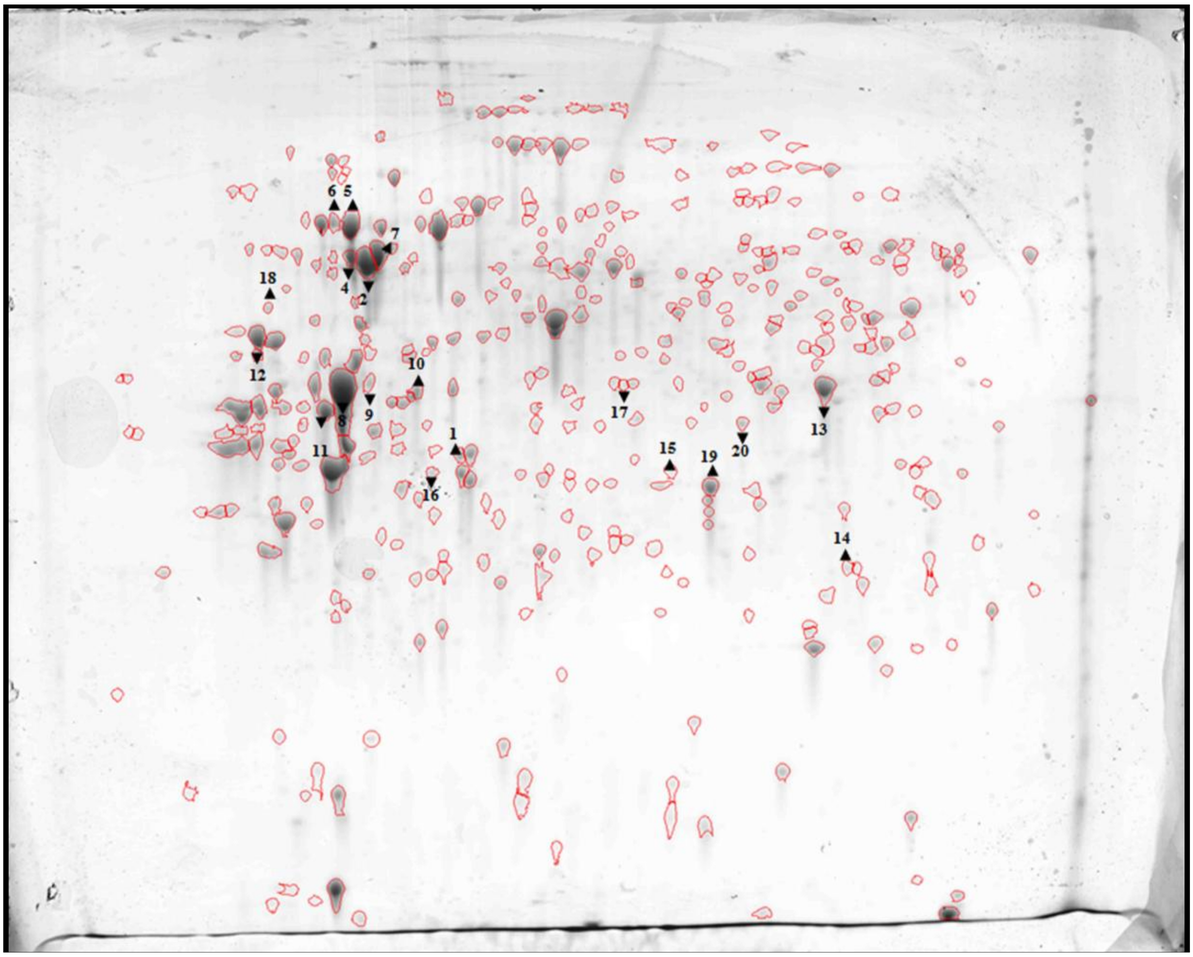

SUPPLEMENTARY DATA

Fig. S4. qRT-PCR estimation of 5S and 16S rRNAs in *S. meliloti* cells grown in the chemostat under steady state at pH 7.0, and at pH 6.1. The quantification of specific RNA species was carried out by quantitative reverse-transcription PCR using a KAPA SYBR FAST One-Step qRT-PCR kit (Kapa Biosystems Inc. Wilmington, MA, USA) according to the manufacturer's instructions. Analyses were performed using a same normalized amount of total RNA for all samples. M values represent the $\log_2$ of the fold change of the indicated RNA species at pH 6.1 (red bars) and 7.4 (blue bars) both with respect to their corresponding amounts at pH 7.0 (reference condition). 5S and 16S correspond to the analysis of 5S and 16S rRNA. *degP1* (SMc02365) corresponds to the analysis of the mRNA of an acid-induced gene used as positive control ($M^{pH\ 6.1\ /\ pH\ 7.0}$ from the microarrays = 1.98). The DegP1 polypeptide was also induced in the proteome of acid-grown rhizobia (fold change *ca.* 4.8 in Table 2, so with an estimated $M^{pH\ 6.1\ /\ pH\ 7.0}$ from the proteome = 2.26). The M values are the means calculated from three technical qRT-PCR replicas. The bars represent the standard deviations.

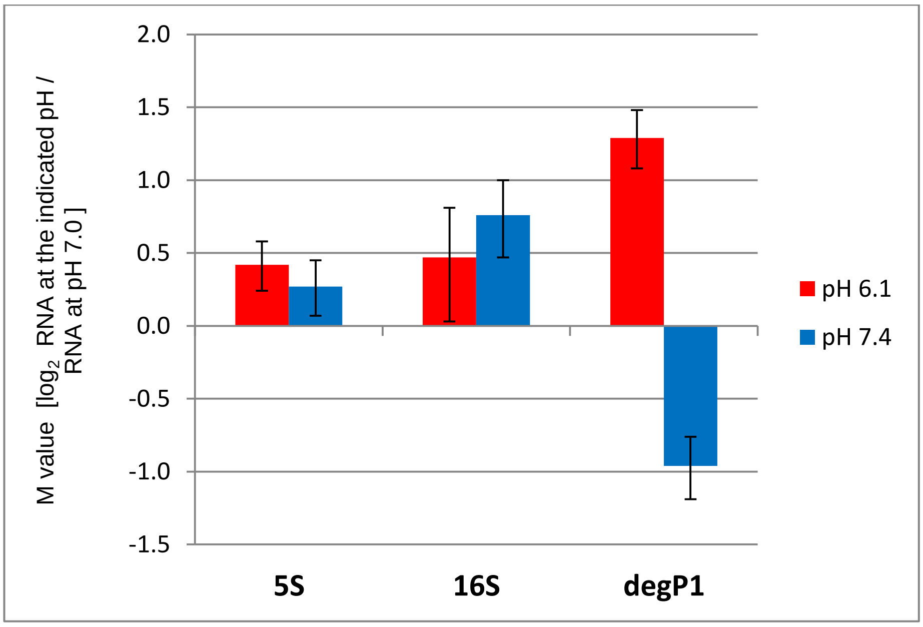

SUPPLEMENTARY DATA

Fig. S5. Genomic distribution of proteome and transcriptome markers differentially overexpressed at pH 6.1 *versus* 7.0. The two outermost circular array of lines on each replicon show—in black and in gray—the respective positions of open reading frames within the two complementary DNA strands. The next array inside shows—with red and blue lines—the genomic position of genes encoding polypeptides that were observed to be induced under acidic and neutral extracellular conditions, respectively (from the proteomic data). The innermost array marks—with green and orange lines—the genomic position of genes encoding RNAs that were also observed to be induced under acidic and neutral extracellular conditions, respectively (from the transcriptome data). The pSymA region characterized by a high density of overexpressed genes at pH 6.1 (*cf.* the text) is outlined and marked by an asterisk.

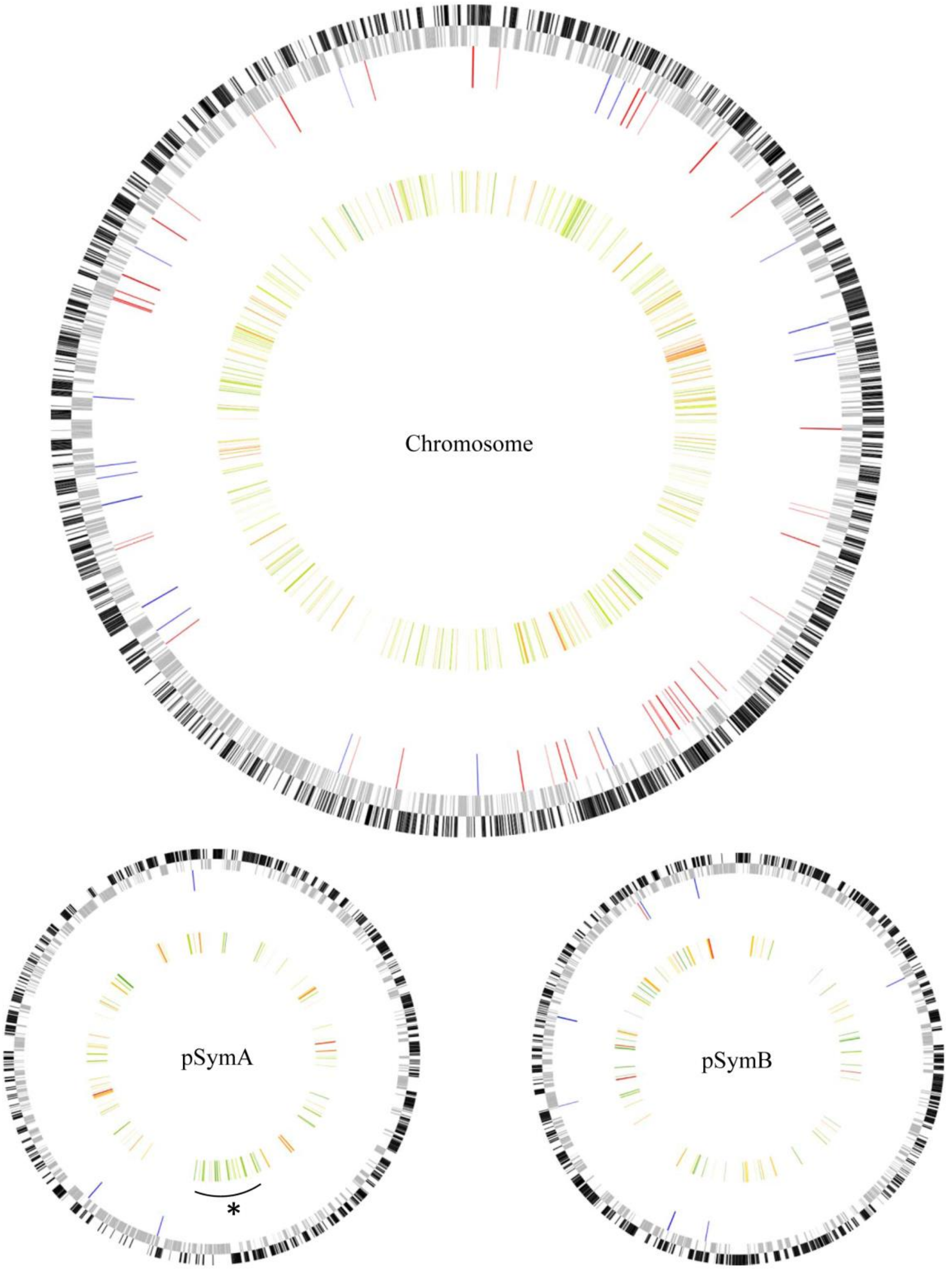
Chromosome
pSymA
*
pSymB

SUPPLEMENTARY DATA

Fig. S6. Vector-correlation plot between the variables examined (*i. e.*, the amount of metabolites) and the PC1 and PC2 spaces of PCA. The direction and length of the vectors are indicative of the positive contribution of each individual metabolite to the position of each sample analyzed in the plot (*i. e.*, from rhizobia grown at pH 6.1, pH 7.0, or pH 7.4).

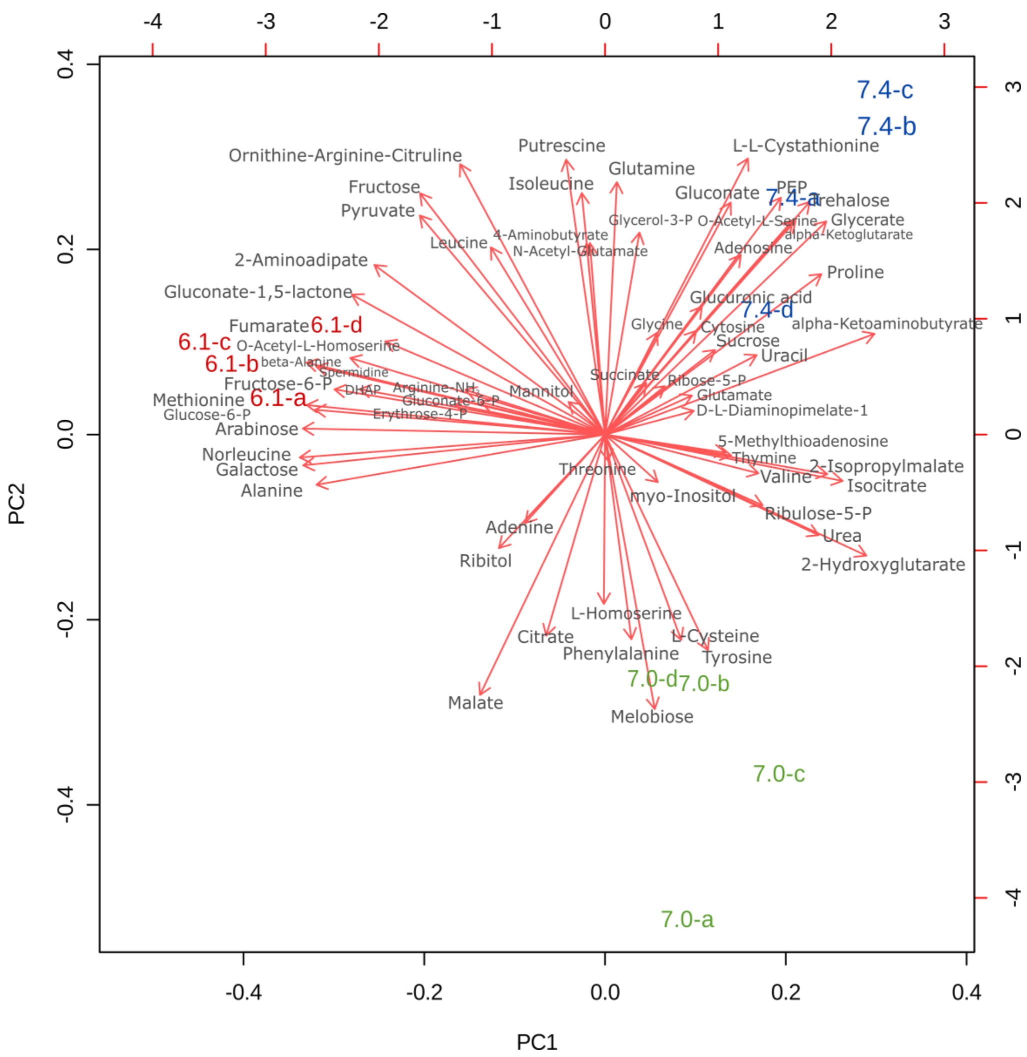

SUPPLEMENTARY DATA

Fig. S7. Graphical maps indicating significant increases of specific RNAs, polypeptides, or metabolites on different *S. meliloti* metabolic pathways (KEGG format); and also changes in RNAs/polypeptides related to cellular macromolecular complexes (transport, energy, translation). Colored rectangles: correspond to increased RNAs or polypeptides (i.e. RNAs listed in Supplementary Table 1, and polypeptides listed in the main Tables 1 and 2). Colored circles: correspond to increased metabolites (i.e. those listed in Supplementary Table 2, and also presented in the volcano plot from Fig. 3). **Red** and **blue** colors indicate increased amount of the corresponding species at **pH 6.1** and **pH 7.0**, respectively. References to panels are as follows: **A.** Pentose phosphate pathway; **B.** Galactose metabolism; **C.** Starch and sucrose metabolism; **D.** Carbon metabolism; **E.** Arginine and proline metabolism; **F.** Cysteine and methionine metabolism; **G.** Glutathione metabolism; **H.** Aminoacids biosynthesis; **I.** Purine metabolism; **J.** Pyrimidine metabolism; **K.** Biotin metabolism; **L.** Ribosome components; **M.** RNA degradation; **N.** O xidative phosphorylation; **O.** Flagellar assembly; **P.** Secretion systems; **Q.** Two-component systems; and **R.** ABC transporters.

A

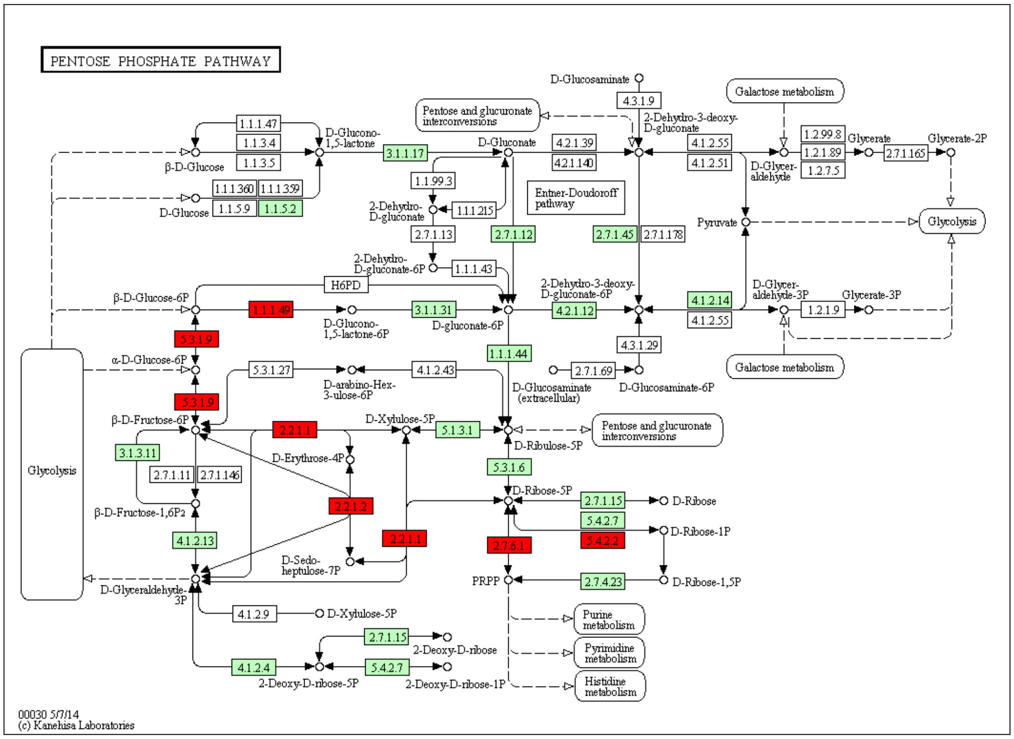

B

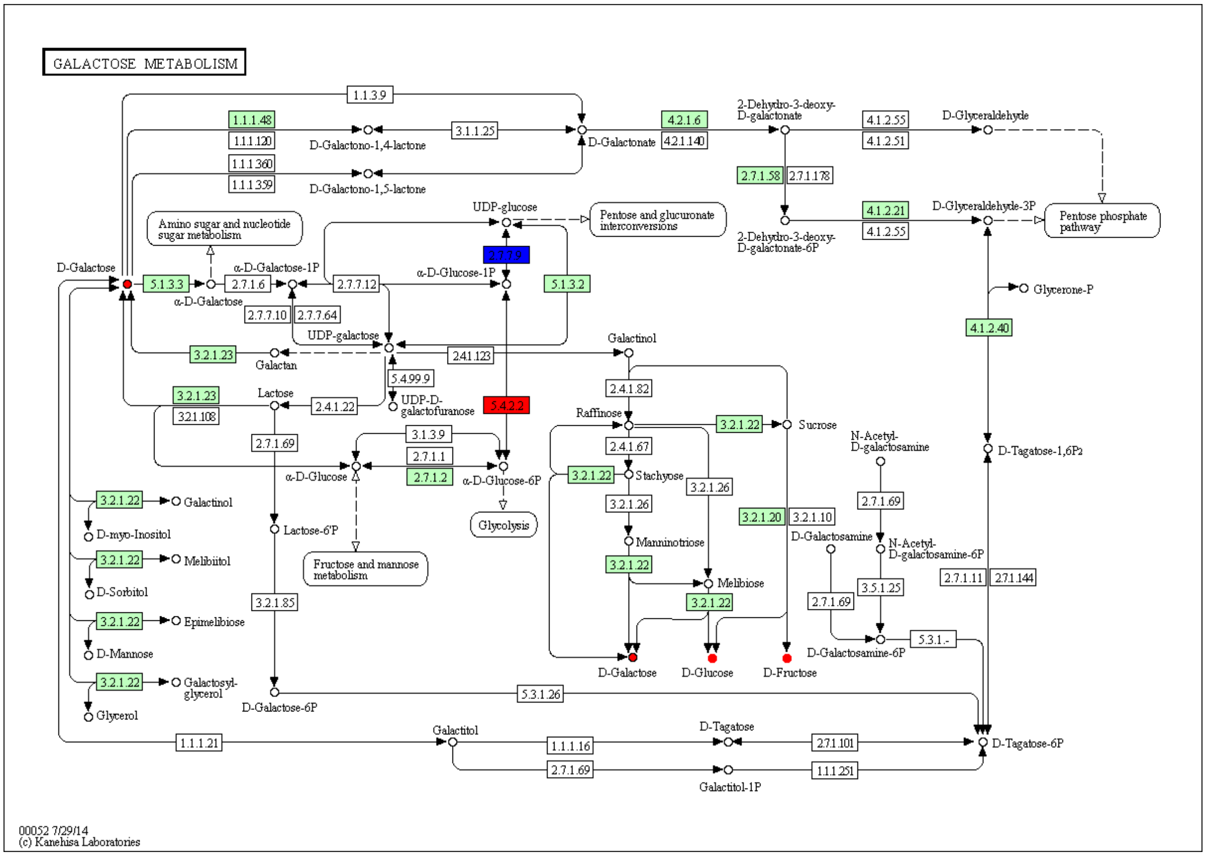
GALACTOSE METABOLISM
D-Galactono-1,4-lactone
D-Galactono-1,5-lactone
D-Galactonate
2-Dehydro-3-deoxy-D-galactonate
D-Glyceraldehyde
2-Dehydro-3-deoxy-D-galactonate-6P
D-Glyceraldehyde-3P
Pentose phosphate pathway
Glycerone-P
Amino sugar and nucleotide sugar metabolism
UDP-glucose
Pentose and glucuronate interconversions
D-Galactose
α-D-Galactose
α-D-Galactose-1P
α-D-Glucose-1P
UDP-galactose
Galactan
Galactinol
Lactose
UDP-D-galactofuranose
Raffinose
Sucrose
N-Acetyl-D-galactosamine
D-Tagatose-1,6P2
α-D-Glucose
α-D-Glucose-6P
Stachyose
Glycolysis
Galactinol
D-myo-Inositol
Lactose-6P
Fructose and mannose metabolism
Manninotriose
D-Galactosamine
N-Acetyl-D-galactosamine-6P
Melibiitol
D-Sorbitol
Melibiose
Epimelibiose
D-Mannose
D-Galactosamine-6P
D-Galactose
D-Glucose
D-Fructose
Galactosyl-glycerol
Glycerol
D-Galactose-6P
Galactitol
D-Tagatose
D-Tagatose-6P
Galactitol-1P
00052 7/29/14
(c) Kanehisa Laboratories

C

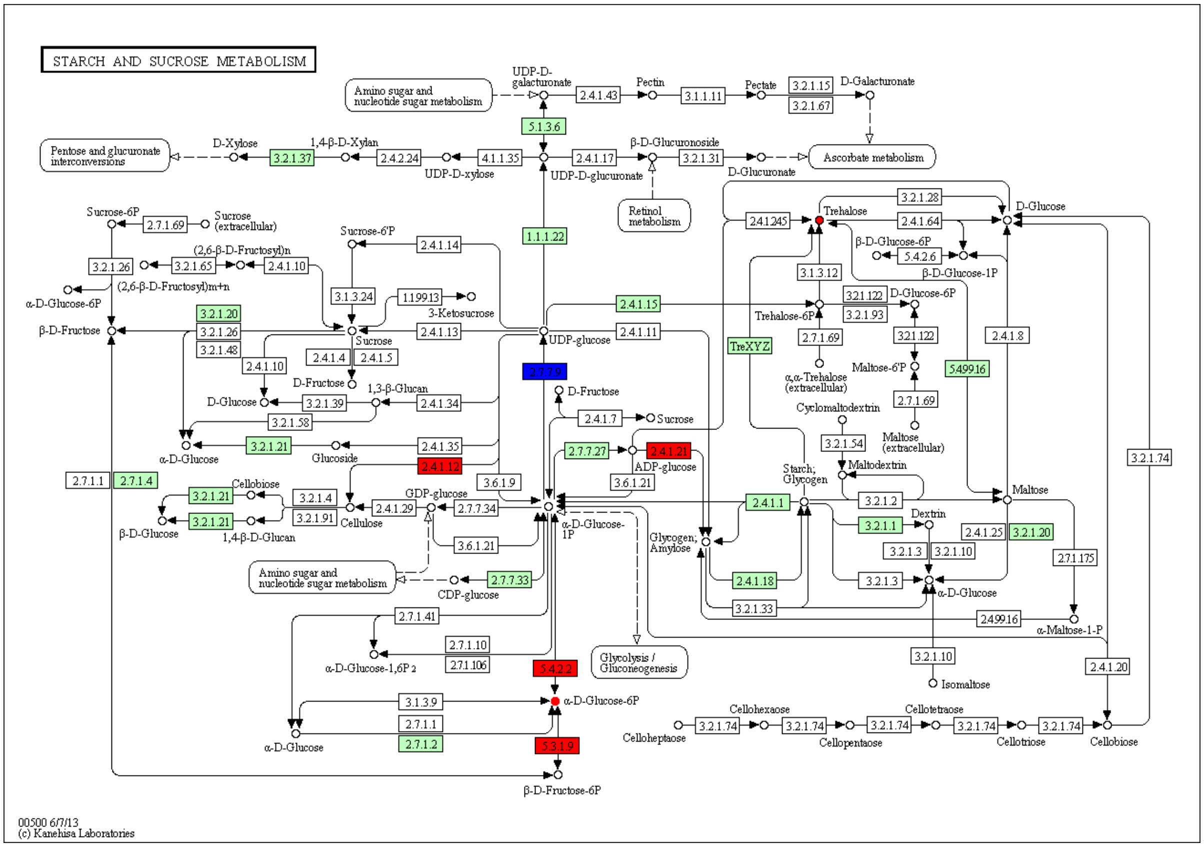
STARCH AND SUCROSE METABOLISM
Amino sugar and nucleotide sugar metabolism
UDP-D-galacturonate
Pectin
Pectate
D-Galacturonate
Pentose and glucuronate interconversions
D-Xylose
1,4-β-D-Xylan
UDP-D-xylose
UDP-D-glucuronate
β-D-Glucuronoside
D-Glucuronate
Ascorbate metabolism
Retinol metabolism
Sucrose-6P
Sucrose (extracellular)
Trehalose
D-Glucose
(2,6-β-D-Fructosyl)n
(2,6-β-D-Fructosyl)m+n
α-D-Glucose-6P
β-D-Fructose
β-D-Glucose-6P
β-D-Glucose-1P
3-Ketosucrose
Trehalose-6P
D-Glucose-6P
UDP-glucose
Sucrose
D-Fructose
1,3-β-Glucan
D-Glucose
α,α-Trehalose (extracellular)
Maltose-6P
Cyclomaltodextrin
Maltose (extracellular)
Maltodextrin
Glucoside
α-D-Glucose
ADP-glucose
Starch; Glycogen
Maltose
Cellobiose
GDP-glucose
β-D-Glucose
Cellulose
1,4-β-D-Glucan
α-D-Glucose-1P
Glycogen; Amylose
Dextrin
α-D-Glucose
Amino sugar and nucleotide sugar metabolism
CDP-glucose
α-Maltose-1-P
α-D-Glucose-1,6P2
Glycolysis / Gluconeogenesis
Isomaltose
α-D-Glucose-6P
Cellohexaose
Cellotetraose
Celloheptaose
Cellopentaose
Cellotriose
Cellobiose
α-D-Glucose
β-D-Fructose-6P
00500 6/7/13
(c) Kanehisa Laboratories

D

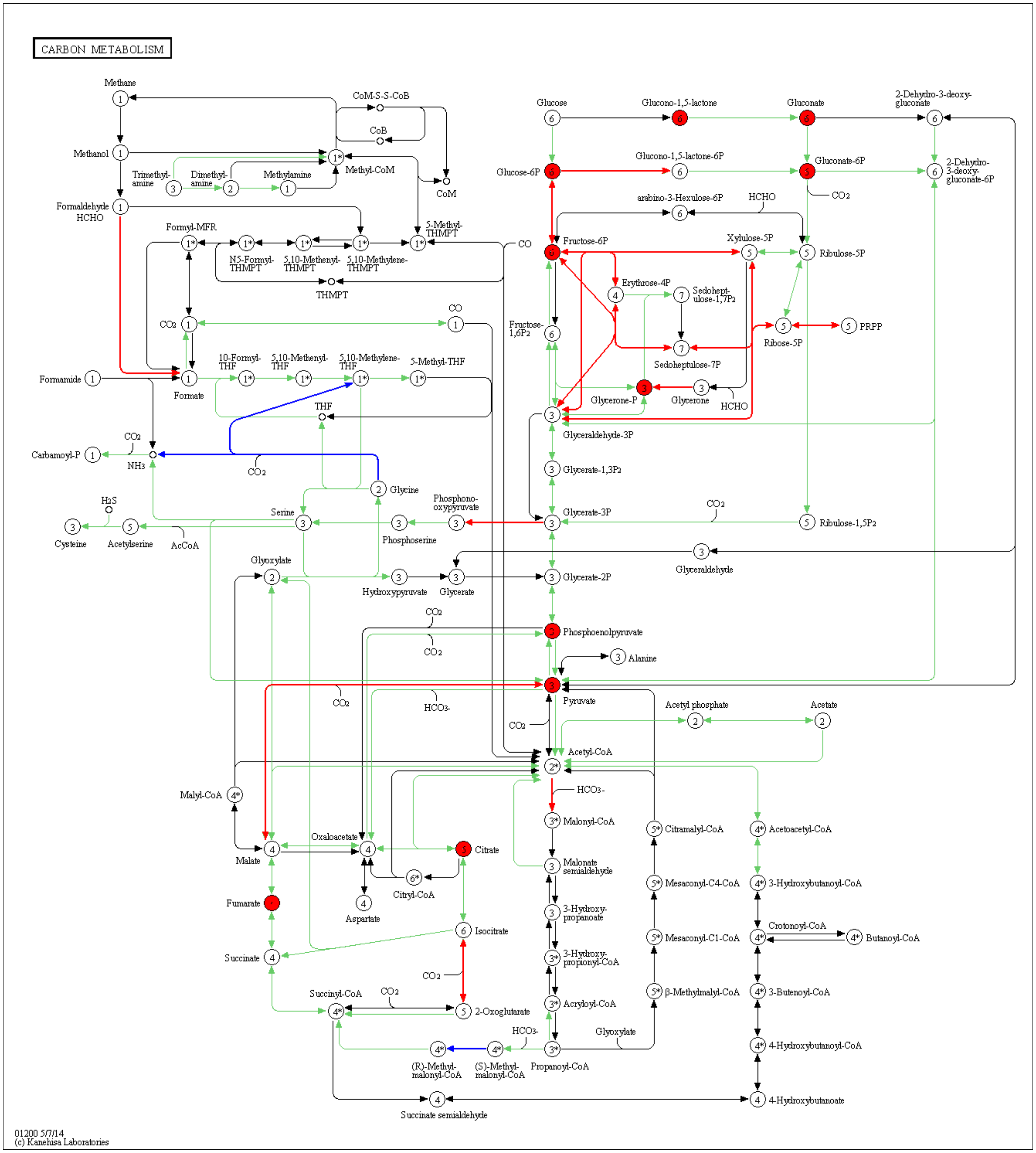
CARBON METABOLISM
Methane
CoM-S-S-CoB
CoB
Methanol
Methyl-CoM
Trimethyl-amine
Dimethyl-amine
Methylamine
CoM
Formaldehyde
HCHO
Formyl-MFR
5-Methyl-THMPT
N5-Formyl-THMPT
5,10-Methenyl-THMPT
5,10-Methylene-THMPT
CO
THMPT
CO2
CO
10-Formyl-THF
5,10-Methenyl-THF
5,10-Methylene-THF
5-Methyl-THF
Formamide
Formate
THF
CO2
Carbamoyl-P
NH3
CO2
Glycine
H2S
Serine
Phosphono-oxypyruvate
Cysteine
Acetylserine
AcCoA
Phosphoserine
Glyoxylate
Hydroxypyruvate
Glycerate
CO2
CO2
Glucose
Glucono-1,5-lactone
Gluconate
2-Dehydro-3-deoxy-gluconate
Glucose-6P
Glucono-1,5-lactone-6P
Gluconate-6P
2-Dehydro-3-deoxy-gluconate-6P
arabino-3-Hexulose-6P
HCHO
CO2
Fructose-6P
Xylulose-5P
Ribulose-5P
Erythrose-4P
Sedohept-ulose-1,7P2
Fructose-1,6P2
Ribose-5P
PRPP
Sedoheptulose-7P
Glycerone-P
Glycerone
HCHO
Glyceraldehyde-3P
Glycerate-1,3P2
CO2
Glycerate-3P
Ribulose-1,5P2
Glyceraldehyde
Glycerate-2P
Phosphoenolpyruvate
Alanine
Pyruvate
CO2
HCO3-
Acetyl phosphate
Acetate
CO2
Acetyl-CoA
HCO3-
Malyl-CoA
Malonyl-CoA
Citramalyl-CoA
Acetoacetyl-CoA
Oxaloacetate
Malate
Citrate
Malonate semialdehyde
Citryl-CoA
Mesaconyl-C4-CoA
3-Hydroxybutanoyl-CoA
Fumarate
Aspartate
3-Hydroxy-propanoate
Crotonoyl-CoA
Isocitrate
Mesaconyl-C1-CoA
Butanoyl-CoA
Succinate
3-Hydroxy-propionyl-CoA
CO2
Succinyl-CoA
CO2
2-Oxoglutarate
Acryloyl-CoA
β-Methylmalyl-CoA
3-Butenoyl-CoA
HCO3-
Glyoxylate
(R)-Methyl-malonyl-CoA
(S)-Methyl-malonyl-CoA
Propanoyl-CoA
4-Hydroxybutanoyl-CoA
Succinate semialdehyde
4-Hydroxybutanoate
01200 5/7/14
(c) Kanehisa Laboratories

E

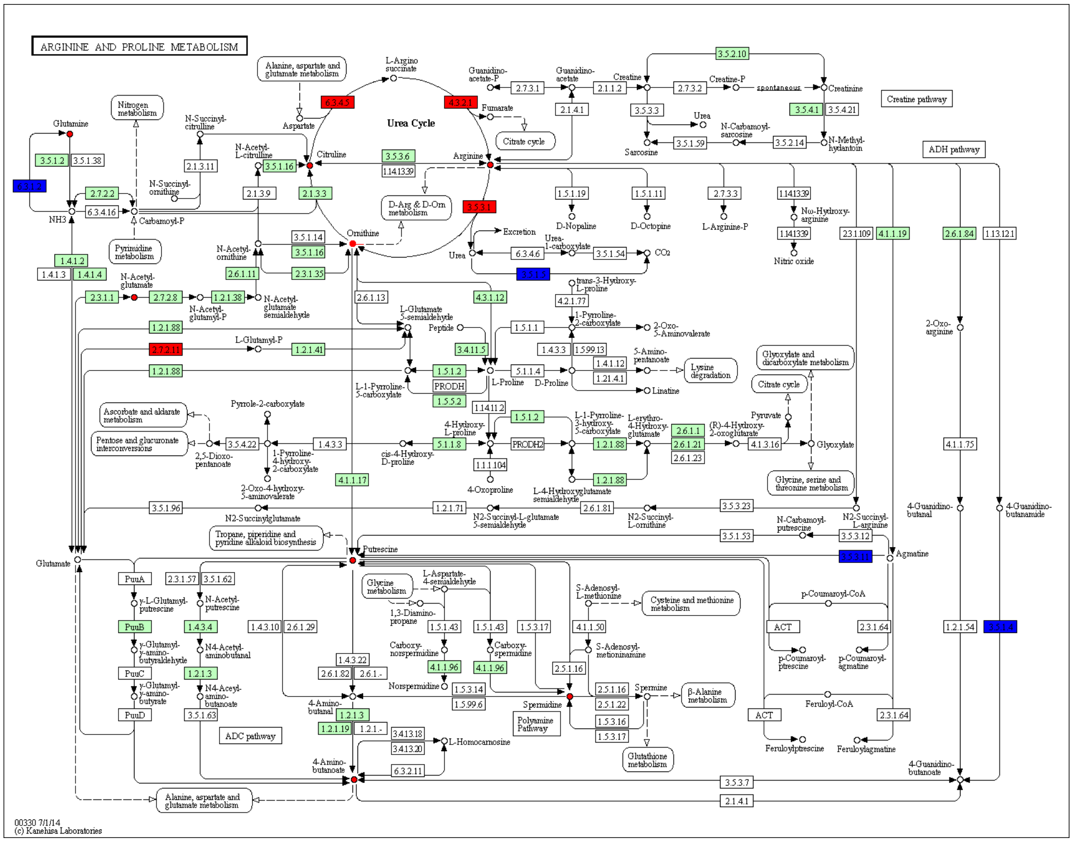
ARGININE AND PROLINE METABOLISM
Urea Cycle
Glutamine
Aspartate
L-Argino succinate
Fumarate
Citrulline
Arginine
Ornithine
Urea
Creatine
Creatine-P
Creatinine
Guanidino-acetate
Sarcosine
Creatine pathway
ADH pathway
Nitrogen metabolism
Pyrimidine metabolism
Carbamoyl-P
Citrate cycle
D-Arg & D-Orn metabolism
L-Glutamate 5-semialdehyde
L-Proline
D-Proline
Putrescine
Spermidine
Spermine
Agmatine
Glutamate
4-Amino-butanoate
Alanine, aspartate and glutamate metabolism
Glutathione metabolism
β-Alanine metabolism
Cysteine and methionine metabolism
Polyamine Pathway
ADC pathway
L-Homocarnosine
00330 7/1/14
(c) Kanehisa Laboratories

F

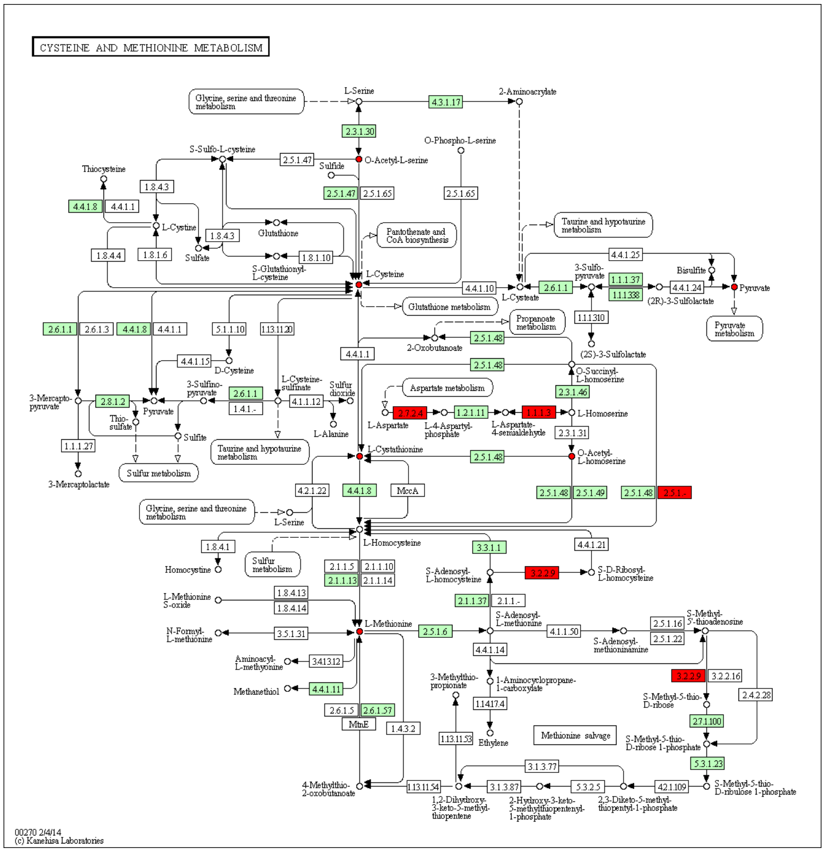
CYSTEINE AND METHIONINE METABOLISM
L-Serine
2-Aminoacrylate
Glycine, serine and threonine metabolism
S-Sulfo-L-cysteine
O-Acetyl-L-serine
O-Phospho-L-serine
Thiocysteine
L-Cystine
Glutathione
Pantothenate and CoA biosynthesis
Taurine and hypotaurine metabolism
L-Cysteine
L-Cysteate
3-Sulfo-pyruvate
Pyruvate
Pyruvate metabolism
Glutathione metabolism
Propanoate metabolism
2-Oxobutanoate
D-Cysteine
3-Mercapto-pyruvate
Sulfur metabolism
L-Aspartate
L-Homoserine
O-Acetyl-L-homoserine
O-Succinyl-L-homoserine
L-Cystathionine
L-Homocysteine
Homocysteine
S-Adenosyl-L-homocysteine
S-Adenosyl-L-methionine
L-Methionine
L-Methionine S-oxide
N-Formyl-L-methionine
Methanethiol
Ethylene
1-Aminocyclopropane-1-carboxylate
S-Methyl-5'-thioadenosine
S-Methyl-5-thio-D-ribose
Methionine salvage
4-Methylthio-2-oxobutanoate
00270 2/4/14
(c) Kanehisa Laboratories

G

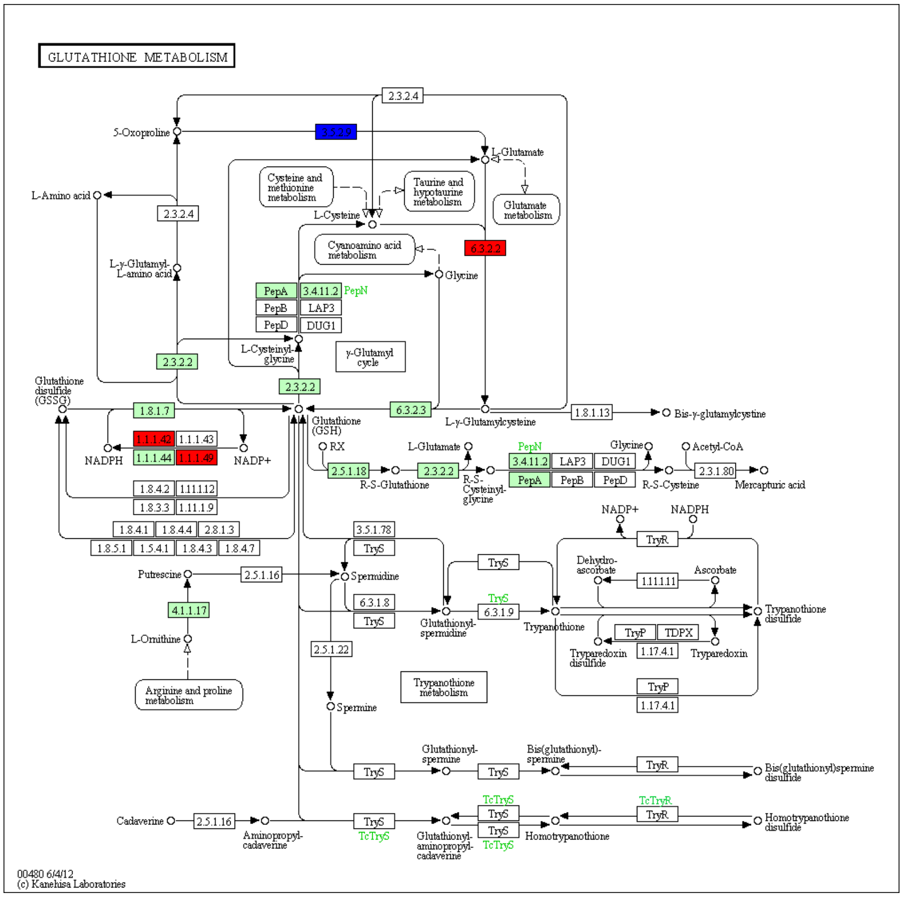
GLUTATHIONE METABOLISM
2.3.2.4
5-Oxoproline
3.5.2.9
L-Glutamate
Cysteine and methionine metabolism
Taurine and hypotaurine metabolism
Glutamate metabolism
L-Amino acid
2.3.2.4
L-Cysteine
Cyanoamino acid metabolism
6.3.2.2
L-γ-Glutamyl-L-amino acid
Glycine
PepA
3.4.11.2
PepN
PepB
LAP3
PepD
DUG1
L-Cysteinyl-glycine
γ-Glutamyl cycle
2.3.2.2
2.3.2.2
Glutathione disulfide (GSSG)
1.8.1.7
6.3.2.3
L-γ-Glutamylcysteine
1.8.1.13
Bis-γ-glutamylcystine
Glutathione (GSH)
1.11.1.42
1.1.1.43
1.1.1.44
1.1.1.49
NADPH
NADP+
RX
L-Glutamate
Glycine
Acetyl-CoA
2.5.1.18
2.3.2.2
R-S-Glutathione
R-S-Cysteinyl-glycine
PepN
3.4.11.2
LAP3
DUG1
PepA
PepB
PepD
R-S-Cysteine
2.3.1.80
Mercapturic acid
1.8.4.2
1.11.1.12
1.8.3.3
1.11.1.9
1.8.4.1
1.8.4.4
2.8.1.3
1.8.5.1
1.5.4.1
1.8.4.3
1.8.4.7
3.5.1.78
TryS
NADP+
NADPH
TryR
Putrescine
2.5.1.16
Spermidine
TryS
Dehydro-ascorbate
Ascorbate
1.11.1.11
4.1.1.17
6.3.1.8
TryS
TryS
6.3.1.9
Glutathionyl-spermidine
Trypanothione
Trypanothione disulfide
L-Ornithine
TryP
TDPX
1.17.4.1
Tryparedoxin disulfide
Tryparedoxin
2.5.1.22
Arginine and proline metabolism
Trypanothione metabolism
TryP
1.17.4.1
Spermine
Glutathionyl-spermine
Bis(glutathionyl)-spermine
TryS
TryS
TryR
Bis(glutathionyl)spermine disulfide
Cadaverine
2.5.1.16
TryS
TcTryS
TryS
TryS
TcTryS
TcTryR
TryR
Aminopropyl-cadaverine
TcTryS
Glutathionyl-aminopropyl-cadaverine
Homotrypanothione
Homotrypanothione disulfide
00480 6/4/12
(c) Kanehisa Laboratories

H

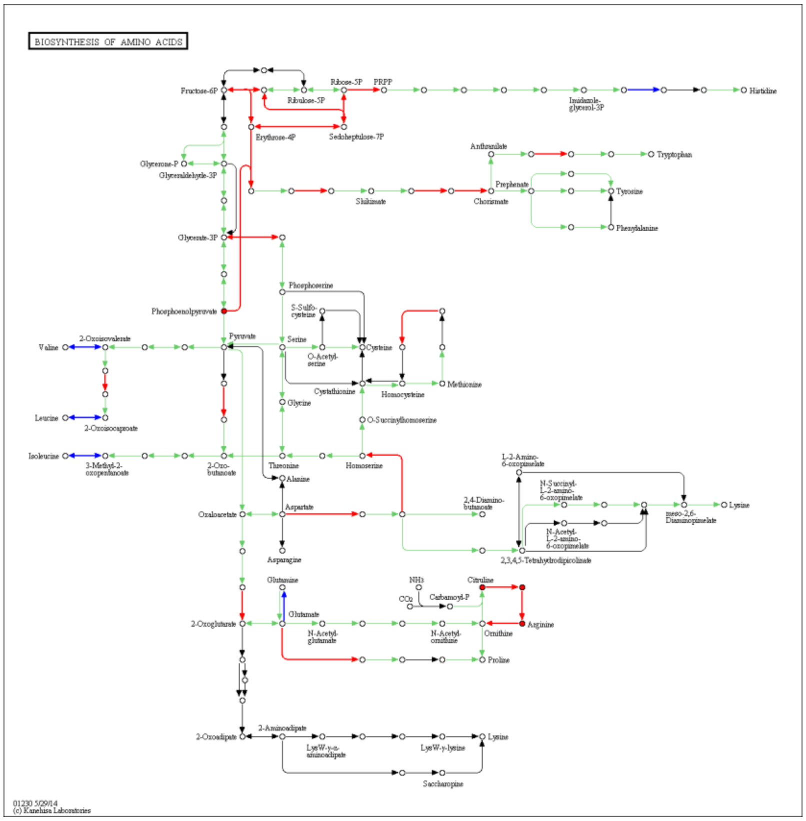
BIOSYNTHESIS OF AMINO ACIDS
Fructose-6P
Ribose-5P
PRPP
Ribulose-5P
Histidine
Imidazole-glycerol-3P
Erythrose-4P
Sedoheptulose-7P
Anthranilate
Tryptophan
Glycerone-P
Glyceraldehyde-3P
Prephenate
Tyrosine
Shikimate
Chorismate
Phenylalanine
Glycerate-3P
Phosphoserine
Phosphoenolpyruvate
S-Sulfo-cysteine
Pyruvate
Serine
Cysteine
2-Oxoisovalerate
Valine
O-Acetyl-serine
Methionine
Cystathionine
Homocysteine
Glycine
Leucine
2-Oxoisocaproate
O-Succinylhomoserine
Isoleucine
3-Methyl-2-oxopentanoate
2-Oxo-butanoate
Threonine
Homoserine
Alanine
L-2-Amino-6-oxopimelate
N-Succinyl-L-2-amino-6-oxopimelate
Aspartate
2,4-Diamino-butanoate
Lysine
Oxaloacetate
meso-2,6-Diaminopimelate
N-Acetyl-L-2-amino-6-oxopimelate
Asparagine
2,3,4,5-Tetrahydrodipicolinate
Glutamine
NH3
Citrulline
CO2
Carbamoyl-P
Glutamate
2-Oxoglutarate
Arginine
N-Acetyl-glutamate
N-Acetyl-ornithine
Ornithine
Proline
2-Aminoadipate
2-Oxoadipate
Lysine
LysW-γ-α-aminoadipate
LysW-γ-lysine
Saccharopine
01230 5/29/14
(c) Kanehisa Laboratories

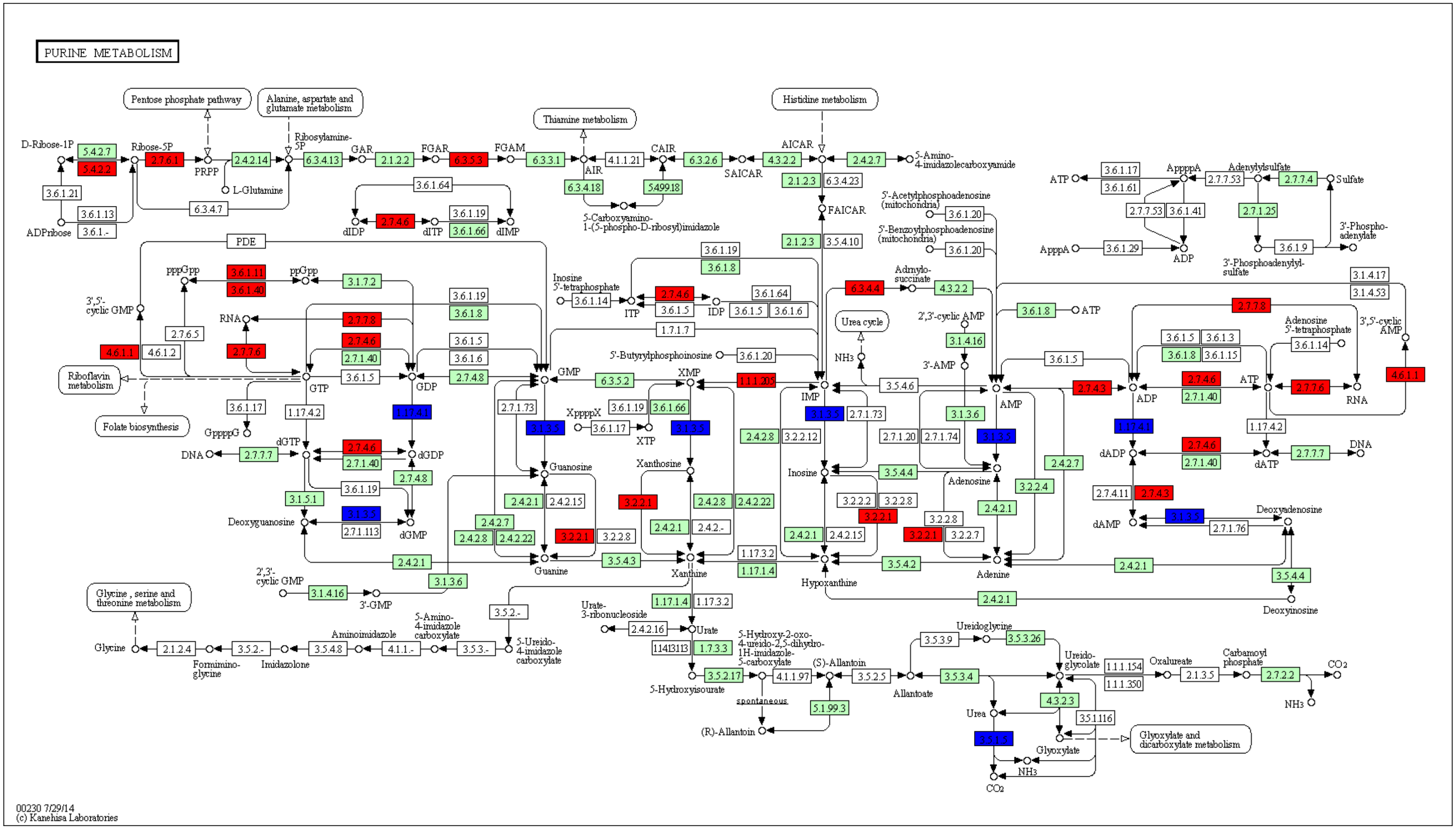
PURINE METABOLISM
Pentose phosphate pathway
Alanine, aspartate and glutamate metabolism
Thiamine metabolism
Histidine metabolism
D-Ribose-1P
Ribose-5P
PRPP
L-Glutamine
Ribosylamine-5P
GAR
FGAR
FGAM
AIR
CAIR
SAICAR
AICAR
5-Amino-4-imidazolecarboxyamide
FAICAR
5-Carboxyamino-1-(5-phospho-D-ribosyl)imidazole
ADPribose
dIDP
dITP
dIMP
PDE
ppGpp
pppGpp
3',5'-cyclic GMP
RNA
GTP
GDP
GMP
Riboflavin metabolism
Folate biosynthesis
GppppG
dGTP
dGDP
DNA
Deoxyguanosine
dGMP
Guanosine
Guanine
2',3'-cyclic GMP
3'-GMP
Inosine 5'-tetraphosphate
ITP
IDP
5'-Butyrylphosphoinosine
XMP
XppppX
XTP
Xanthosine
Xanthine
IMP
Inosine
Hypoxanthine
NH3
Urea cycle
Adenylo-succinate
2',3'-cyclic AMP
3'-AMP
AMP
Adenosine
Adenine
5'-Acetylphosphoadenosine (mitochondria)
5'-Benzoylphosphoadenosine (mitochondria)
ATP
ApppA
AppppA
ADP
Adenylylsulfate
Sulfate
3'-Phosphoadenylyl-sulfate
3'-Phospho-adenylate
Adenosine 5'-tetraphosphate
3',5'-cyclic AMP
dADP
dATP
dAMP
Deoxyadenosine
Deoxyinosine
Glycine, serine and threonine metabolism
Glycine
Formimino-glycine
Imidazolone
Aminoimidazole
5-Amino-4-imidazole carboxylate
5-Ureido-4-imidazole carboxylate
Urate-3-ribonucleoside
Urate
5-Hydroxyisourate
5-Hydroxy-2-oxo-4-ureido-2,5-dihydro-1H-imidazole-5-carboxylate
(S)-Allantoin
(R)-Allantoin
spontaneous
Allantoate
Ureidoglycine
Ureido-glycolate
Oxalureate
Carbamoyl phosphate
CO2
Urea
Glyoxylate
Glyoxylate and dicarboxylate metabolism
00230 7/29/14
(c) Kanehisa Laboratories

J

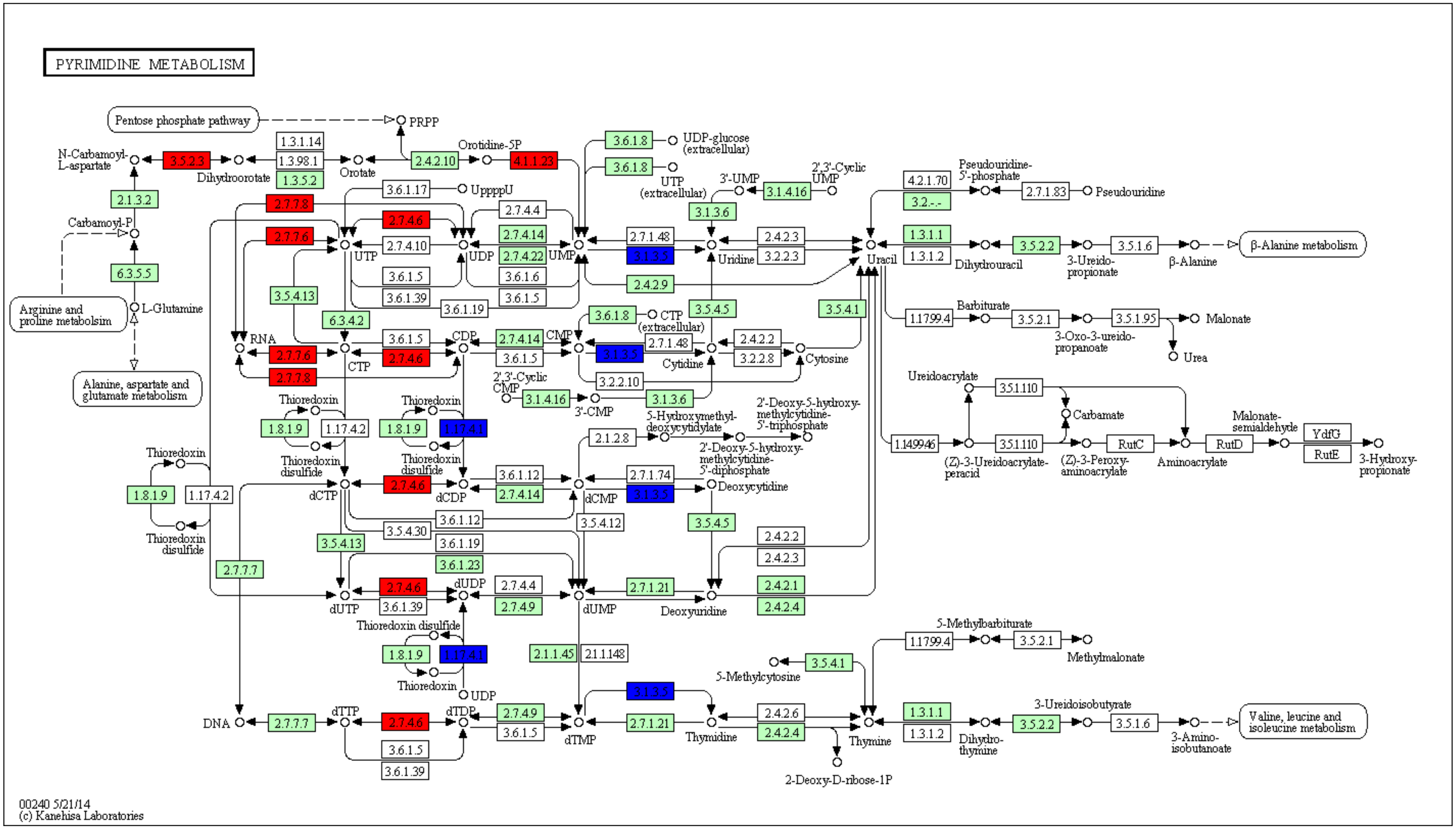
PYRIMIDINE METABOLISM
Pentose phosphate pathway
PRPP
N-Carbamoyl-L-aspartate
3.5.2.3
Dihydroorotate
1.3.1.14
1.3.98.1
1.3.5.2
Orotate
2.4.2.10
Orotidine-5P
4.1.1.23
2.1.3.2
Carbamoyl-P
6.3.5.5
L-Glutamine
Arginine and proline metabolsim
Alanine, aspartate and glutamate metabolism
2.7.7.8
2.7.7.6
3.6.1.17
UppppU
2.7.4.4
2.7.4.6
2.7.4.14
2.7.4.22
UTP
2.7.4.10
UDP
UMP
3.6.1.5
3.6.1.6
3.6.1.39
3.6.1.19
3.5.4.13
6.3.4.2
RNA
CTP
CDP
CMP
3.6.1.8
UDP-glucose (extracellular)
UTP (extracellular)
3'-UMP
3.1.4.16
2',3'-Cyclic UMP
3.1.3.6
2.7.1.48
3.1.3.5
2.4.2.3
3.2.2.3
Uridine
2.4.2.9
Uracil
3.5.4.5
3.5.4.1
CTP (extracellular)
2.4.2.2
3.2.2.8
Cytidine
Cytosine
3.2.2.10
2',3'-Cyclic CMP
3'-CMP
Pseudouridine-5'-phosphate
4.2.1.70
3.2.-.-
2.7.1.83
Pseudouridine
1.3.1.1
1.3.1.2
Dihydrouracil
3.5.2.2
3-Ureido-propionate
3.5.1.6
β-Alanine
β-Alanine metabolism
Barbiturate
1.17.99.4
3.5.2.1
3-Oxo-3-ureido-propanoate
3.5.1.95
Malonate
Urea
Ureidoacrylate
3.5.1.110
Carbamate
1.14.99.46
(Z)-3-Ureidoacrylate-peracid
(Z)-3-Peroxy-aminoacrylate
RutC
Aminoacrylate
RutD
Malonate-semialdehyde
YdfG
RutE
3-Hydroxy-propionate
Thioredoxin
Thioredoxin disulfide
1.8.1.9
1.17.4.2
1.17.4.1
5-Hydroxymethyl-deoxycytidylate
2'-Deoxy-5-hydroxy-methylcytidine-5'-triphosphate
2'-Deoxy-5-hydroxy-methylcytidine-5'-diphosphate
2.1.2.8
dCTP
dCDP
3.6.1.12
2.7.1.74
dCMP
Deoxycytidine
3.5.4.30
3.5.4.12
3.6.1.23
2.4.2.2
2.4.2.3
2.4.2.1
2.4.2.4
2.7.7.7
dUTP
dUDP
2.7.4.9
2.7.1.21
dUMP
Deoxyuridine
2.1.1.45
2.1.1.148
5-Methylbarbiturate
Methylmalonate
5-Methylcytosine
DNA
dTTP
dTDP
dTMP
Thymidine
2.4.2.6
Thymine
2-Deoxy-D-ribose-1P
3-Ureidoisobutyrate
Dihydro-thymine
3-Amino-isobutanoate
Valine, leucine and isoleucine metabolism
00240 5/21/14
(c) Kanehisa Laboratories

K

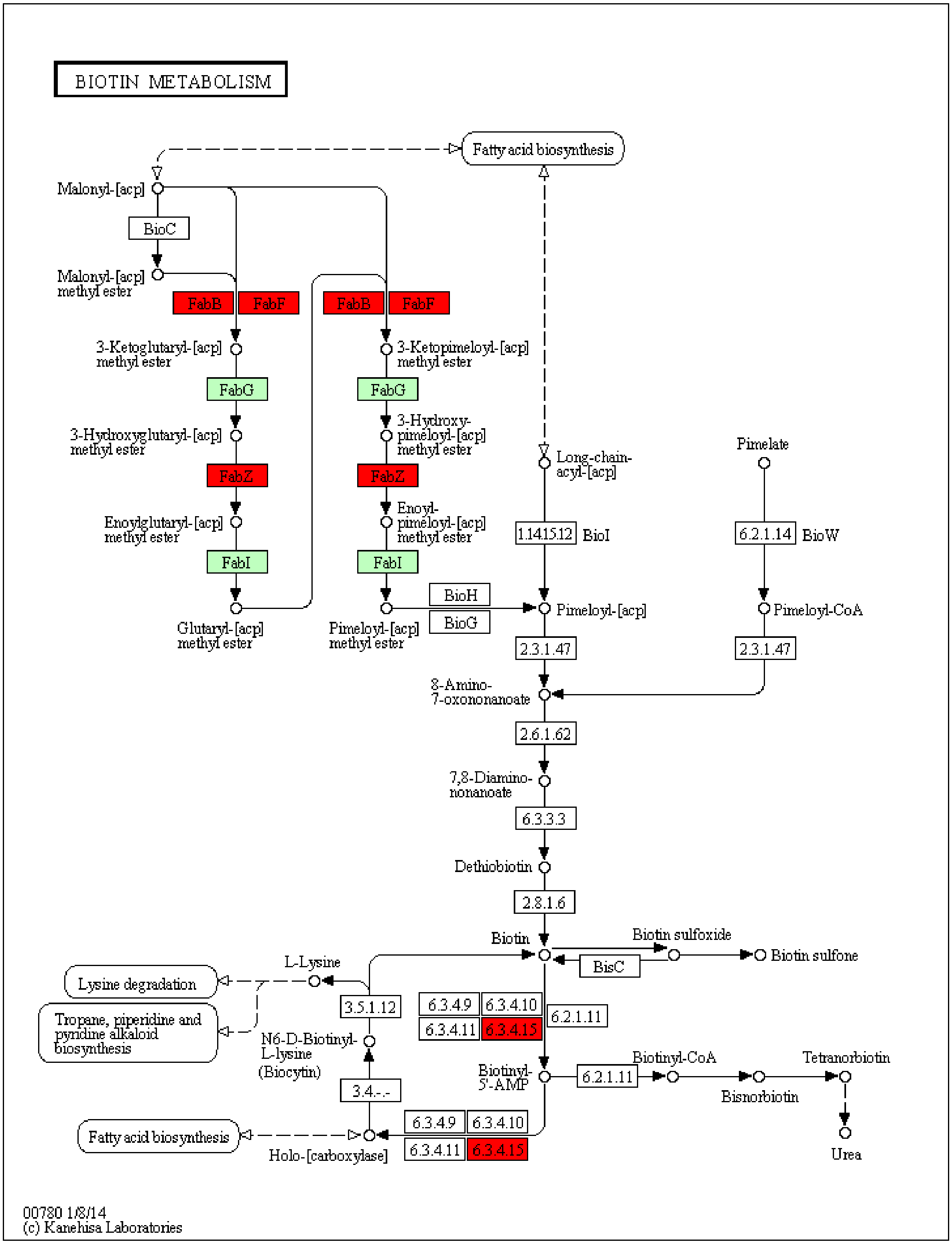

BIOTIN METABOLISM
Fatty acid biosynthesis
Malonyl-[acp]
BioC
Malonyl-[acp] methyl ester
FabB
FabF
FabB
FabF
3-Ketoglutaryl-[acp] methyl ester
3-Ketopimeloyl-[acp] methyl ester
FabG
FabG
3-Hydroxyglutaryl-[acp] methyl ester
3-Hydroxy-pimeloyl-[acp] methyl ester
FabZ
FabZ
Enoylglutaryl-[acp] methyl ester
Enoyl-pimeloyl-[acp] methyl ester
FabI
FabI
Glutaryl-[acp] methyl ester
Pimeloyl-[acp] methyl ester
BioH
BioG
Long-chain-acyl-[acp]
1.14.15.12
BioI
Pimelate
6.2.1.14
BioW
Pimeloyl-[acp]
Pimeloyl-CoA
2.3.1.47
2.3.1.47
8-Amino-7-oxononanoate
2.6.1.62
7,8-Diamino-nonanoate
6.3.3.3
Dethiobiotin
2.8.1.6
Biotin
Biotin sulfoxide
Biotin sulfone
BisC
L-Lysine
Lysine degradation
Tropane, piperidine and pyridine alkaloid biosynthesis
3.5.1.12
6.3.4.9
6.3.4.10
6.3.4.11
6.3.4.15
6.2.1.11
N6-D-Biotinyl-L-lysine (Biocytin)
3.4.-.-
Biotinyl-5'-AMP
6.2.1.11
Biotinyl-CoA
Bisnorbiotin
Tetranorbiotin
Urea
Fatty acid biosynthesis
6.3.4.9
6.3.4.10
6.3.4.11
6.3.4.15
Holo-[carboxylase]
00780 1/8/14
(c) Kanehisa Laboratories

L

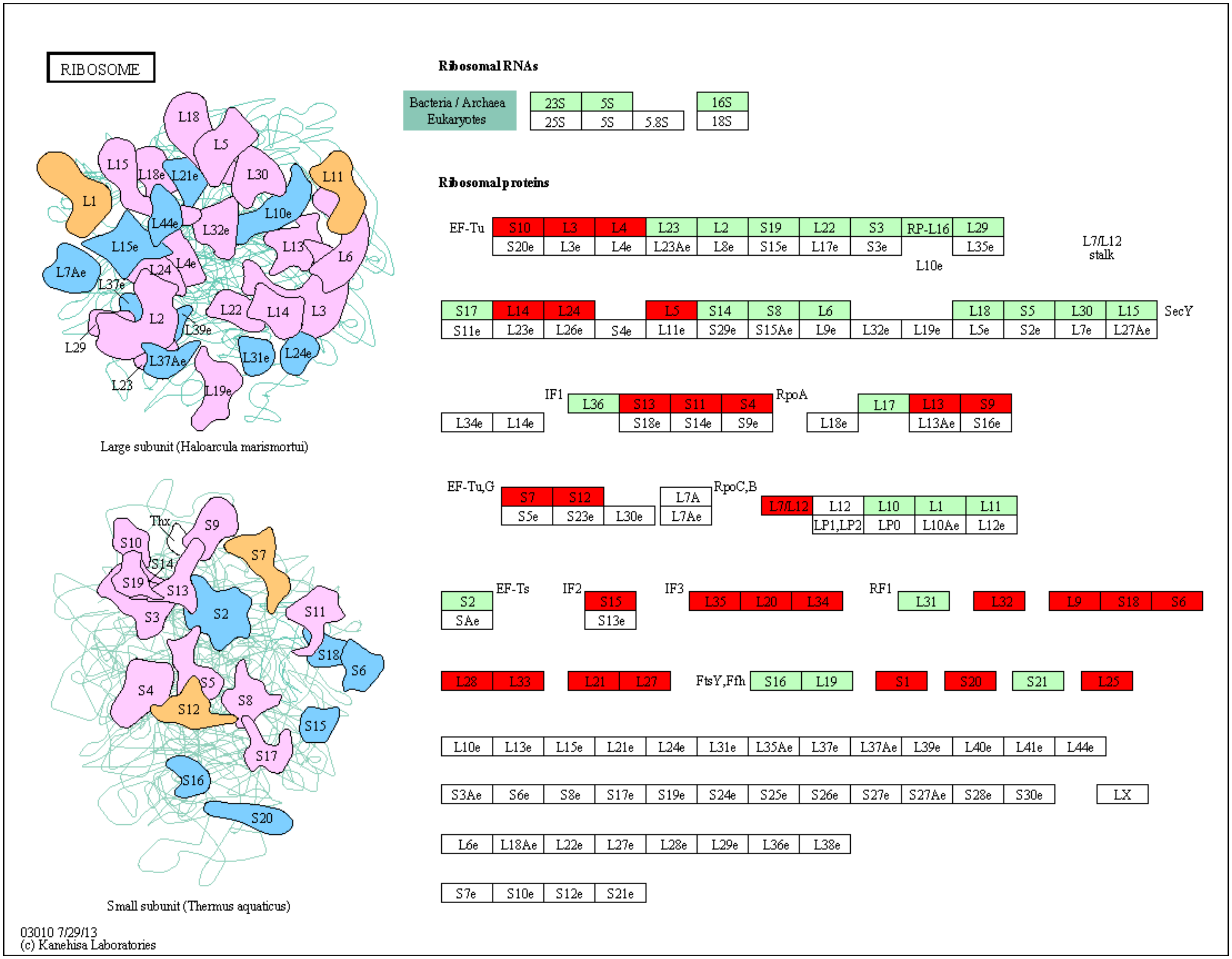
RIBOSOME
Ribosomal RNAs
Bacteria / Archaea
Eukaryotes
23S 5S 16S
25S 5S 5.8S 18S
Ribosomal proteins
EF-Tu
S10 L3 L4 L23 L2 S19 L22 S3 RP-L16 L29
S20e L3e L4e L23Ae L8e S15e L17e S3e L35e
L10e
L7/L12 stalk
S17 L14 L24 L5 S14 S8 L6 L18 S5 L30 L15 SecY
S11e L23e L26e S4e L11e S29e S15Ae L9e L32e L19e L5e S2e L7e L27Ae
IF1 L36 S13 S11 S4 RpoA L17 L13 S9
L34e L14e S18e S14e S9e L18e L13Ae S16e
EF-Tu,G S7 S12 L7A RpoC,B L7/L12 L12 L10 L1 L11
S5e S23e L30e L7Ae LP1,LP2 LP0 L10Ae L12e
S2 EF-Ts IF2 S15 IF3 L35 L20 L34 RF1 L31 L32 L9 S18 S6
SAe S13e
L28 L33 L21 L27 FtsY,Ffh S16 L19 S1 S20 S21 L25
L10e L13e L15e L21e L24e L31e L35Ae L37e L37Ae L39e L40e L41e L44e
S3Ae S6e S8e S17e S19e S24e S25e S26e S27e S27Ae S28e S30e LX
L6e L18Ae L22e L27e L28e L29e L36e L38e
S7e S10e S12e S21e
Large subunit (Haloarcula marismortui)
Small subunit (Thermus aquaticus)
03010 7/29/13
(c) Kanehisa Laboratories

M

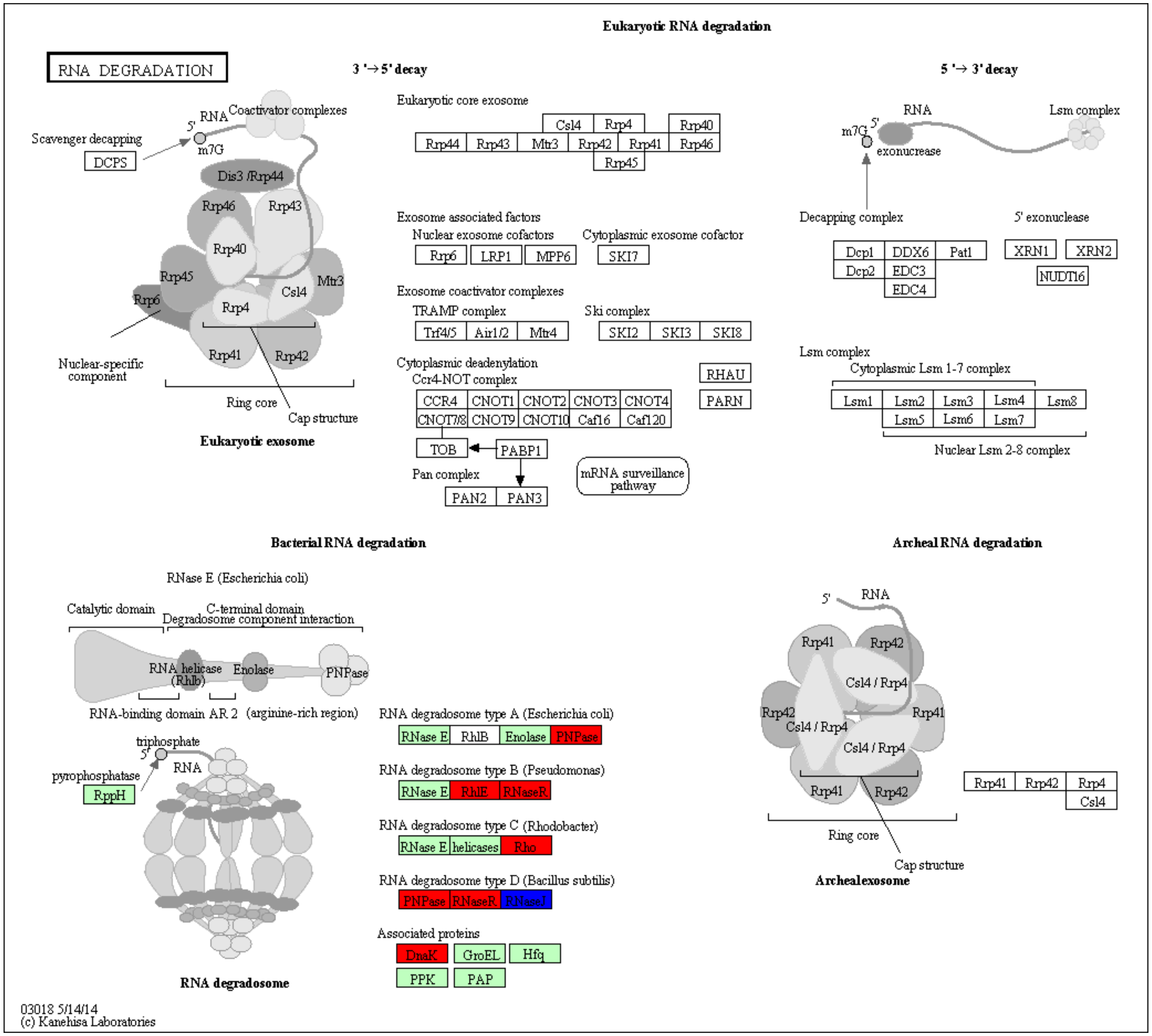

Eukaryotic RNA degradation
RNA DEGRADATION
3 '→5' decay
5 '→ 3' decay
RNA
5'
m7G
Coactivator complexes
Scavenger decapping
DCPS
Dis3 /Rrp44
Rrp46
Rrp43
Rrp40
Rrp45
Mtr3
Csl4
Rrp6
Rrp4
Rrp41
Rrp42
Nuclear-specific component
Ring core
Cap structure
Eukaryotic exosome
Eukaryotic core exosome
Csl4
Rrp4
Rrp40
Rrp44
Rrp43
Mtr3
Rrp42
Rrp41
Rrp46
Rrp45
Exosome associated factors
Nuclear exosome cofactors
Cytoplasmic exosome cofactor
Rrp6
LRP1
MPP6
SKI7
Exosome coactivator complexes
TRAMP complex
Ski complex
Trf4/5
Air1/2
Mtr4
SKI2
SKI3
SKI8
Cytoplasmic deadenylation
Ccr4-NOT complex
RHAU
PARN
CCR4
CNOT1
CNOT2
CNOT3
CNOT4
CNOT7/8
CNOT9
CNOT10
Caf16
Caf120
TOB
PABP1
Pan complex
mRNA surveillance pathway
PAN2
PAN3
RNA
Lsm complex
m7G
5'
exonucrease
Decapping complex
5' exonuclease
Dcp1
DDX6
Pat1
XRN1
XRN2
Dcp2
EDC3
NUDT16
EDC4
Lsm complex
Cytoplasmic Lsm 1-7 complex
Lsm1
Lsm2
Lsm3
Lsm4
Lsm8
Lsm5
Lsm6
Lsm7
Nuclear Lsm 2-8 complex
Bacterial RNA degradation
Archeal RNA degradation
RNase E (Escherichia coli)
Catalytic domain
C-terminal domain
Degradosome component interaction
RNA helicase (Rhlb)
Enolase
PNPase
RNA-binding domain AR 2 (arginine-rich region)
triphosphate
5'
RNA
pyrophosphatase
RppH
RNA degradosome
RNA degradosome type A (Escherichia coli)
RNase E
RhlB
Enolase
PNPase
RNA degradosome type B (Pseudomonas)
RNase E
RhlE
RNaseR
RNA degradosome type C (Rhodobacter)
RNase E
helicases
Rho
RNA degradosome type D (Bacillus subtilis)
PNPase
RNaseR
RNaseJ
Associated proteins
DnaK
GroEL
Hfq
PPK
PAP
5'
RNA
Rrp41
Rrp42
Csl4 / Rrp4
Rrp42
Rrp41
Csl4 / Rrp4
Csl4 / Rrp4
Rrp41
Rrp42
Ring core
Cap structure
Archealexosome
Rrp41
Rrp42
Rrp4
Csl4
03018 5/14/14
(c) Kanehisa Laboratories

N

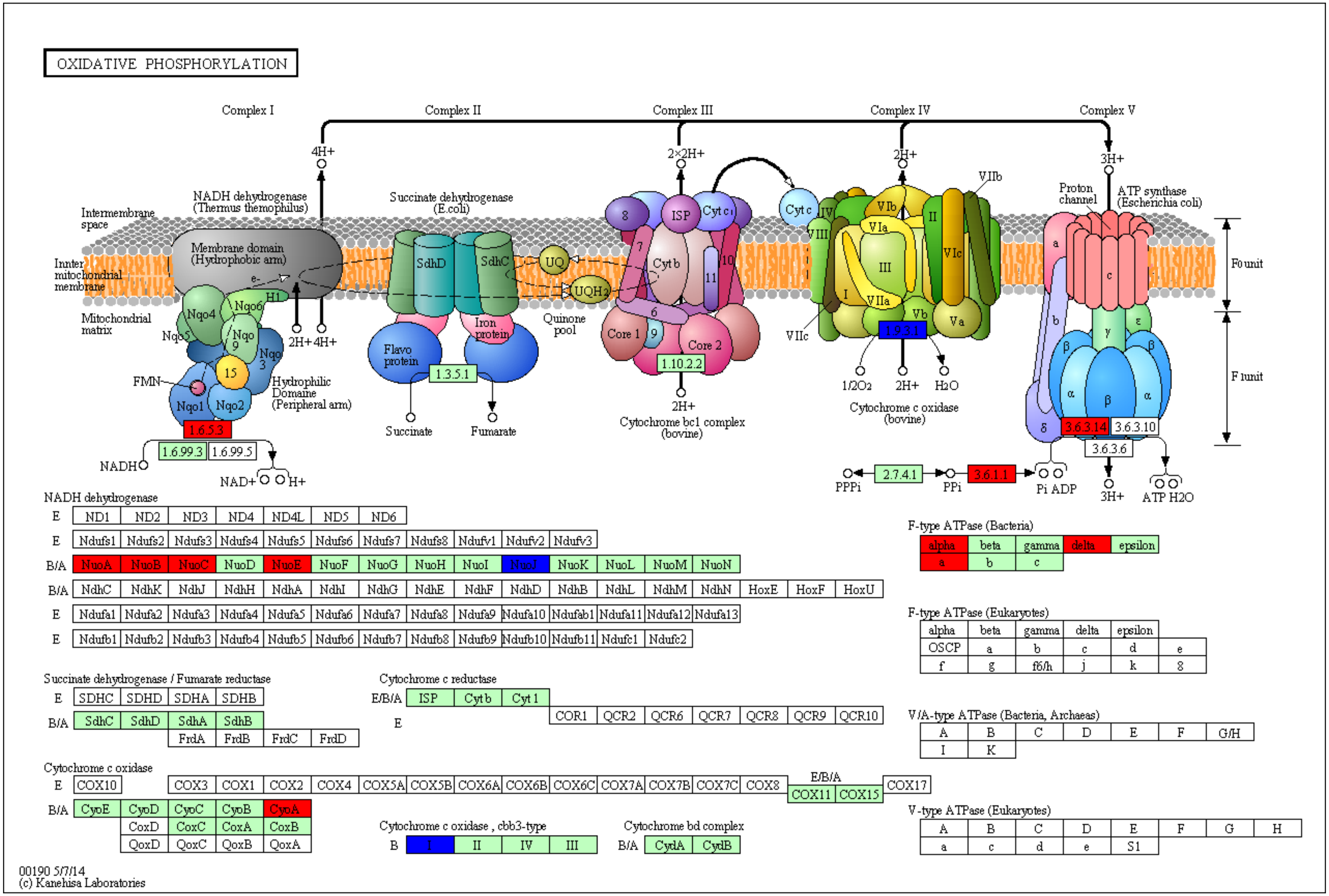
OXIDATIVE PHOSPHORYLATION
Complex I
Complex II
Complex III
Complex IV
Complex V
4H+
2×2H+
2H+
3H+
NADH dehydrogenase (Thermus thermophilus)
Succinate dehydrogenase (E.coli)
Intermembrane space
Inner mitochondrial membrane
Mitochondrial matrix
Membrane domain (Hydrophobic arm)
e-
H1
Nqo4
Nqo6
Nqo5
Nqo9
Nqo3
15
FMN
Nqo1
Nqo2
Hydrophilic Domaine (Peripheral arm)
2H+ 4H+
1.6.5.3
1.6.99.3
1.6.99.5
NADH
NAD+
H+
SdhD
SdhC
UQ
UQH2
Flavo protein
Iron protein
Quinone pool
1.3.5.1
Succinate
Fumarate
8
ISP
Cyt c1
Cyt c
7
Cyt b
10
11
6
Core 1
9
Core 2
1.10.2.2
2H+
Cytochrome bc1 complex (bovine)
VIIb
IV
VIII
VIb
VIa
II
VIc
III
I
VIIa
Vb
Va
VIIc
1.9.3.1
1/2O2
2H+
H2O
Cytochrome c oxidase (bovine)
Proton channel
ATP synthase (Escherichia coli)
a
c
b
ε
γ
β
α
δ
F0 unit
F1 unit
3.6.3.14
3.6.3.10
3.6.3.6
PPPi
2.7.4.1
PPi
3.6.1.1
Pi ADP
3H+
ATP H2O
NADH dehydrogenase
E: ND1 ND2 ND3 ND4 ND4L ND5 ND6
E: Ndufs1 Ndufs2 Ndufs3 Ndufs4 Ndufs5 Ndufs6 Ndufs7 Ndufs8 Ndufv1 Ndufv2 Ndufv3
B/A: NuoA NuoB NuoC NuoD NuoE NuoF NuoG NuoH NuoI NuoJ NuoK NuoL NuoM NuoN
B/A: NdhC NdhK NdhJ NdhH NdhA NdhI NdhG NdhE NdhF NdhD NdhB NdhL NdhM NdhN HoxE HoxF HoxU
E: Ndufa1 Ndufa2 Ndufa3 Ndufa4 Ndufa5 Ndufa6 Ndufa7 Ndufa8 Ndufa9 Ndufa10 Ndufab1 Ndufa11 Ndufa12 Ndufa13
E: Ndufb1 Ndufb2 Ndufb3 Ndufb4 Ndufb5 Ndufb6 Ndufb7 Ndufb8 Ndufb9 Ndufb10 Ndufb11 Ndufc1 Ndufc2
Succinate dehydrogenase / Fumarate reductase
E: SDHC SDHD SDHA SDHB
B/A: SdhC SdhD SdhA SdhB
FrdA FrdB FrdC FrdD
Cytochrome c reductase
E/B/A: ISP Cytb Cyt1
E: COR1 QCR2 QCR6 QCR7 QCR8 QCR9 QCR10
Cytochrome c oxidase
E: COX10 COX3 COX1 COX2 COX4 COX5A COX5B COX6A COX6B COX6C COX7A COX7B COX7C COX8
E/B/A: COX11 COX15
COX17
B/A: CyoE CyoD CyoC CyoB CyoA
CoxD CoxC CoxA CoxB
QoxD QoxC QoxB QoxA
Cytochrome c oxidase, cbb3-type
B: I II IV III
Cytochrome bd complex
B/A: CydA CydB
F-type ATPase (Bacteria)
alpha beta gamma delta epsilon
a b c
F-type ATPase (Eukaryotes)
alpha beta gamma delta epsilon
OSCP a b c d e
f g f6/h j k 8
V/A-type ATPase (Bacteria, Archaeas)
A B C D E F G/H
I K
V-type ATPase (Eukaryotes)
A B C D E F G H
a c d e S1
00190 5/7/14
(c) Kanehisa Laboratories

O

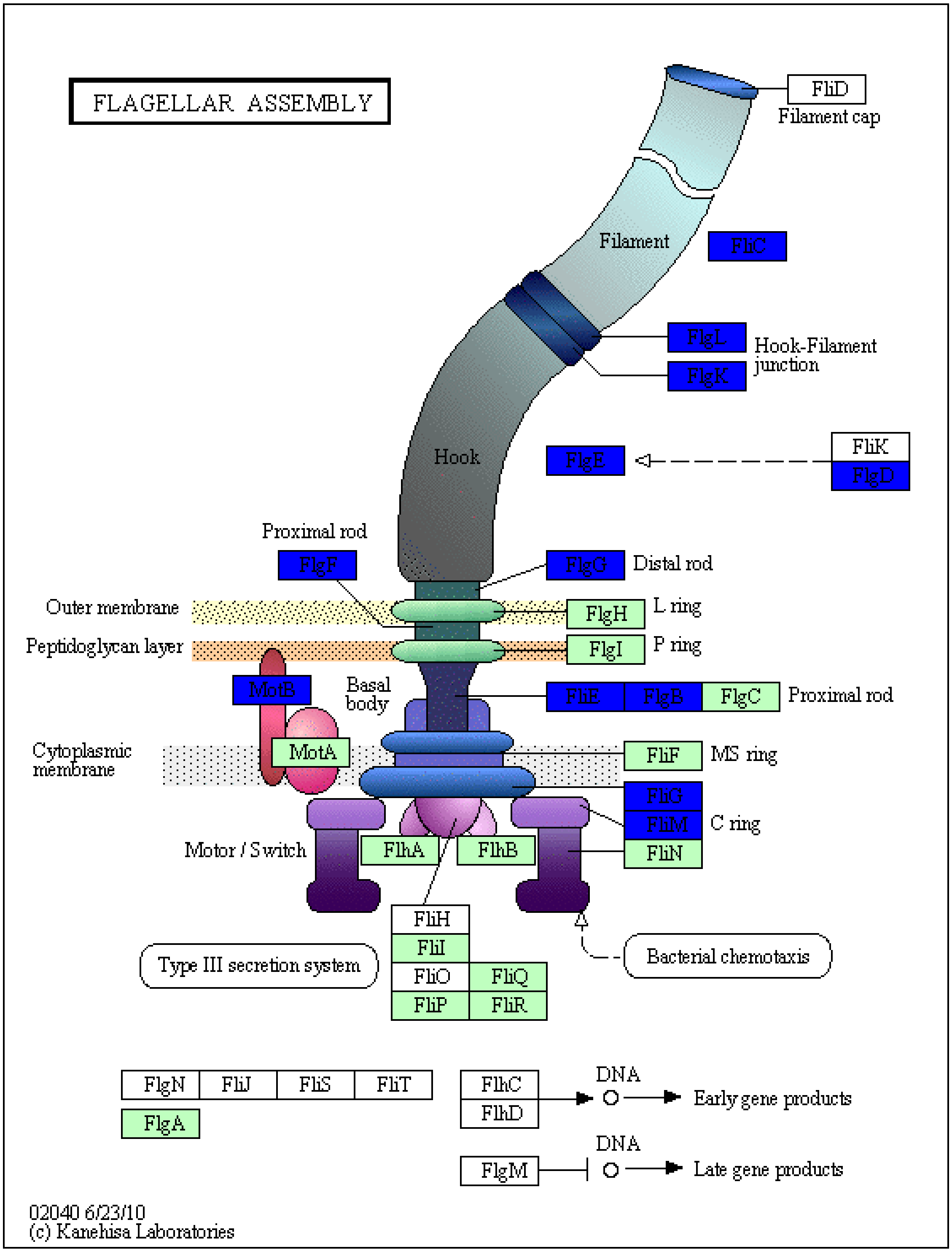
FLAGELLAR ASSEMBLY
FliD
Filament cap
Filament
FliC
FlgL
FlgK
Hook-Filament junction
Hook
FlgE
FliK
FlgD
Proximal rod
FlgF
FlgG
Distal rod
Outer membrane
FlgH
L ring
Peptidoglycan layer
FlgI
P ring
MotB
Basal body
FliE
FlgB
FlgC
Proximal rod
Cytoplasmic membrane
MotA
FliF
MS ring
FliG
FliM
FliN
C ring
Motor / Switch
FlhA
FlhB
FliH
FliI
FliO
FliQ
FliP
FliR
Type III secretion system
Bacterial chemotaxis
FlgN
FliJ
FliS
FliT
FlgA
FlhC
FlhD
DNA
Early gene products
FlgM
DNA
Late gene products
02040 6/23/10
(c) Kanehisa Laboratories

P

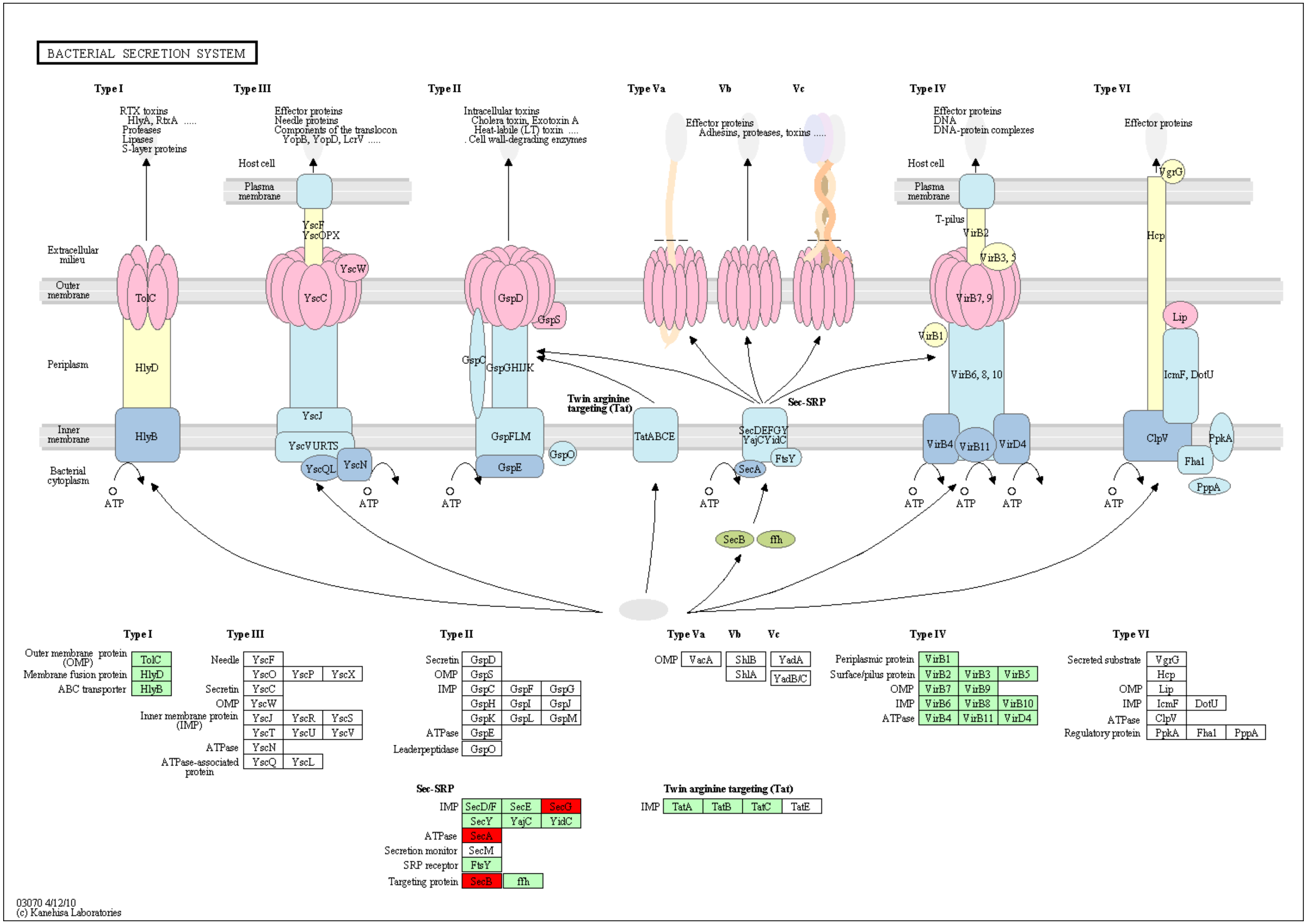
BACTERIAL SECRETION SYSTEM
Type I
Type III
Type II
Type Va
Vb
Vc
Type IV
Type VI
RTX toxins
HlyA, RtxA .....
Proteases
Lipases
S-layer proteins
Effector proteins
Needle proteins
Components of the translocon
YopB, YopD, LcrV .....
Intracellular toxins
Cholera toxin, Exotoxin A
Heat-labile (LT) toxin ....
Cell wall-degrading enzymes
Effector proteins
Adhesins, proteases, toxins .....
Effector proteins
DNA
DNA-protein complexes
Effector proteins
Host cell
Plasma membrane
Extracellular milieu
Outer membrane
Periplasm
Inner membrane
Bacterial cytoplasm
TolC
HlyD
HlyB
YscF
YscOPX
YscW
YscC
YscJ
YscVURTS
YscQL
YscN
GspD
GspS
GspC
GspGHIJK
GspFLM
GspE
GspO
Twin arginine targeting (Tat)
TatABCE
Sec-SRP
SecDEFGY
YajCYidC
FtsY
SecA
SecB
ffh
T-pilus
VirB2
VirB3, 5
VirB7, 9
VirB1
VirB6, 8, 10
VirB4
VirB11
VirD4
VgrG
Hcp
Lip
IcmF, DotU
ClpV
PpkA
FhaI
PppA
ATP
Type I
Outer membrane protein (OMP) TolC
Membrane fusion protein HlyD
ABC transporter HlyB
Type III
Needle YscF
YscO YscP YscX
Secretin YscC
OMP YscW
Inner membrane protein (IMP) YscJ YscR YscS
YscT YscU YscV
ATPase YscN
ATPase-associated protein YscQ YscL
Type II
Secretin GspD
OMP GspS
IMP GspC GspF GspG
GspH GspI GspJ
GspK GspL GspM
ATPase GspE
Leaderpeptidase GspO
Type Va Vb Vc
OMP VacA ShlB YadA
ShlA YadB/C
Type IV
Periplasmic protein VirB1
Surface/pilus protein VirB2 VirB3 VirB5
OMP VirB7 VirB9
IMP VirB6 VirB8 VirB10
ATPase VirB4 VirB11 VirD4
Type VI
Secreted substrate VgrG
Hcp
OMP Lip
IMP IcmF DotU
ATPase ClpV
Regulatory protein PpkA FhaI PppA
Sec-SRP
IMP SecD/F SecE SecG
SecY YajC YidC
ATPase SecA
Secretion monitor SecM
SRP receptor FtsY
Targeting protein SecB ffh
Twin arginine targeting (Tat)
IMP TatA TatB TatC TatE
03070 4/12/10
(c) Kanehisa Laboratories

Q

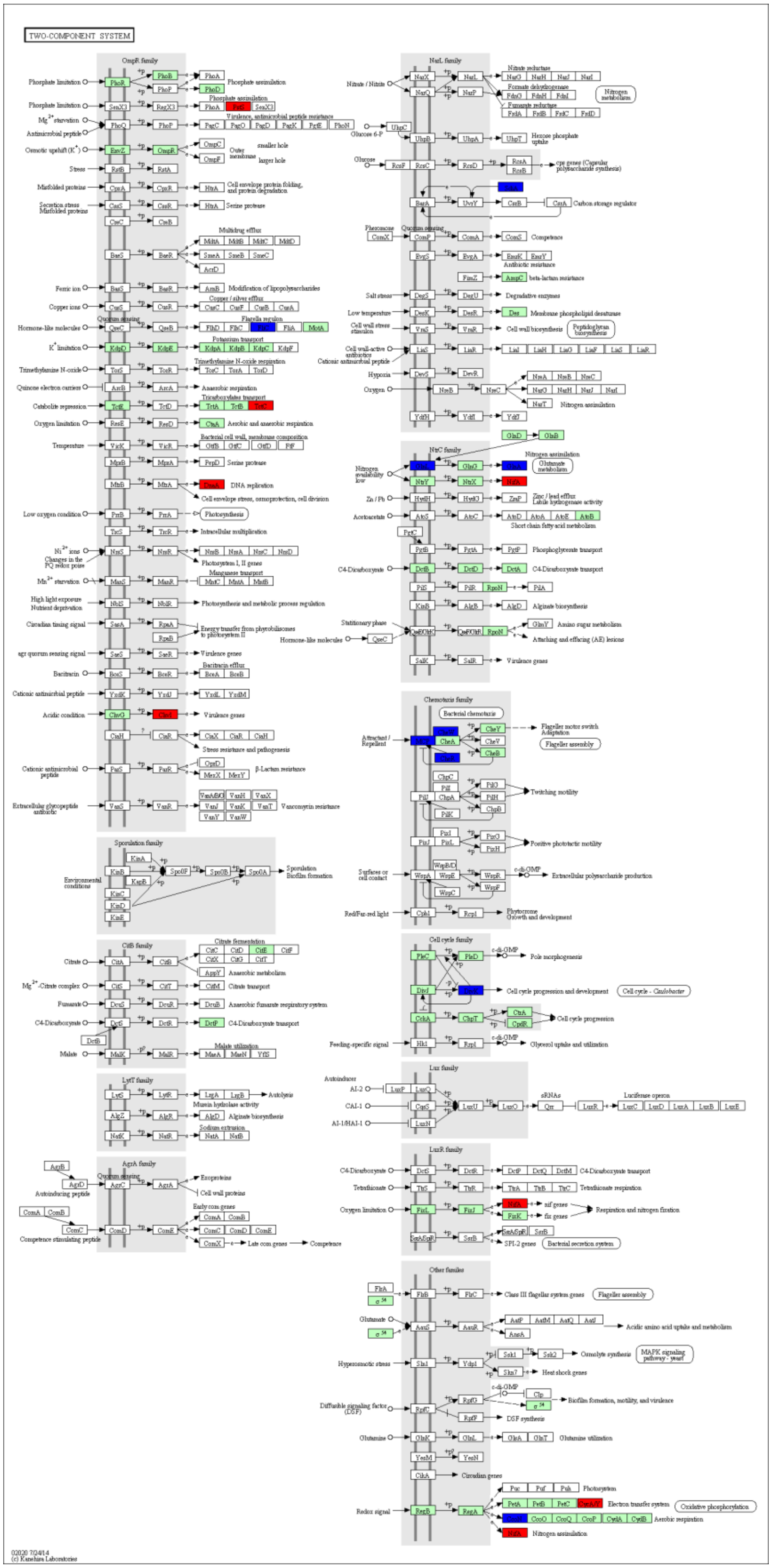
TWO-COMPONENT SYSTEM
OmpR family
NarL family
NtrC family
Chemotaxis family
Sporulation family
CitB family
Cell cycle family
LytT family
Lux family
AgrA family
LuxR family
Other families
02020 7/24/14
(c) Kanehisa Laboratories

R

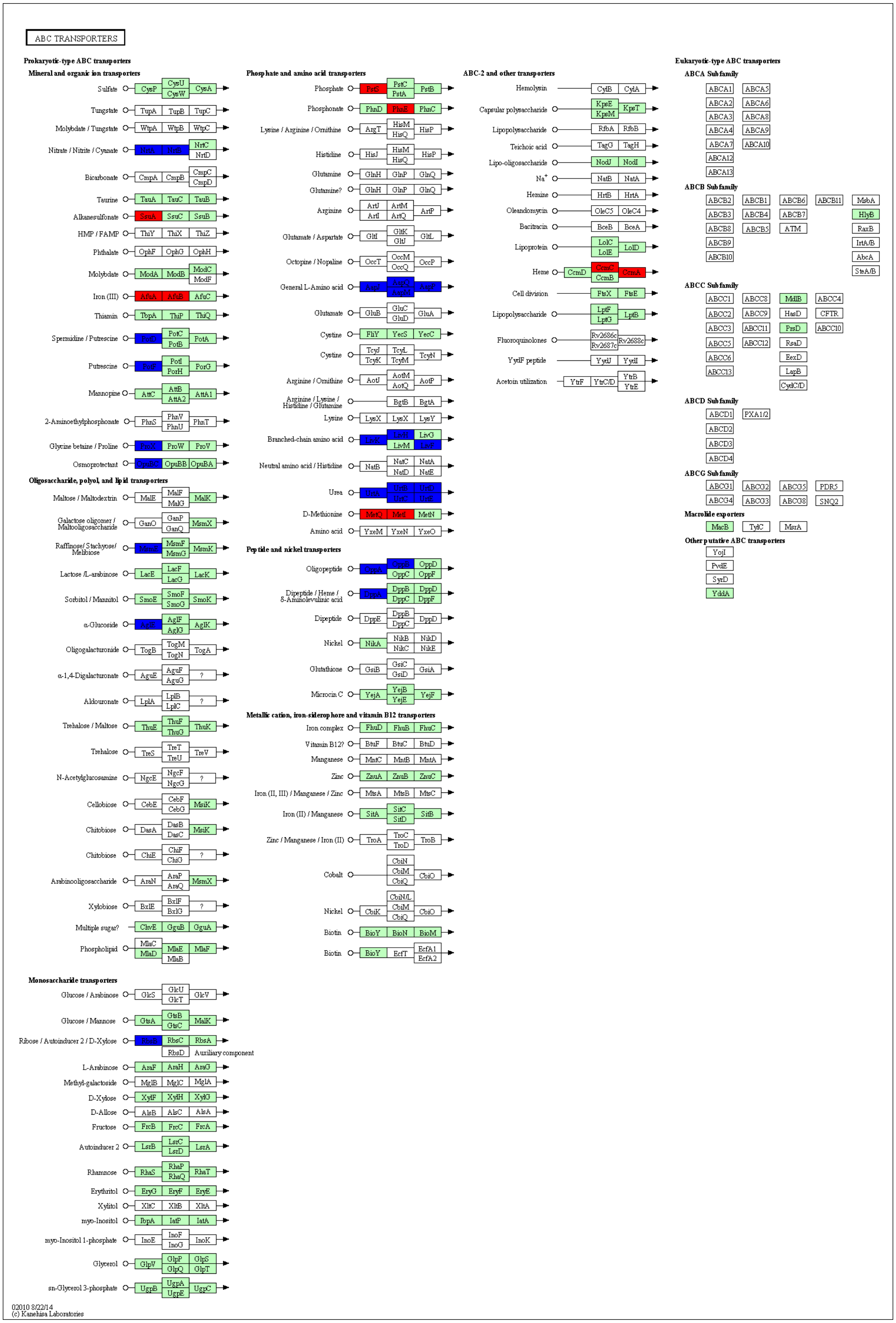

ABC TRANSPORTERS
Prokaryotic-type ABC transporters
Mineral and organic ion transporters
Sulfate CysP CysU CysW CysA
Tungstate TupA TupB TupC
Molybdate / Tungstate WtpA WtpB WtpC
Nitrate / Nitrite / Cyanate NrtC NrtD
Bicarbonate CmpA CmpB CmpC CmpD
Taurine TauA TauC TauB
Alkanesulfonate SsuC SsuB
HMP / FAMP ThiY ThiX ThiZ
Phthalate OphF OphG OphH
Molybdate ModA ModB ModC ModF
Iron (III) AfuA AfuB AfuC
Thiamin TbpA ThiP ThiQ
Spermidine / Putrescine PotC PotB PotA
Putrescine PotI PotH PotG
Mannopine AttC AttB AttA2 AttA1
2-Aminoethylphosphonate PhnS PhnV PhnU PhnT
Glycine betaine / Proline ProW ProV
Osmoprotectant OpuBB OpuBA
Oligosaccharide, polyol, and lipid transporters
Maltose / Maltodextrin MalE MalF MalG MalK
Galactose oligomer / Maltooligosaccharide GanO GanP GanQ MsmX
Raffinose/ Stachyose/ Melibiose MsmF MsmG MsmK
Lactose /L-arabinose LacE LacF LacG LacK
Sorbitol / Mannitol SmoE SmoF SmoG SmoK
α-Glucoside AglF AglG AglK
Oligogalacturonide TogB TogM TogN TogA
α-1,4-Digalacturonate AguE AguF AguG ?
Aldouronate LplA LplB LplC ?
Trehalose / Maltose ThuE ThuF ThuG ThuK
Trehalose TreS TreT TreU TreV
N-Acetylglucosamine NgcE NgcF NgcG ?
Cellobiose CebE CebF CebG MsiK
Chitobiose DasA DasB DasC MsiK
Chitobiose ChiE ChiF ChiG ?
Arabinooligosaccharide AraN AraP AraQ MsmX
Xylobiose BxlE BxlF BxlG ?
Multiple sugar? ChvE GguB GguA
Phospholipid MlaC MlaD MlaE MlaB MlaF
Monosaccharide transporters
Glucose / Arabinose GlcS GlcU GlcT GlcV
Glucose / Mannose GtsA GtsB GtsC MalK
Ribose / Autoinducer 2 / D-Xylose RbsC RbsA RbsD Auxiliary component
L-Arabinose AraF AraH AraG
Methyl-galactoside MglB MglC MglA
D-Xylose XylF XylH XylG
D-Allose AlsB AlsC AlsA
Fructose FrcB FrcC FrcA
Autoinducer 2 LsrB LsrC LsrD LsrA
Rhamnose RhaS RhaP RhaQ RhaT
Erythritol EryG EryF EryE
Xylitol XltC XltB XltA
myo-Inositol IbpA IatP IatA
myo-Inositol 1-phosphate InoE InoF InoG InoK
Glycerol GlpV GlpP GlpQ GlpS GlpT
sn-Glycerol 3-phosphate UgpB UgpA UgpE UgpC
Phosphate and amino acid transporters
Phosphate PstC PstA PstB
Phosphonate PhnD PhnC
Lysine / Arginine / Ornithine ArgT HisM HisQ HisP
Histidine HisJ HisM HisQ HisP
Glutamine GlnH GlnP GlnQ
Glutamine? GlnH GlnP GlnQ
Arginine ArtJ ArtI ArtM ArtQ ArtP
Glutamate / Aspartate GltI GltK GltJ GltL
Octopine / Nopaline OccT OccM OccQ OccP
General L-Amino acid
Glutamate GluB GluC GluD GluA
Cystine FliY YecS YecC
Cystine TcyJ TcyK TcyL TcyM TcyN
Arginine / Ornithine AotJ AotM AotQ AotP
Arginine / Lysine / Histidine / Glutamine BgtB BgtA
Lysine LysX LysX LysY
Branched-chain amino acid LivM LivG
Neutral amino acid / Histidine NatB NatC NatD NatA NatE
Urea
D-Methionine MetN
Amino acid YxeM YxeN YxeO
Peptide and nickel transporters
Oligopeptide OppC OppD OppF
Dipeptide / Heme / δ-Aminolevulinic acid DppB DppC DppD DppF
Dipeptide DppE DppB DppC DppD
Nickel NikA NikB NikC NikD NikE
Glutathione GsiB GsiC GsiD GsiA
Microcin C YejA YejB YejE YejF
Metallic cation, iron-siderophore and vitamin B12 transporters
Iron complex FhuD FhuB FhuC
Vitamin B12? BtuF BtuC BtuD
Manganese MntC MntB MntA
Zinc ZnuA ZnuB ZnuC
Iron (II, III) / Manganese / Zinc MtsA MtsB MtsC
Iron (II) / Manganese SitA SitC SitD SitB
Zinc / Manganese / Iron (II) TroA TroC TroD TroB
Cobalt CbiN CbiM CbiQ CbiO
Nickel CbiK CbiN/L CbiM CbiQ CbiO
Biotin BioY BioN BioM
Biotin BioY EcfT EcfA1 EcfA2
ABC-2 and other transporters
Hemolysin CylB CylA
Capsular polysaccharide KpsE KpsM KpsT
Lipopolysaccharide RfbA RfbB
Teichoic acid TagG TagH
Lipo-oligosaccharide NodJ NodI
Na^+ NatB NatA
Hemine HrtB HrtA
Oleandomycin OleC5 OleC4
Bacitracin BceB BceA
Lipoprotein LolC LolE LolD
Heme CcmD CcmC CcmB CcmA
Cell division FtsX FtsE
Lipopolysaccharide LptF LptG LptB
Fluoroquinolones Rv2686c Rv2687c Rv2688c
YydF peptide YydJ YydI
Acetoin utilization YtrF YtrC/D YtrB YtrE
Eukaryotic-type ABC transporters
ABCA Subfamily
ABCA1 ABCA5 ABCA2 ABCA6 ABCA3 ABCA8 ABCA4 ABCA9 ABCA7 ABCA10 ABCA12 ABCA13
ABCB Subfamily
ABCB2 ABCB1 ABCB6 ABCB11 MsbA ABCB3 ABCB4 ABCB7 HlyB ABCB8 ABCB5 ATM RaxB ABCB9 IrtA/B ABCB10 AbcA SteA/B
ABCC Subfamily
ABCC1 ABCC8 MdlB ABCC4 ABCC2 ABCC9 HasD CFTR ABCC3 ABCC11 PrsD ABCC10 ABCC5 ABCC12 RsaD ABCC6 EexD ABCC13 LapB CydC/D
ABCD Subfamily
ABCD1 PXA1/2 ABCD2 ABCD3 ABCD4
ABCG Subfamily
ABCG1 ABCG2 ABCG5 PDR5 ABCG4 ABCG3 ABCG8 SNQ2
Macrolide exporters
MacB TylC MsrA
Other putative ABC transporters
YojI PvdE SyrD YddA
02010 3/22/14
(c) Kanehisa Laboratories
