# Supplementary material for: A consolidated analysis of the physiologic and molecular responses induced under acid stress in the legume-symbiont model-soil bacterium Sinorhizobium meliloti
Source: Sci Rep. 2016 Jul 11;6:29278. doi: 10.1038/srep29278 (PMC4941405; doi:10.1038/srep29278)
Supplement: Supplementary Dataset 2 [file srep29278-s2.pdf]

A consolidated analysis of the physiologic and molecular responses induced under acid stress in the legume-symbiont model-soil bacterium *Sinorhizobium meliloti*.

Draghi, W. O.[1]; Del Papa, M. F. [1]; Hellweg, C.[2]; Watt, S. A.[2]; Watt, T. F.[2]; Barsch, A[2]. Lozano, M. J.[1]; Lagares, A. (Jr.)[4]; Salas, M. E[1]; López, J. L.[1]; Albicoro, F. J.[1]; Nilson, J. F.[1]; Torres Tejerizo, G. A.[1]; Luna, M. F. [3]; Pistorio, M.[1]; Boiardi, J. L.[3]; Pühler, A.[2]; Weidner; S.[2]; Niehaus, K.[2]; and Lagares A. [1]*

[1] IBBM - Instituto de Biotecnología y Biología Molecular, CONICET - Departamento de Ciencias Biológicas, Facultad de Ciencias Exactas, Universidad Nacional de La Plata, calles 47 y 115, 1900-La Plata, Argentina

[2] CeBiTec - Centrum für Biotechnologie, Universität Bielefeld, Bielefeld, Germany

[3] CINDEFI – Centro de Investigación y Desarrollo en Fermentaciones Industriales, CONICET - Departamento de Química, Facultad de Ciencias Exactas, Universidad Nacional de La Plata, calles 47 y 115, 1900-La Plata, Argentina

[4] Laboratorio de Bioquímica, Microbiología e Interacciones Biológicas en el Suelo, Departamento de Ciencia y Tecnología, Universidad Nacional de Quilmes, Roque Sáenz Peña 352, Bernal B1876BXD, Buenos Aires, Argentina

Key words: acid stress, rhizobia, legume

***Corresponding author**
Phone: +54-221-425-0497 ext. 31
Fax: +54-221-422-3409 ext. 56
E-mail:lagares@biol.unlp.edu.ar

Supplementary Table 1. mRNA transcripts induced in acid chemostat cultures. Values are ordered by M-values (M>1.58).

ORFName	Gene	M-Value	P-Value	A-Value	COG Function
SMa1262	-	1.58548904	6.6536E-07	11.589882	General function prediction only
SMa2408	*alcA*	1.59611731	0.00014554	8.34364176	Secondary metabolites biosynthesis
SMc02652	*rnc*	1.60666928	7.881E-06	8.39098757	Transcription
SMa0815	*nifA*	1.61104863	9.0315E-09	11.2389603	Transcription Signal transduction mechanisms
SMc02499	*atpA*	1.61189688	3.5167E-07	12.1172341	Energy production and conversion
SMb20204	*pqqA*	1.63121328	2.0677E-09	12.9390689	Not in COGs
SMc02940	*yaeN*	1.63396401	2.1335E-06	11.0011781	Cell cycle control
SMc01299	*rplN*	1.63755784	3.436E-10	13.9868273	Translation
SMc02146	*pstS*	1.63895592	1.3539E-08	10.3010513	Inorganic ion transport and metabolism
SMc02435	*hemK*	1.64232368	2.3632E-07	11.1607666	Translation
SMc00714	*plsC*	1.64762275	4.3503E-08	9.50681642	Lipid transport and metabolism
SMa0851	*glpD*	1.64878712	5.4278E-06	8.83733124	Not in COGs
SMa1207	*dnr*	1.65074116	2.1521E-09	9.77307533	Signal transduction mechanisms
SMc04094	-	1.65324515	3.294E-11	10.5318432	Function unknown
SMc01784	*plsX*	1.65542888	2.7463E-10	11.1278399	Lipid transport and metabolism
SMa0163	*rhcC2*	1.65807817	6.1101E-08	11.5715085	Intracellular trafficking
SMb21189	*lgtD*	1.66324295	8.1016E-09	12.2021789	Cell Wall
SMc03961	*sqdB*	1.67367763	5.0054E-08	11.3813073	Cell wall Carbohydrate transport and metabolism
SMc04277	*fabZ*	1.68043692	2.0426E-09	11.1445314	Lipid transport and metabolism
SMb20552	*gbpD*	1.68976668	8.9158E-12	14.1351932	General function prediction only
SMa0633	*ywaD*	1.69280641	3.8435E-07	9.63030511	Not in COGs
SMa0848	-	1.69379288	1.4781E-07	10.8045351	Not in COGs
SMc02495	*tal*	1.6987857	1.8854E-09	11.2053186	Carbohydrate transport and metabolism
SMc01152	*rpsT*	1.70560369	3.5147E-06	11.4636977	Translation
SMc02408	*rnpO*	1.71128929	3.0665E-10	11.5210901	Transcription
SMc00186	-	1.71227873	1.5971E-09	10.8435777	Defense mechanisms

Supplementary Table 1. mRNA transcripts induced in acid chemostat cultures. Values are ordered by M-values (M>1.58) (continued).

ORFName	*Gene*	M-Value	P-Value	A-Value	COG Function
SMb21295	*ibpA*	1.71624612	7.8491E-76	9.70269968	Posttranslational modification
SMc00458	*phoP*	1.72021917	1.898E-09	11.2598127	Signal transduction mechanisms Transcription
SMc01844	-	1.72425225	4.5826E-10	9.31321083	Carbohydrate transport and metabolism Amino acid transport and metabolism General function prediction only
SMc03881	*rpmF*	1.72454849	8.0501E-09	10.4659343	Translation
SMc02160	-	1.72769843	2.0224E-09	11.4521501	General function prediction only
SMc00340	*kguD*	1.72781013	1.2431E-06	10.149035	Amino acid transport and metabolism
SMb21516	-	1.72970806	9.1608E-05	9.98017181	Function unknown
SMc02847	*pan*	1.7341864	1.2939E-07	10.4061813	General function prediction only
SMa1179	*nosR*	1.73716439	5.9531E-06	10.8097133	Transcription
SMc02242	-	1.73944422	3.7767E-06	9.95087774	Not in COGs
SMc03773	-	1.74083749	6.998E-08	10.1060028	Translation
SMb20364	*afuB*	1.74415835	2.3791E-07	10.5953971	Inorganic ion transport and metabolism
SMc03847	*ccmA*	1.75879981	2.7494E-08	10.7837017	Posttranslational modification
SMc01765	-	1.7589209	1.2229E-09	12.3495481	Function unknown
SMc00725	*argH*	1.76003048	1.3865E-07	9.25664494	Amino acid transport and metabolism
SMc00743	-	1.76135526	6.5816E-08	11.6420969	General function prediction only
SMc00088	-	1.76932097	3.0234E-07	9.77864069	Not in COGs
SMc00568	*rpsF*	1.77340838	9.7325E-09	15.0548857	Translation
SMa1776	-	1.78208017	1.6694E-10	11.4471249	Function unknown
SMa1573	*ctpF*	1.79083006	3.4408E-08	12.1031189	Intracellular trafficking
SMc02854	-	1.79785853	1.4632E-09	9.59392957	Not in COGs
SMc00153	-	1.80141303	1.5469E-08	11.2929695	Function unknown
SMc03877	*mgpS*	1.80241283	9.7306E-09	11.0999487	Replication Transcription Translation
SMc03795	*leuD*	1.81568775	6.338E-10	11.7182454	Amino acid transport and metabolism
SMc01458	-	1.81707972	2.7203E-08	10.8066607	Cell Wall
SMc03172	*gltX*	1.82232227	5.8738E-07	9.41125924	Translation

Supplementary Table 1. mRNA transcripts induced in acid chemostat cultures. Values are ordered by M-values (M>1.58) (continued).

ORFName	*Gene*	M-Value	P-Value	A-Value	COG Function
SMb20989	*hflC*	1.8337062	5.3566E-11	14.4940582	Posttranslational modification
SMc00335	*rpsA*	1.86097983	8.905E-180	12.4298527	Translation
SMb21009	*glpK*	1.86327712	2.8921E-06	8.1923785	Energy production and conversion
SMc02692	*rplY*	1.86869095	2.0148E-09	11.4769038	Translation
SMb21175	*phnE*	1.87226692	9.7593E-06	9.16836781	Inorganic ion transport and metabolism
SMb20353	*ida*	1.87869926	3.4759E-09	13.0454888	General function prediction only
SMc03772	*rpmA*	1.88630814	5.7149E-10	11.1127087	Translation
SMc01513	*hmuS*	1.88837001	2.3742E-07	9.59808693	Inorganic ion transport and metabolism
SMc02278	-	1.90030015	3.017E-08	10.089171	Not in COGs
SMc03070	*zwf*	1.90177682	2.6429E-08	12.8426324	Carbohydrate transport and metabolism
SMc00280	-	1.9092699	2.3396E-09	12.2690889	Not in COGs
SMb20920	*htr8*	1.91166465	4.153E-10	12.2190615	Not in COGs
SMc02580	*hyuA*	1.9119882	4.8436E-11	12.8460196	Amino acid transport and metabolism Secondary metabolites biosynthesis
SMc01914	*nuoC*	1.9164944	3.1152E-09	10.4118907	Energy production and conversion
SMc00960	*tldD*	1.91878312	1.3023E-09	12.09047	General function prediction only
SMc00485	*rpsD*	1.92030632	7.9846E-08	11.2509876	Translation
SMb20754	-	1.92099003	2.0953E-09	13.2867666	General function prediction only
SMc01861	*murE*	1.9278184	1.1845E-07	9.41720828	Cell Wall
SMa1266	*hemN*	1.95374362	5.6914E-09	8.1951981	Coenzyme transport and metabolism
SMc01774	*carB*	1.96335101	2.2415E-07	14.9881615	Not in COGs
SMc02434	-	1.96482026	1.7012E-08	12.0489034	Not in COGs
SMc02723	-	1.96645362	8.2126E-07	8.07771216	General function prediction only
SMc00712	-	1.9677443	2.9652E-07	10.8446119	Function unknown
SMa1128	*htrA*	1.96867045	1.7848E-09	10.9339964	Posttranslational modification
SMc00324	*pnp*	1.97083763	8.6482E-11	11.9721467	Translation
SMc02432	-	1.97642878	1.6702E-10	9.4728603	Cell Wall
SMc02365	*dop*	1.97802733	1.7601E-08	12.920852	Posttranslational modification

Supplementary Table 1. mRNA transcripts induced in acid chemostat cultures. Values are ordered by M-values (M>1.58) (continued).

ORFName	*Gene*	M-Value	P-Value	A-Value	COG Function
SMb21505	*exoQ*	1.9794309	1.1248E-12	12.5826702	Cell Wall
SMc01026	*recJ-like*	2.00162427	4.463E-06	11.7774381	Not in COGs
SMc04267	*nodH*	2.0051896	3.3065E-06	8.7728241	Function unknown
SMc00522	*rhlE*	2.00550708	1.9818E-11	12.4130613	Replication Transcription Translation
SMc00092	*cysH*	2.01838442	5.6151E-07	10.0786159	Amino acid transport and metabolism Coenzyme transport and metabolism
SMa0760	*fixT1*	2.01966309	4.091E-09	9.98705815	Signal transduction mechanisms
SMa1296	*adhP*	2.03681958	2.5231E-09	9.85717951	General function prediction only
SMb21518	*amiC*	2.05968016	7.248E-08	9.76162349	Not in COGs
SMa1170	*coxM*	2.06474012	1.61E-09	10.1719019	Energy production and conversion
SMc00815	*guaB*	2.08083691	1.051E-10	9.5094357	Nucleotide transport and metabolism
SMc02349	*frdA*	2.10336064	1.892E-08	9.29736269	Energy production and conversion
SMc02052	-	2.10659565	4.7741E-10	13.7267537	Not in COGs
SMc00349	*lepA*	2.11038278	2.3696E-11	10.7332912	Cell Wall
SMc00062	-	2.12651004	2.759E-10	10.0586615	Function unknown
SMc00929	*gcvA*	2.13165304	1.2383E-07	10.696463	Transcription
SMa0849	*syrM*	2.14579821	1.4983E-08	8.59723487	Transcription
SMc01913	*nuoB*	2.16256873	1.2241E-10	12.8162302	Energy production and conversion
SMa1220	*fixN*	2.17602433	2.6643E-13	10.2595307	Energy production and conversion
SMa1082	-	2.17879589	7.101E-11	13.8071505	Not in COGs
SMc00483	*murI*	2.1831555	1.8449E-09	9.09631641	Cell Wall
SMc01910	*yhaN*	2.18401738	5.8631E-10	10.9711717	Function unknown
SMc00363	*rpmI*	2.1851894	2.149E-176	11.7115846	Translation
SMc03831	-	2.18809666	4.3008E-08	8.3598153	General function prediction only
SMc01314	*rpsL*	2.20656093	1.191E-06	12.2038182	Translation
SMc01580	*ydaH*	2.23360987	1.8605E-09	11.4905337	Not in COGs
SMb20186	-	2.23478655	2.8475E-08	9.31085783	Function unknown

Supplementary Table 1. mRNA transcripts induced in acid chemostat cultures. Values are ordered by M-values (M>1.58) (continued).

ORFName	*Gene*	M-Value	P-Value	A-Value	COG Function
SMa2073	*acd*	2.2381681	1.6872E-06	8.27207909	Lipid transport and metabolism
SMa1211	*fixG*	2.24663493	1.3055E-11	11.194584	Energy production and conversion
SMc04411	-	2.25117563	3.7428E-08	10.9314709	Cell Wall
SMc00007	*aroC*	2.26571808	1.6922E-09	9.10586702	Amino acid transport and metabolism
SMa1243	*azu1*	2.33823412	6.1603E-13	13.6674307	Energy production and conversion
SMc00331	-	2.37393529	6.1404E-09	8.17181182	Function unknown
SMc00690	*accA*	2.38198883	3.3534E-11	10.851773	Lipid transport and metabolism
SMc00332	-	2.39183692	1.2072E-09	10.4192665	Function unknown
SMc02305	*murA*	2.40381945	2.3944E-10	10.7902713	Cell Wall
SMa1077	*nex18*	2.42590077	2.5445E-15	12.5162321	Cell Wall
SMb21440	-	2.44227378	3.7807E-10	12.7405598	Not in COGs
SMc01718	-	2.44515381	4.31E-10	11.2952843	Function unknown
SMb20880	*rhlE*	2.47527439	1.1079E-11	12.902477	Replication Transcription Translation
SMc01341	-	2.47652358	6.1068E-12	10.1460719	Not in COGs
SMc01912	*nuoA*	2.51419105	1.3449E-08	12.082341	Energy production and conversion
SMc02266	-	2.56144284	1.3452E-10	10.924509	Not in COGs
SMa2075	-	2.57806859	0.00265956	9.93639692	Amino acid transport and metabolism
SMc00364	*rplT*	2.58074563	9.0335E-11	10.9507509	Translation
SMa0763	-	2.63124341	1.3827E-11	10.8928482	General function prediction only
SMc02897	*cycM*	2.64861469	3.8028E-07	10.158078	Energy production and conversion
SMc01313	*rpsG*	2.71057062	1.5495E-12	11.3717463	Translation
SMc00567	*rpsR*	2.74219768	1.0247E-08	10.5131972	Translation
SMc00950	-	2.98966637	3.3295E-13	11.1952996	General function prediction only
SMc03239	*ppa*	3.00297141	2.1002E-12	11.5187615	Energy production and conversion
SMa0045	*cah*	3.22485924	1.6534E-11	9.45772375	Inorganic ion transport and metabolism
SMc03242	*typA*	3.3801664	3.3699E-11	9.33349902	Signal transduction mechanisms
SMc01855	*slt*	3.57531647	3.3829E-10	10.7774849	Cell wall
SMc02900	-	3.79119397	3.4822E-09	11.9605937	Not in COGs

Supplementary Table 1. . mRNA transcripts repressed in acid chemostat cultures. Values are ordered by M-values (M< -1.58) (continued).

ORFName	Gene	M-Value	P-Value	A-Value	COG Function
SMb20605	-	-4.8237228	2.8144E-11	12.842455	Amino acid transport and metabolism
SMb20984	*nirB*	-4.60196563	1.0679E-07	10.0935251	Energy production and conversion
SMa1683	*aslA1*	-4.58393294	3.1606E-11	11.574338	Inorganic ion transport and metabolism
SMc03806	*glnK*	-4.5559039	1.4036E-10	13.0318113	Amino acid transport and metabolism
SMa0585	*nrtA*	-4.24410458	4.7281E-09	13.1821797	Inorganic ion transport and metabolism
SMb20886	-	-4.20488909	0.00046482	10.1170777	Function unknown
SMb20436	*narK*	-4.19954839	2.533E-09	10.2347374	Inorganic ion transport and metabolism
SMb20604	-	-3.99228824	5.6104E-09	9.62795438	Amino acid transport and metabolism
SMc03807	*amtB*	-3.94014173	1.9428E-09	11.4229338	Inorganic ion transport and metabolism
SMc03049	*flgL*	-3.91922003	7.4077E-08	9.37431295	Cell motility
SMc03037	*flaD*	-3.2183134	1.0523E-08	12.0556974	Cell motility
SMc03030	*flgG*	-3.17920173	2.3094E-05	8.90353865	Cell motility
SMa2361	-	-3.04849128	1.5749E-07	9.35299126	General function prediction only
SMc00979	-	-2.86230151	1.8468E-08	9.36507761	Not in COGs
SMc02240	-	-2.85637038	7.7598E-12	11.9075516	Function unknown
SMc02118	*aapJ*	-2.81539955	3.3282E-12	13.4518406	Amino acid transport and metabolism Signal transduction mechanisms
SMa1635	*cgmA*	-2.79636394	5.4478E-13	12.7300604	Not in COGs
SMa0392	*potD_2*	-2.75958006	1.2701E-08	11.0969597	Amino acid transport and metabolism
SMb20745	*glnII*	-2.71897977	3.3381E-11	13.0684081	Amino acid transport and metabolism
SMc03038	*flaD*	-2.67047572	2.7626E-07	11.6799569	Cell motility
SMc03027	*flgB*	-2.63290948	4.7024E-09	9.01862498	Cell motility
SMc02119	*aapQ*	-2.60828229	3.5717E-12	12.4646164	Amino acid transport and metabolism
SMc04114	*ctpA*	-2.605422	2.8414E-07	13.5354024	Intracellular trafficking
SMa0612	*fixN*	-2.59772954	0.00028612	10.2399307	Energy production and conversion
SMc01946	*livK*	-2.58926532	2.3189E-09	14.1116802	Amino acid transport and metabolism
SMc01827	-	-2.58805018	1.2323E-09	11.5358971	Inorganic ion transport and metabolism
SMc03834	*phhB*	-2.58255582	8.8064E-08	10.1806492	Coenzyme transport and metabolism

Supplementary Table 1. . mRNA transcripts repressed in acid chemostat cultures. Values are ordered by M-values (M< -1.58) (continued).

ORFName	Gene	M-Value	P-Value	A-Value	COG Function
SMb20915	*atsA*	-2.45630288	4.2672E-09	10.8073034	Inorganic ion transport and metabolism
SMa0583	*nrtB*	-2.41411749	1.9613E-06	9.74479903	Inorganic ion transport and metabolism
SMb20985	*nirD*	-2.36799963	2.5737E-05	9.41529441	Inorganic ion transport and metabolism General function prediction only
SMc03029	*fliE*	-2.34080057	9.2718E-08	9.70935776	Cell motility Intracellular trafficking
SMc04043	-	-2.31265869	6.15E-09	10.6379739	Amino acid transport and metabolism
SMc02120	*aapM*	-2.28895803	5.1589E-10	11.103403	Amino acid transport and metabolism
SMb21115	*nasT*	-2.2121726	5.0404E-06	10.34089	Signal transduction mechanisms
SMc01930	*gloA*	-2.15397819	2.4275E-07	11.6599722	Amino acid transport and metabolism
SMb20806	-	-2.13318033	9.2651E-09	11.753991	Not in COGs
SMb20651	*acpA*	-2.10742731	2.9264E-08	9.10940818	Lipid transport and metabolism Secondary metabolites biosynthesis
SMc03880	-	-2.07669752	3.2174E-09	11.6234648	Function unknown
SMa1233	*napB*	-2.05371381	2.1471E-06	9.54292997	Energy production and conversion
SMa1706	-	-2.04580646	2.874E-06	9.36455873	Not in COGs
SMa0941	*nodW*	-2.02461237	0.00013025	8.26770281	Signal transduction mechanisms
SMa1961	-	-2.0174378	2.1461E-07	11.2995703	Secondary metabolites biosynthesis
SMc03039	*flaD*	-1.99479981	5.8005E-07	9.67272913	Cell motility
SMb20811	*cshA*	-1.9712435	6.7253E-07	11.2599296	Not in COGs
SMa2245	-	-1.97033852	1.434E-07	9.78404671	General function prediction only
SMc00360	-	-1.96454914	1.588E-09	11.6491685	Function unknown
SMc01947	*cadB*	-1.95966645	2.2094E-08	11.9139712	Not in COGs
SMa0937	-	-1.93323631	1.4168E-05	10.3920301	Cell wall
SMa1857	*hadL*	-1.9283666	0.00525663	8.56611387	General function prediction only
SMb21315	*prsE*	-1.92468524	1.1009E-06	9.59601543	Cell wall
SMa0943	*atsA*	-1.89514853	3.3537E-06	10.867285	Inorganic ion transport and metabolism
SMc03009	*cheR*	-1.8555164	4.0665E-08	8.42072154	Cell motility Signal transduction mechanisms
SMb20909	-	-1.83954682	1.0283E-06	8.24024037	Not in COGs
SMc03044	*motD*	-1.83586073	6.4398E-07	8.36513411	Not in COGs

Supplementary Table 1. . mRNA transcripts repressed in acid chemostat cultures. Values are ordered by M-values (M< -1.58) (continued).

ORFName	Gene	M-Value	P-Value	A-Value	COG Function
SMb20652	*ansB*	-1.80067998	3.4077E-08	11.4703576	Amino acid transport and metabolism
SMc02111	-	-1.78727719	2.6202E-09	13.8189215	Not in COGs
SMc00283	*mexT*	-1.76438867	7.9696E-05	8.52914592	Transcription
SMa1507	-	-1.75619242	1.1271E-07	9.67937287	General function prediction only
SMb21254	-	-1.74864717	5.7334E-09	11.2005776	General function prediction only
SMc00997	*etfA*	-1.72953706	3.5548E-06	9.876769	Not in COGs
SMb21572	-	-1.7162345	1.5515E-07	11.4600786	Amino acid transport and metabolism
SMa0391	-	-1.70425054	2.1856E-07	9.05746455	Amino acid transport and metabolism
SMc00770	*potF*	-1.70315162	6.674E-08	12.0791701	Amino acid transport and metabolism
SMc02079	*fadL*	-1.68221923	2.4521E-07	10.8569764	Lipid transport and metabolism
SMc00737	-	-1.67848298	6.6394E-08	11.1413804	Defense mechanisms
SMc01951	*livH*	-1.67481286	1.3215E-07	10.4286473	Amino acid transport and metabolism
SMc00999	*kdsB*	-1.67157414	1.0418E-05	9.62400882	Not in COGs
SMb21117	*phrR*	-1.6188859	1.2264E-06	11.0244833	Transcription
SMc01967	*speB2*	-1.61021818	6.9183E-09	9.02266091	Amino acid transport and metabolism
SMc04018	*yfkN*	-1.59481426	7.8728E-10	11.4769443	Nucleotide transport and metabolism
SMc03046	-	-1.59367719	1.2038E-06	10.4382086	Signal transduction mechanisms Transcription
SMb20650	*fadD*	-1.58780509	1.0688E-09	11.4749924	Lipid transport and metabolism Secondary metabolites biosynthesis
SMb21314	*rzcA*	-1.58727006	1.323E-07	11.4624031	Secondary metabolites biosynthesis
SMc03021	*fliM*	-1.58141084	3.3752E-07	8.56630938	Cell motility
SMc02121	*aapP*	-1.58021391	1.5804E-06	10.5553539	Amino acid transport and metabolism

Supplementary Table 2. List of metabolites detected by GC-MS and analysed by MetabolAnalyst.

2-Aminoadipate	2-Hydroxyglutarate	2-Isopropylmalate	4-Aminobutyrate	5-Methylthio-adenosine	Adenine
Adenosine	a-Ketoaminobutyrate	a-Ketoglutarate	Alanine	Arabinose	Arginin-NH3
b-Alanine	Citrate	Cytosine	D/L-Diaminopimelate	DHAP	Erythrose4P
Fructose_6_P	Fructose	Fumarate	Galactose	Gluconate	Gluconate-1,5-lactone
Gluconate-6-P	Gluconic	Acid	Glucose-6-P	Glutamine	Glycerate
Glycerol-3-P	Glycine	Isocitrate	Isoleucine	L-Cystathionine	L-Cysteine
Leucine	L-Homoserine	Malate	Mannitol	Mannose-6P	Melibiose
Methionine	myo-Inositol	N-Acetyl-Glutamate	Norleucine	O-Acetyl_L_Homoserine	O-Acetyl-L-Serine
Ornithin_Arginine_Citrulline	Pep	Phenylalanine	Proline	Putrescine	Pyruvate
Ribose-5P	Ribulose-5P	Spermidine	Succinate	Sucrose	Threonine
Thymine	Trehalose	Tryptophane	Tyrosine	Uracile	Urea
Valine.					

Supplementary Table 3. Metabolites with fold change (FC) either ≥ 2 (increased at pH 6.1) or ≤ 0.5 (increased at pH 7.0) ($p \leq 0.05$) (see legend to the Volcano Plot in manuscript Fig. 3).

Compound	FC	$\log_2$(FC)	*p*-value	$\log_{10}$(p)
Spermidine	30.74	4.94	7.73E-07	6.11
beta-Alanine	5.57	2.48	8.38E-08	7.08
Ornithine_Arginine_Citrulline	3.94	1.98	1.92E-07	6.71
2-Aminoadipate	3.80	1.92	3.14E-06	5.50
Gluconate-1,5-lactone	3.56	1.83	8.94E-08	7.05
Glucose-6-P	2.42	1.28	8.74E-06	5.06
Fructose	2.42	1.27	3.19E-04	3.50
Norleucine	2.41	1.27	1.86E-06	5.73
O-Acetyl_L_Homoserine	2.39	1.25	6.37E-04	3.20
4-Aminobutyrate	2.37	1.24	2.73E-06	5.56
Pep	2.36	1.24	7.45E-03	2.13
N-Acetyl-Glutamate	2.31	1.21	2.79E-03	2.55
Galactose	2.29	1.19	4.77E-07	6.32
O-Acetyl-L-Serine	0.49	-1.01	4.10E-05	4.39
Phenylalanine	0.45	-1.15	2.58E-05	4.59
L-Cysteine	0.38	-1.38	6.65E-04	3.18
Melibiose	0.28	-1.85	7.75E-02	1.11
2-Isopropylmalate	0.08	-3.61	5.01E-09	8.30
2-Hydroxyglutarate	0.04	-4.48	1.62E-07	6.79
